# Specific decorations of 17-hydroxygeranyllinalool diterpene glycosides solve the autotoxicity problem of chemical defense in *Nicotiana attenuata*

**DOI:** 10.1093/plcell/koab048

**Published:** 2021-02-09

**Authors:** Sven Heiling, Lucas Cortes Llorca, Jiancai Li, Klaus Gase, Axel Schmidt, Martin Schäfer, Bernd Schneider, Rayko Halitschke, Emmanuel Gaquerel, Ian Thomas Baldwin

**Affiliations:** 1 Department of Molecular Ecology, Max Planck Institute for Chemical Ecology, 07745 Jena, Germany; 2 Department of Biochemistry, Max Planck Institute for Chemical Ecology, 07745 Jena, Germany; 3 Research Group Biosynthesis/NMR, Max Planck Institute for Chemical Ecology, 07745 Jena, Germany; 4 Centre for Organismal Studies Heidelberg, 69120 Heidelberg, Germany; 5 Institut de Biologie Moléculaire des Plantes, CNRS UPR 2357 Université de Strasbourg, 67084 Strasbourg, France

## Abstract

The native diploid tobacco *Nicotiana attenuata* produces abundant, potent anti-herbivore defense metabolites known as 17-hydroxygeranyllinalool diterpene glycosides (HGL-DTGs) whose glycosylation and malonylation biosynthetic steps are regulated by jasmonate signaling. To characterize the biosynthetic pathway of HGL-DTGs, we conducted a genome-wide analysis of uridine diphosphate glycosyltransferases (UGTs) and identified 107 family-1 UGT members. The transcript levels of three UGTs were highly correlated with the transcript levels two key HGL-DTG biosynthetic genes: geranylgeranyl diphosphate synthase (Na*GGPPS)* and geranyllinalool synthase (Na*GLS*). NaGLS’s role in HGL-DTG biosynthesis was confirmed by virus-induced gene silencing. Silencing the Uridine diphosphate (UDP)-rhamnosyltransferase gene *UGT91T1* demonstrated its role in the rhamnosylation of HGL-DTGs. In vitro enzyme assays revealed that UGT74P3 and UGT74P4 use UDP-glucose for the glucosylation of 17-hydroxygeranyllinalool (17-HGL) to lyciumoside I. Plants with stable silencing of *UGT74P3* and *UGT74P5* were severely developmentally deformed, pointing to a phytotoxic effect of the aglycone. The application of synthetic 17-HGL and silencing of the UGTs in HGL-DTG-free plants confirmed this phytotoxic effect. Feeding assays with tobacco hornworm (*Manduca sexta*) larvae revealed the defensive functions of the glucosylation and rhamnosylation steps in HGL-DTG biosynthesis. Glucosylation of 17-HGL is therefore a critical step that contributes to the resulting metabolites’ defensive function and solves the autotoxicity problem of this potent chemical defense.

## Teaser

Glycosylation reactions eliminate the toxic effects of 17-hydroxygeranyllinalool diterpene glycosides to plants while contributing to the defensive functions of these compounds during herbivore attack.

S.H. designed and performed the research, analyzed metabolomics and transcriptome data, annotated the UGTs, screened the transient and stable lines, performed the phytotoxicity, enzymatic, and performance assays, developed the quantitative method for 17-HGL and wrote the article. L.C.L. assisted with the expression data. J.L. performed the enzymatic assay. K.G. designed the constructs for the transiently and stably silenced lines. A.S. assisted with the in vitro enzyme activity assay. M.S. assisted with the development of the quantitative 17-HGL method and analysis of phytohormones and general metabolites. B. S. performed NMR measurements and verified the synthetized 17-HGL mixture. E. G. and I.T.B. designed the research and wrote the article. R.H. wrote the article.

## Introduction

During the course of evolution, plants have developed versatile defense strategies against biotic stressors, such as herbivores and pathogens. Among these defensive strategies are physical and chemical barriers such as trichomes (Fordyce and Agrawal, 2001), volatiles that attract predators (Kessler and Baldwin, 2001; Halitschke et al., 2008), and a wide array of defensive specialized metabolites. Many small molecules produced as part of a plant’s specialized metabolism function as direct defense compounds by being toxic, repellent, or anti-nutritive for herbivores of different feeding guilds and degrees of specialization; compounds with a broad spectrum of toxicity (e.g. glucosinolates, alkaloids, and terpenoids) are likely to be of greater defensive value against a greater diversity of attackers. However, these broad-spectrum toxins force the producers to solve the “toxic waste dump problem” of chemical defense, as many direct defense compounds are generally cytotoxic and can damage the tissues of nonadapted producers.

Plants have evolved numerous ways of solving this “toxic waste dump problem” for broad-spectrum chemical defenses. One frequently used solution is glycosylation, which is one of the most prevalent and widespread biochemical modifications contributing to the structural and functional diversity of specialized metabolites in plants. The incorporation of sugar molecules into small lipophilic metabolites can regulate the storage/localization of defensive metabolites (Gachon et al., 2005; Yadav et al., 2014) and change their bioactivity by detoxifying phytotoxic intermediates. For example, for the defensive deployment of hydrogen cyanide (Gleadow and Moller, 2014) or steroidal saponins (Mylona et al., 2008), plants store these toxins as glycosides, sometimes in particular compartments, away from lytic enzymes that liberate the active toxins in response to the tissue damage that frequently accompanies herbivore and pathogen attack. Similarly, glucosinolates are compartmentalized away from the myrosinases that rapidly hydrolyze them to toxic isothiocyanates and other biologically active products (Matile, 1980; Halkier and Gershenzon, 2006). Glycosylation of steroidal alkaloids in tomato (*Solanum lycopersicum*; Itkin et al., 2011), and the saponins, hederagenin in barrelclover (*Medicago truncatula*; Naoumkina et al., 2010) and avenacin A-1 in common oat (*Avena sativa*; Mylona et al., 2008) are other examples that point to a similar chemical sequestration role exerted by glycosylation by UDP-glycosyltransferases (UGTs; Paquette et al., 2003).

Diterpene glycosides (DTGs) are a diverse class of compounds whose members are often associated with phytotoxic activities (Macias et al., 2008) and have potent anti-herbivore resistance/deterrence effects. For example, the abundance of monomers and dimers of capsianosides is correlated with thrips resistance in pepper (*Capsicum* spp.; Macel et al., 2019), and 17-hydroxygeranyllinalool diterpene glycosides (HGL-DTGs) in *Nicotiana* species have been shown to function in resistance against larvae of the specialist herbivore tobacco hornworm (*Manduca sexta*; Lou and Baldwin, 2003; Jassbi et al., 2008; Heiling et al., 2010) and the generalist herbivore tobacco budworm (*Heliothis virescens*; Snook et al., 1997).

**Figure 1:**
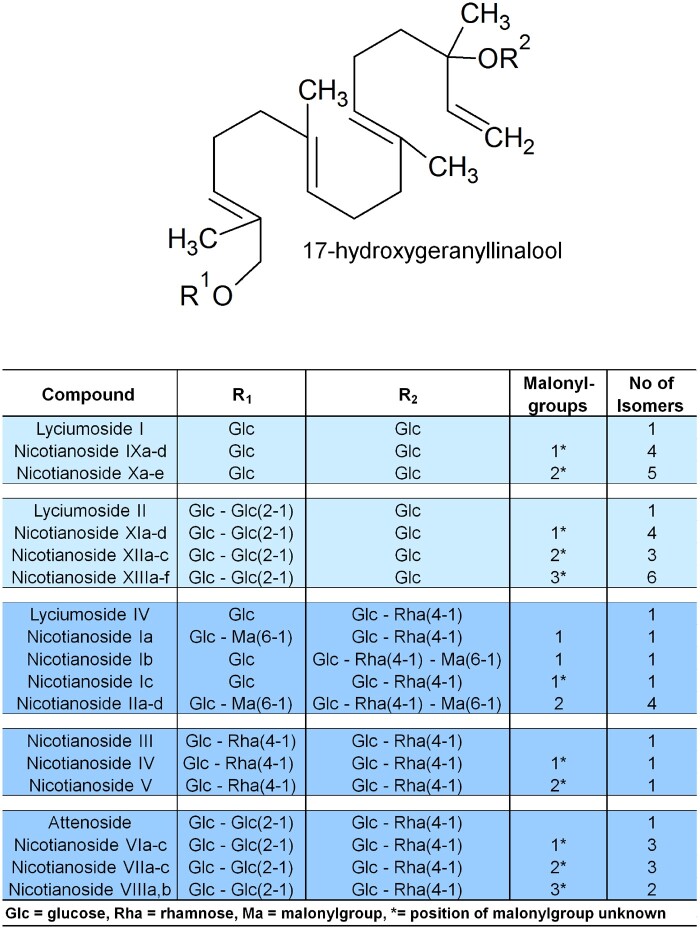
HGL-DTG biosynthetic pathway. Components of the diverse HGL-DTGs structures previously identified and annotated in the leaves of *N. attenuata* that differ with respect to their sugar and malonyl group compositions.

HGL-DTGs occur in the aboveground tissues of the native diploid tobacco *Nicotiana attenuata* (Heiling et al., 2010) as well as other *Nicotiana* species (Shinozaki et al., 1996; Snook et al., 1997; Lou and Baldwin, 2003; Jassbi et al., 2006; Heiling et al., 2010; Jassbi et al., 2010; Poreddy et al., 2015; Heiling et al., 2016), several other solanaceous genera including *Capsicum* (Izumitani et al., 1990; Hashimoto et al., 1997; Iorizzi et al., 2001; Lee et al., 2006, 2007, 2008; Lee et al., 2009) and *Lycium* (Terauchi et al., 1995, 1997a, 1997b, 1998a, 1998b; Roda et al., 2003), and the Asteraceae *Blumea lacera* (Akter et al., 2016). HGL-DTGs consist of an acyclic 17-hydroxygeranyllinalool (17-HGL) aglycone, which is conjugated at its C-3 and C-17 hydroxyl groups to glucose. These glucoses can be further glycosylated with different sugar moieties, such as additional glucose and rhamnose, at the C′-2, C′-4 or C′-6 hydroxyl groups, and malonylated at the C′-6 hydroxyl group of the glucose(s) (Heiling et al., 2010, 2016; Jassbi et al., 2010). To date, 45 HGL-DTGs, which differ in their sugar or malonyl decorations, have been putatively annotated or identified in *N. attenuata* (Figure 1; Heiling et al., 2016). However, it is unclear which of the many different HGL-DTGs or which structural components of HGL-DTGs are responsible for the observed deterrent (Jassbi et al., 2006) and resistance effects of these compounds against different herbivores (Snook et al., 1997). For example, the geranyllinalool precursor is known to be insecticidal in pine wood, and the same compound can be found in the defensive secretions of the termite *Reticulitermes lucifugus* (Baker et al., 1982; Lemaire et al., 1990). Jassbi et al. (2010) suggested that the 17-HGL aglycone is responsible for the feeding-deterrent characteristics of HGL-DTGs, but this hypothesis has not been tested.

While the chemical identity and anti-herbivore effects of HGL-DTGs have been the focus of major investigations, less is known about the possible phytotoxic effects of HGL-DTGs and how plants produces these compounds cope with the phytotoxic effects. This is, in part, because we know very little about the enzymes required for the biosynthesis of these metabolites. The 17-HGL aglycone is derived from the condensation of three five-carbon units of isopentenyl-pyrophosphate and dimethylallyl-pyrophosphate to produce the diterpenoid precursor geranylgeranyl-pyrophosphate (GGPP; Ohnuma et al., 1998; Dewick, 2002). This reaction is catalyzed by a plastidial geranylgeranyl pyrophosphate synthase (GGPPS; Jassbi et al., 2008; Heiling et al., 2010). The formation of GGPP is followed by its allylic rearrangement by a geranyllinalool synthase (GLS; Falara et al., 2014) that produces the tertiary alcohol geranyllinalool. However, the enzymes necessary for further hydroxylation and glycosylation steps, which contribute to much of the structural diversity of HGL-DTGs, remain to be characterized. Malonylation, a common decoration of plant specialized metabolites and the last biosynthetic step for HGL-DTGs, is mediated by the malonyltransferase NaMAT1 in *N. attenuata* (Li et al., 2018). However, all malonyl moieties of HGL-DTGs are rapidly lost when leaves are ingested by *Manduca sexta* larvae, suggesting that the malonylation of HGL-DTGs does not play a central role in herbivore defense (Poreddy et al., 2015). Interestingly, disruption of the uniform malonylation patterns of HGL-DTGs by silencing the expression of *NaMaT1* leads to a specific reduction in the floral style lengths of *N. attenuata* flowers (Li et al., 2018). This shows that specific decorations of a plant’s specialized metabolites can play a crucial, but poorly understood role in plant development. Malonylation of specialized metabolites, such as flavonoids or phenolic glucosides, is a common phenomenon that can enhance water solubility (Heller and Forkman, 1994) or facilitate the sequestration of compounds to molecular compartments, such as vacuoles (Taguchi et al., 2010). Whether other biosynthetic steps in the HGL-DTG pathway are similarly influential for plant development remains unclear.

In the current study, to evaluate the defensive value of glycosylation and the potential (auto)toxicity of the 17-HGL aglycone for both plant cells and insect herbivores, we identified the UGTs responsible for the glycosylation of HGL-DTGs by co-expression analysis of the 107 predicted UGTs in *N. attenuata* with two bait genes previously characterized for their involvement in the biosynthesis of 17-HGL-DTGs. RNAi silencing of these UGTs and enzyme assays with recombinant proteins confirmed that UGT74P3 and UGT74P4 are responsible for glucosylation of the C-3 and C-17 hydroxyl-groups of the aglycone, while UGT91T1 is the rhamnosyltransferase in the HGL-DTG pathway. As summarized in one of our previous studies (Heiling et al., 2016), all rhamnosylated HGL-DTG identified to date possess rhamnosyl moieties attached to glucose moieties, suggesting that rhamnosylation requires prior glucosylation. Finally, the genetic manipulations conducted in the present study revealed that glycosylation of the 17-HGL aglycone by UGT74P3 is crucial in preventing toxic effects to the plant and contributes to the metabolites’ defensive function during attack by *M. sexta* larvae.

## Results

### Identification of UGTs that might be responsible for HGL-DTG biosynthesis

To identify UGTs, we performed a genome-wide survey of *N. attenuata* and detected 107 putative UGT sequences containing the PSPG motif at the C-terminus (Supplemental Figure 1). We phylogenetically characterized these UGTs (Supplemental Figure 2 and Table 1) and examined their amino acid sequences (Supplemental Figure 3, Supplemental Table 2 and 3 and Supplemental Data Set 1).

HGL-DTGs are specialized metabolites of *N. attenuata*, whose biosynthetic steps of glycosylation are regulated by the defense phytohormone jasmonic acid (Heiling et al., 2010). Genes involved in a shared biological process tend to be co-expressed in large-scale expression datasets because they are frequently under the control of a common regulatory network (Saito et al., 2008). Following this rationale, we identified UGT candidates putatively responsible for the biosynthesis of HGL-DTGs by exploring gene co-expression in tissue-specific (local and systemic leaves as well as root tissue) transcriptomes analyzed at 1, 5, 9, 13, 17, and 21 h after simulated herbivory treatment as described in the Supplemental Methods. For the resulting compendium consisting of 150 microarray expression profiles, we calculated the Pearson Correlation Coefficients (PCCs) for 150 microarrays of the expressed UGTs with previously identified genes of the HGL-DTG biosynthetic pathway, *NaGGPPS* and *NaGLS*, (Supplemental Table 4). The PCC value between *GLS* and *GGPPS* transcript levels was 0.799 (Supplemental Figure 4). The transcript levels of three UGTs were significantly correlated with *GGPPS* (*UGT91T1* – PCC = 0.823, *UGT74P3* – PCC = 0.608, and *UGT74P5* – PCC = 0.684) and *GLS* (*UGT91T1* – PCC = 0.899, *UGT74P3* – PCC = 0.872, and *UGT74P5* – PCC = 0.868) and increased in response to wounding and simulated herbivory. Furthermore, we compared the expression levels between shoot and root tissues of the above genes. Consistent with the absence of HGL-DTG accumulation in *N. attenuata* roots, transcript accumulation was 50-fold and 3225-fold lower in roots compared to leaves for *GGPPS* and *GLS*, respectively. The three candidate UGTs showed a similar profile, with 2190-, 127-, and 20-fold lower transcript levels of *UGT91T1*, *UGT74P3*, and *UGT74P5*, respectively, in roots. Focusing on the 5-h time point collected from systemic leaves, we determined that *GGPPS* (increased six-fold), *GLS* (increased eight-fold), *UGT91T1* (increased seven-fold), *UGT74P3* (increased eight-fold), and *UGT74P5* (increased eight-fold) were all highly upregulated, relative to untreated control and mechanical wounding conditions, in response to simulated herbivory treatment (Supplemental Figure 4 and 5; Supplemental Data Set 2).

A phylogenetic analysis including functionally characterized UGTs (Supplemental Figure 6) showed a close relationship of UGT74P3 and UGT74P5 protein sequences with two previously characterized diterpene glucosyltransferases [SrUGT74G1–steviol glycoside glucosyltransferase from sugarleaf (*Stevia rebaudiana*) and CsGIT2–crocetin glucosyltransferase from saffron crocus (*Crocus sativus*; Cote et al., 2000)]. UGT91T1 showed a close phylogenetic relationship to three of the very few functionally characterized flavonoid rhamnosyltransferases [CmF7G12RT–flavonoid-1, 2-rhamnosyltransferase from pomelo (*Citrus maxima*), GmF3G6R–flavonol-3-O-glucoside-α-1, 6-*L*-rhamnosyltransferase from soybean (*Glycine max*), and PhA3ART–anthocyanidin-3-O-glucoside-α-1, 6-*L*-rhamnosyltransferase from *Petunia x hybrida*].

Based on co-expression with known genes of the HGL-DTG pathway, their high expression levels in leaf tissues, and their phylogenetic relationships, we selected three candidate UGTs, *UGT91T1*, *UGT74P3*, and *UGT74P5*, for further characterization.

### Virus-induced gene silencing (VIGS) reveals the roles of three UGTs in HGL-DTG production

Using a well-established transient VIGS approach (Saedler and Baldwin, 2004), we first examined the consequences of independently silencing the three candidate UGTs for HGL-DTG production. Seventeen days after inoculation with *Agrobacterium tumefaciens* harboring the appropriate constructs, transcript abundances in the leaves of respective plants were reduced by 98.5% for *UGT91T1* in pTVUGT91T1, 85% for *UGT74P3* in pTVUGT74P3, and 94.3% for *UGT74P5* in pTVUGT74P5 relative to the empty vector (EV) controls (pTV00; Supplemental Figure 7). Co-silencing resulted in reduced transcript abundances of *UGT74P3* and *UGT74P5* in both pTVUGT74P3 and pTVUGT74P5 plants (Supplemental Figure 8).

To test the hypothesis that UGT91T1, UGT74P3, and UGT74P5 control the glycosylation steps in the HGL-DTG pathway, we analyzed the leaf metabolome by UPLC/TOF-MS of the different VIGS plants under control condition (lanolin, Lan) and after treatment with a lanolin paste containing methyl jasmonate (Lan + MeJA), which is known to strongly induce the de novo production of HGL-DTGs (Heiling et al., 2010). We putatively identified and annotated HGL-DTGs using a previously established rapid de-replication and identification workflow, which is based on high resolution MS data analysis and a library of putative and identified HGL-DTGs (Heiling et al., 2016).

**Figure 2:**
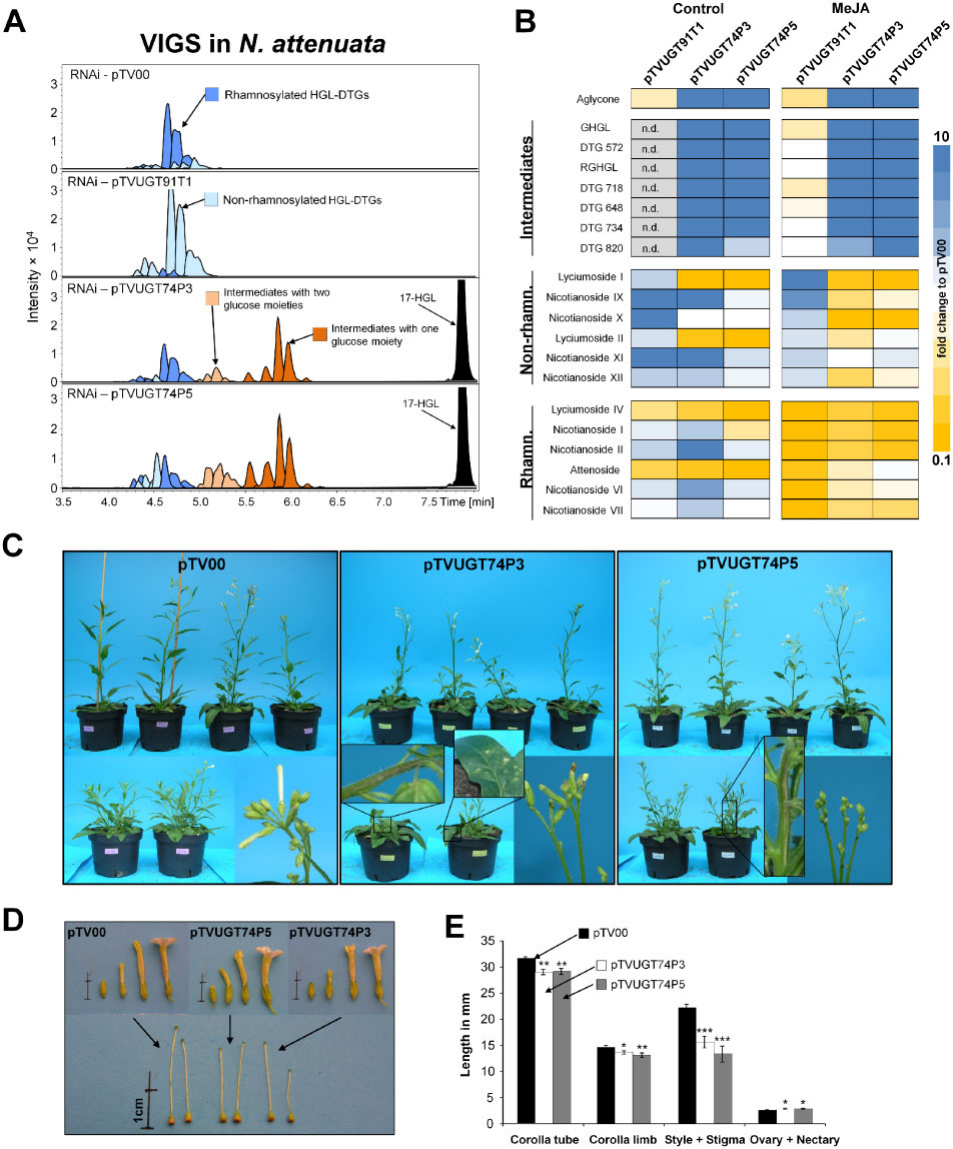
Metabolite profiling and morphologies of *N. attenuata* plants transiently silenced in the expression of HGL-DTG-predicted UGTs by VIGS. A, EIC for identified HGL-DTGs in leaves of 37-day-old elongated *N. attenuata* plants silenced in *UGT91T1*, *UGT74P3*, or *UGT74P5* transcript accumulation as well as in the EV controls (pTV00). HGL-DTGs were categorized into rhamnosylated, non-rhamnosylated, and intermediates with one or two glucose moieties to facilitate visualization. The 17-hydroxygeranyllinallool (17-HGL) aglycone was only detected in transiently silenced pTVUGT74P3 and pTVUGT74P5 lines. B, Heatmap visualization of the patterns of deregulation in control plants or in plants treated with 150 µg methyl jasmonate (MeJA) in 20 µL lanolin paste (*N* = 5). The color gradient visualizes fold changes in individual HGL-DTGs for each of the VIGs constructs compared to the average in the pTV00 EV VIGS plants. C, Morphological alterations observed in pTV00, pTVUGT74P3, and pTVUGT74P5 transiently transformed plants ranged from necrotic spots and tissues to necrotic apical meristem and flower buds frequently stalled in the opening process. Additional phenotypic details are provided in Supplemental Figure 7, a–c. D and E, Morphological alterations of the corolla tube, corolla limb, style, ovary and nectary (average ± se; *N* = 20). Asterisks indicate significant differences between the EV control and pTVUGT74P3 or pTVUGT74P5 VIGS plants (*P ≤ 0.05, ** *P* < 0.01, *** *P* < 0.001).

The levels of rhamnosylated HGL-DTGs, most particularly of lyciumoside IV and attenoside, were reduced in UGT-silenced plants compared to pTV00 EV controls. The levels of non-rhamnosylated HGL-DTGs lyciumoside I and lyciumoside II increased in the HGL-DTG chemotype of pTVUGT91T1 VIGS plants (Figure 2A;Supplemental Data Set 3). This shift was more pronounced after the Lan + MeJA treatment. In pTVUGT91T1 plants, most rhamnosylated HGL-DTGs were barely detectable and non-rhamnosylated HGL-DTG levels were strongly increased compared to the pTV00 controls (Figure 2, A and B). Furthermore, we detected the 17-HGL aglycone as well as several novel compounds in pTVUGT74P3 and pTVUGT74P5 VIGS plant profiles (Figure 2A). We defined this class of compounds as intermediate HGL-DTGs, and our MS annotation workflow suggested they were malonylated and non-malonylated compounds with only one or two sugar moieties at either the C-3 or C-17 hydroxy-group of the aglycone.

**Figure 3:**
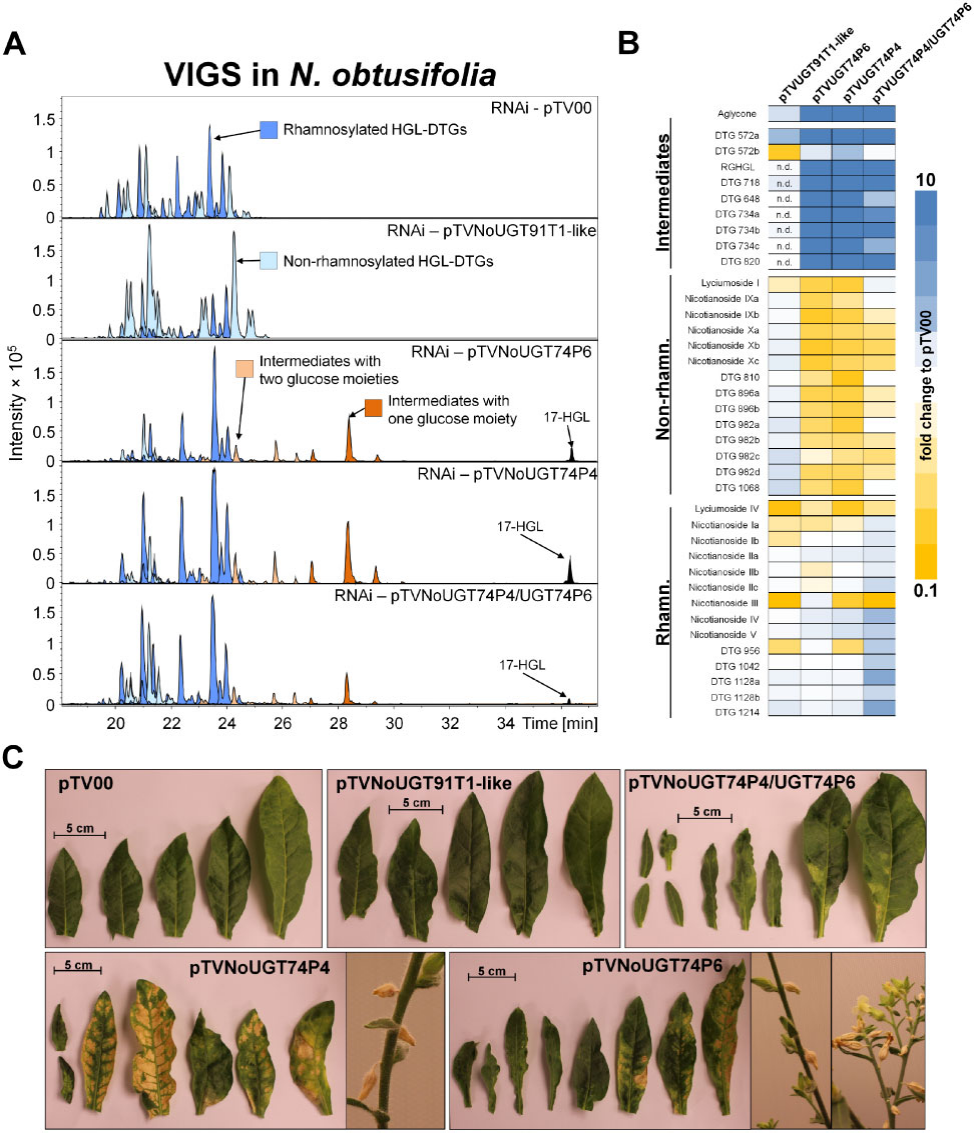
Metabolite profiling and morphologies of *N. obtusifolia* plants transiently silenced in the expression of HGL-DTG-predicted UGTs by VIGS. A, EIC for the identified HGL-DTGs of 37-day-old elongated *N. obtusifolia* plants silenced in No*UGT91T1*-like, No*UGT74P4* and No*UGt74P6* transcript accumulation as well as in the EV (pTV00) controls. Rhamnosylated, non-rhamnosylated, and intermediate HGL-DTGs with one or two glucose moieties were color-categorized as in Figure 3. The 17-HGL aglycone was only detected in pTVNoUGT74P4, pTVNoUGT74P6, and the double construct pTVNoUGT74P4/UGT74P6 VIGS plants. B, Heatmap visualization of deregulations in the leaf HGL-DTG profiles of transiently transformed pTVNoUGT91T1-like, pTVNoUGT74P4, pTVNoUGT74P6, or pTVNoUGT74P4/UGT74P6 plants (*N* = 3–7). The color gradient visualizes fold changes in individual HGL-DTGs for each of the VIGS constructs compared to the average in the pTV00 EV VIGS plants. C, Morphological alterations in pTV00, pTVNoUGT91T1-like, pTVNoUGT74P4, pTVNoUGT74P6, or pTVNoUGT74P4/UGT74P6 ranged from necrotic spots to a high percentage of stalled flower buds. Additional phenotypic details are reported in Supplemental Figure 9.

We searched for orthologs to these three UGTs in Nicotiana *obtusifolia*, a sympatric perennial tobacco species to *N. attenuata.* We identified No*UGT74P4* (95% homology to *UGT74P3*), No*UGT74P6* (93% homology to *UGT74P5*), and NoUGT91T1-like (92% homology to *UGT91T1*). Using VIGS, we inoculated *A. tumefaciens* harboring the appropriate constructs into 25-day-old *N. obtusifolia* plants and detected 91.2% transcript reductions of No*UGT91T1-like* in pTVNoUGT91T-like, 98.5% of No*UGT74P3* in pTVNoUGT74P3, and 93.1% of *NoUGT74P6* in pTVNoUGT74P6 at 14 days after inoculation (Supplemental Figure 9). pTVNoUGT91T1-like plants showed an overall decrease in rhamnosylated HGL-DTG levels compared to the pTV00 controls and increased levels of non-rhamnosylated compounds (Figure 3, A and B; Supplemental Data Set 4). In pTVNoUGT74P4 and pTVNoUGT74P6 plants, the abundance of intermediate HGL-DTGs as well as the 17-HGL aglycone increased and non-rhamnosylated HGL-DTGs decreased compared to the pTV00 controls. Silencing both glucosyltransferases in *N. obtusifolia* by means of a double construct resulted in the same phenotypic alterations, namely the appearance of the intermediate HGL-DTGs and the 17-HGL aglycone. Ten novel intermediate HGL-DTGs were putatively identified based on the de-replication workflow for HGL-DTGs. A detailed MS analysis and putative structural description of *N. attenuata* and *N. obtusifolia* HGL-DTGs can be found in Supplemental Data Set 5 and 6 and Supplemental Figures 10, a and b and 11, a to c.

### 17-HGL glucosylation activities of UGT74P3/P4 and UGT74P5 proteins

**Figure 4:**
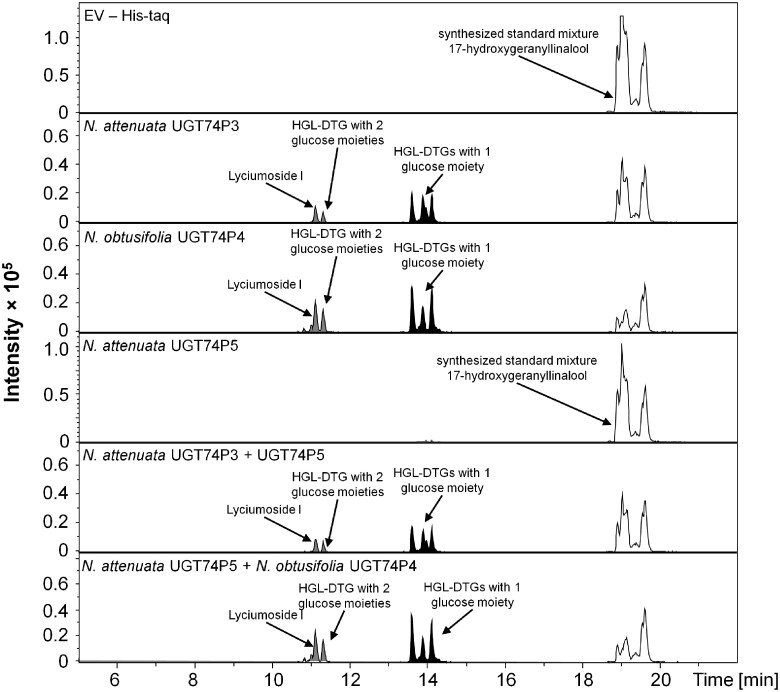
Recombinant UGT74P3, UGT74P4, and UGT74P5 proteins glucosylate the 17-HGL aglycone. Enzyme activity assays of the recombinant UGT74P3, UGT74P4, and UGT74P5 proteins expressed in *E. coli* BL21 DE3 cells. Chromatograms (EIC traces for aglycone *m*/*z* 329.2475, HGL-DTGs with one glucose moiety *m*/*z* 491.3003 and HGL-DTGs with two glucose moieties *m*/*z* 653.3507) of analyses of four UGT recombinant proteins incubated for 3 h in 50 mM Tris HCL pH 7.0 with 5 mM 17-HGL in the presence of 5 mM UDP-glucose. Additionally, activity assays combining UGT74P5 with UGT74P3 or UGT74P4 were performed, but the results did not differ from the results of the enzyme assays using only UGT74P3 or UGT74P4.

The co-silencing of both *UGT74P3* and *UGT74P5* resulted in strong overlap in the metabolic alterations of the HGL-DTG profiles produced by pTVUGT74P3 and pTVUGT74P5 plants and suggested that at least one of these enzymes might play a concerted role in the formation of lyciumoside I and lyciumoside II through glucosylation of the C-3 or C-17 hydroxyl-group of 17-HGL. HGL-DTGs typically contain one D-Glc moiety at the C-3 and another at the C-17 hydroxyl group. Lyciumoside II derives from lyciumoside I by the attachment of a second D-Glc at the C′-2 position of the C-17 D-Glc. To test this hypothesis, we analyzed the in vitro activity of the UGT enzymes when recombinantly produced in *Escherichia coli*. UDP-glucose was used as sugar donor with a mixture of all six isomers of synthesized 17-HGL (Supplemental Figure 12) as substrates. NaUGT74P3 and its *N. obtusifolia* homologue NoUGT74P4 readily used UDP-glucose as a donor, producing several novel products with a mass-to-charge ratio of *m/z* 491.3003 [M+Na]^+^ corresponding to a mono-glucosylated 17-HGL and two additional products with *m/z* 653.3509 [M+Na]^+^ corresponding to lyciumoside I and another di-glucosylated 17-HGL (Figure 4). Lyciumoside I was identified by comparing retention times and MS/MS fragmentation patterns to a purified authentic standard, and the novel compounds were annotated based on comparisons of their MS/MS fragmentation patterns to an in-house database of HGL-DTG spectra. Thus, NaUGT74P3 and NoUGT74P4 function as UDP-glucosyltransferases for HGL-DTGs synthesis via their ability to catalyze glucosylation at C-3 and C-17 of the 17-HGL aglycone. Enzymatic assays conducted with NaUGT74P5 and NoUGT74P6 in the presence UDP-glucose as donor and 17-HGL as substrate also produced a mass signal at *m/z* 491.3003 [M+Na]^+^, which may correspond to a mono-glucosylated 17-HGL. Due to the extremely low intensity of this mass signal, no MS/MS spectrum could be collected to confirm this annotation. Furthermore, the combination of NaUGT74P5 with NaUGT74P3 or NoUGT74P4 did not result in any detectable products.

### VIGS of UGTs causes morphological defects and necrosis

In addition to the mild TRV infection symptoms such as curly leaves and local chlorosis that were also seen in EV (pTV00) and pTVUGT91T1 plants (Supplemental Figure 13a), we noticed that pTVUGT74P3 and pTVUGT74P5 plants displayed severe morphological alterations that ranged from the presence of necrotic spots to necrotic apical meristems and a high percentage of stalled flower buds (Figure 2C;Supplemental Figure *13*, *b and c*). Furthermore, we isolated organs of mature flowers from pTVUGT74P3 and pTVUGT74P5 VIGS plants collected at anthesis (Figure 2D) and observed significant reductions in the lengths of styles and corolla tubes and in the width of the corolla limb, as well as an increased combined lengths of ovary + nectary, compared to the pTV00 controls (Figure 2E).

Nicotiana *obtusifolia* plants showed the same symptoms. Plants transiently silenced with pTVNoUGT91T1-like did not display any additional phenotypic alterations compared with the pTV00 control plants. However, *N. obtusifolia* plants silenced in *UGT74P4* and *UGT74P6* expression showed similar morphological modifications to those observed in *N. attenuata*, ranging from necrotic spots, misshapen, and deformed leaves, to stalled flower buds (Figure 3C;Supplemental Figure 14), with the exception of apical meristem necrosis. Floral morphology was not examined in *N. obtusifolia*.

Finally, we generated a VIGS construct (pTVGLS) targeting Na*GLS*. The encoded protein is predicted to be part of the HGL-DTG biosynthetic pathway that catalyzes the conversion of GGPP to geranyllinalool (Falara et al., 2014). Fourteen days after inoculation with pTVGLS, we detected a 97.5% reduction in *GLS* transcript abundance compared to the pTV00 controls, which was accompanied by a strong reduction in almost all HGL-DTG types (Supplemental Figure 15 and Supplemental Data Set 7). Consistent with the expected disruption of the biosynthesis of geranyllinalool, no novel intermediate HGL-DTGs were detected. Beyond the typical TRV infection symptoms, no morphological alterations were detected, suggesting that the phenotype of the glycosyltransferase-impaired lines resulted from the accumulation of intermediate HGL-DTGs or the aglycone 17-HGL.

### Plants stably silenced for *UGT74P3* and *UGT74P5* display severe developmental defects

**Figure 5:**
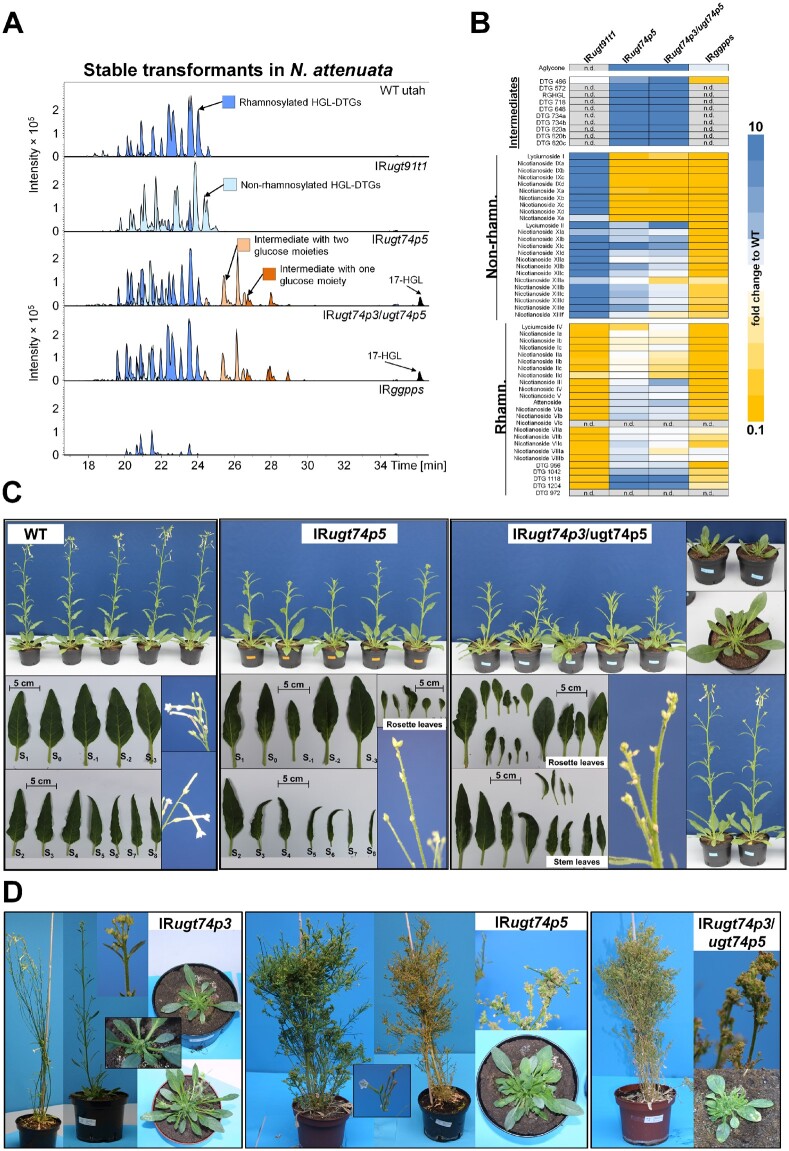
Metabolite profiling and morphological characterization of stably silenced *N. attenuata* plants. A, EICs for identified HGL-DTGs in leaves of 42-day-old elongated *N. attenuata* plants silenced in *GGPPS*, *UGT91T1*, *UGT74P3*, and *UGT74P5* transcript accumulation as well as WT control plants. HGL-DTGs were categorized into rhamnosylated, non-rhamnosylated HGL-DTG, and intermediates with one or two glucose moieties to facilitate visualization. The 17-HGL aglycone was only detected in IR*ugt74p5* and IR*ugt74p3/ugt74p5* plants. B, Heatmap visualization of deregulations in the leaf HGL-DTG profile of IR*ugt91t1*, IR*ugt74p5*, IR*ugt74p3/ugt74p5*, and IR*ggpps* (*N* = 5). The color gradient visualizes fold changes in individual HGL-DTGs for each of the stably transformed lines compared to the average in the WT plants. C, Morphological alterations in IR*ugt74p5* and IR*ugt74p3/ugt74p5* with milder phenotypes ranged from necrotic spots and tissues, altered leaf shape and thickness, to apical meristem necrosis and a high percentage of stalled flower buds and overall highly stunted growth. Additional details of these phenotypes are shown in Supplemental Figure 14, a–c. D, 1-year-old independent T0-transformants silenced in the expression of *UGT74P3*, *UGT74P5*, and *UGT74P3/UGT74P5*. Strong morphological alterations ranging from stunted growth, succulent leaves, stalled flower buds to a “broom” like appearance were consistently detected among T0-transformants. Viable seeds were produced by a few transformants.

To further elucidate the roles of UGT74P3 and UGT74P5 in controlling the flux of HGL-DTG synthesis in *N. attenuata* as well as the mechanisms responsible for the developmental defects detected during transient gene silencing, we generated 36 independent stably transformed transgenic *N. attenuata* plants harboring *UGT74P3* and *UGT74P5* inverted-repeat (IR) silencing constructs in their genomes. Similar to the VIGS experiments, IR*ugt74p3* and IR*ugt74p5*-silenced plants displayed strong effects on growth and development. Almost all stable T_0_-transformants developed either a dwarfish growth or a “broom-like” appearance (Figure 5D). Compared to wild-type (WT) plants, many of the transformed plants were strongly retarded in their growth, with thicker woody or possibly suberized side branches and deformed or thick, succulent-like leaves. Most flower buds of these transformed plants were small and aborted before fertilization. A few T_0_-transformants of both constructs exhibited a milder phenotype and produced mature flowers and seed capsules. The phenotypic characteristics of IR*ugt74p3* transformants could not be transferred to the T_2_ generation, as T_1_ plants aborted most flower buds early during development and did not produce fertile flowers. IR*ugt74p5* transformants could be propagated and two lines from the same T_1_ parental plant were established (Supplemental Figure 16). Similar to the T_0_ transformants, both IR*ugt74p5* lines displayed a milder phenotype compared to IR*ugt74p3*, but still with severe morphological alterations ranging from necrotic spots and necrotic apical meristem to small deformed or thicker succulent leaves (Supplemental Figure 17, a to c) and numerous stalled flower buds (Figure 5C). IR*ugt74p5* plants were also smaller compared to WT (Supplemental Figure 18). Additionally, we established a heterozygous double construct *UGT74P3/UGT74P5* for which ∼75% of the silenced plants exhibited similar morphological alterations. Similar to the IR*ugt74p3* plants, the homozygous double construct was lethal and did not produce seeds.

In contrast to the IR*ugt74p3* and IR*ugt74p5*-silenced plants, two independent stably silenced *UGT91T1* lines did not show any morphological alterations compared to WT (Supplemental Figure 19).

**Figure 6:**
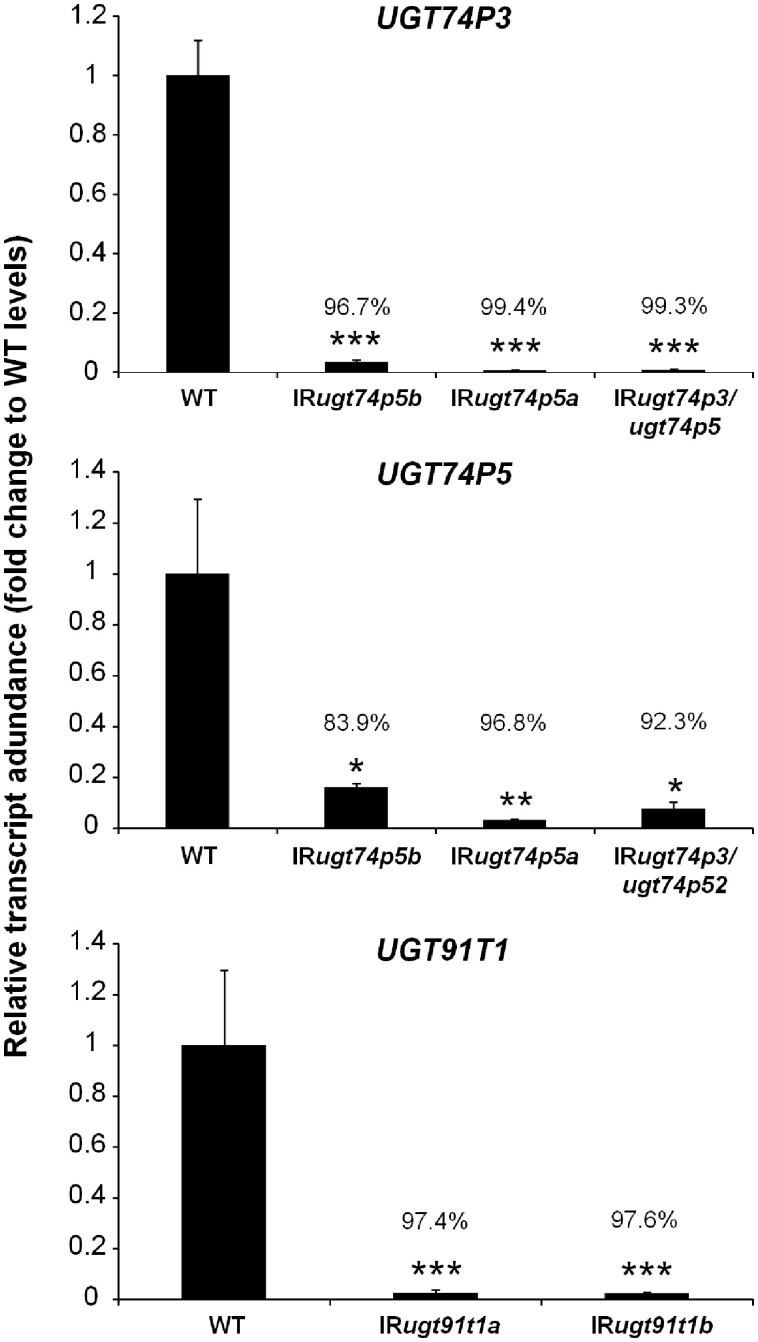
Silencing efficiency for the three 17-HGL-DTG biosynthetic UGTs in IR*ugt91t1*, IR*ugt74p5*, IR*ugt74p3/ugt74p5* plants. Relative transcript abundance of *UGT91T1*, *UGT74P3*, and *UGT74P5* in leaves of stably transformed *N. attenuata* plants (average ± se; *N* = 4). Asterisks indicate significant differences between the WT control and stable transformants (*P ≤ 0.05, ** *P* < 0.01, *** *P* < 0.001).

In addition to the careful examination of shoot morphological alterations (roots were not examined), we analyzed the transcript abundances of *UGT74P3*, *UGT74P5*, and *UGT91T1* in leaves of 52-day-old plants of the different transgenic lines (Figure 6). The silencing efficiency for *UGT91T1* transcript abundance was 97.4% in IR*ugt91t1a* and 97.6% in IR*ugt91t1b*. *UGT74P5* transcript accumulation was strongly repressed in all IR*ugt74p5* lines tested (83.9% in IR*ugt74p5b*, 96.8% in IR*ugt74p5a* and 92.3% in IR*ugt74p3/ugt74p5)*. The silencing efficiency for *UGT74P3* in IR*ugt74p3/ugt74p5* was 99.3%, but we also detected a strong co-silencing of *UGT74P3* expression in IR*ugt74p5b* (96.7%) and IR*ugt74p5a* (99.4%).

### Metabolic profiling confirms the unique HGL-DTG profiles of stably silenced UGT lines

From UPLC/qTOF-MS measurements and the application of our de-replication workflow, we identified 60 HGL-DTGs in the leaf extracts of the different stably silenced lines. The HGL-DTGs were annotated and classified into three categories based on their chemical compositions: rhamnosylated, nonrhamnosylated, and biosynthetic intermediates only detectable upon disruption of the glucosylation steps (Figure 5). Both stable lines impaired in *UGT74P5* expression showed the highest overall HGL-DTG levels (Supplemental Figure 20). Nonrhamnosylated lyciumoside I and its malonylated forms, nicotianoside IXa-d and Xa-e, were barely detectable, and the level of rhamnosylated lyciumoside IV was strongly reduced. While rhamnosylated nicotianoside I and II levels remained constant compared to WT plants, both transgenic IR*ugt74p5* lines exhibited elevated levels of lyciumoside II and attenoside and their malonylated forms, nicotianoside XIa-d, XIIa-c, XIIIa-f, and nicotianoside VIa-c, VIIa-c and VIIIa-b, respectively. More complex reconfigurations were also detected. For example, nicotianoside III levels remained unchanged in transgenic IR*ugt74p5* lines, but the levels of its malonylated forms, nicotianoside IV and V, increased. Furthermore, we putatively identified 10 novel highly abundant intermediate HGL-DTGs and the 17-HGL aglycone, which were not detected in WT plants. Annotation of the MS/MS spectra of these novel HGL-DTGs indicated that they were lower molecular weight HGL-DTG biosynthetic intermediates with either one (G-3-HGL, G-17-HGL) or two (RGHGL and DTG 648) sugar moieties attached to the 17-HGL (Figure 5; Supplemental Figure 11, a–c and Supplemental Data Set 6). Additionally, malonylated forms of these compounds were also detected (DTG 572, DTG 718, DTG 734, and DTG 820; Figure 5, A and B; Supplemental Figure 11, a–c and Supplemental Data Set 6). The transgenic line IR*ugt74p5b* was used for all further experiments. Plants harboring the heterozygous double construct IR*ugt74p3/ugt74p5* exhibited an almost identical pattern of accumulation of known and novel intermediate HGL-DTGs. The most prominent difference compared to the IR*ugt74p5* lines was the specific increases detected for lyciumoside II and lyciumoside IV.

In both stable lines impaired in *UGT91T1* expression, the levels of all rhamnosylated HGL-DTGs were strongly reduced. Reciprocally, the levels of all nonrhamnosylated HGL-DTGs were highly increased, leading to a complete shift between rhamnosylated and non-rhamnosylated compounds within the HGL-DTG profile (Figure 5, A and B). Neither intermediate HGL-DTGs nor the 17-HGL aglycone could be detected in either of the transgenic IR*ugt91t1* lines. IR*ugt91t1* line A was selected for all further experiments. Additionally, we analyzed the HGL-DTG profiles in IR*ggpps* plants (Figure 5). GGPPS is responsible for the synthesis of GGPP, the precursor for all HGL-DTGs (Jassbi et al., 2008). The levels of almost all HGL-DTGs were reduced, and no intermediates could be detected in these plants. Only the levels of nicotianoside VIIIa and VIIIb increased. A more detailed analysis of the HGL-DTG profiles of all stably silenced lines can be found in Supplemental Data Set 8.

In addition, we performed a detailed metabolite profiling of central carbon metabolism intermediates and specialized metabolites (Supplemental Methods). This quantitative profiling, which included 23 amino acids and biogenic amines, small organic acids, phenylpropanoids, and derivatives, sugars, and phytohormones such as gibberellins, cytokinins, and jasmonates (Supplemental Figures 21 and 22; Supplemental Data Sets 9 and 10), showed profound reconfigurations of central carbon and phytohormone metabolism in IR*ggpps* plants.

### Abolishing 17-HGL aglycone synthesis in IR*ggpps* prevents the strong morphological alterations that result from silencing *UGT74P3* and *UGT74P5*

**Figure 7:**
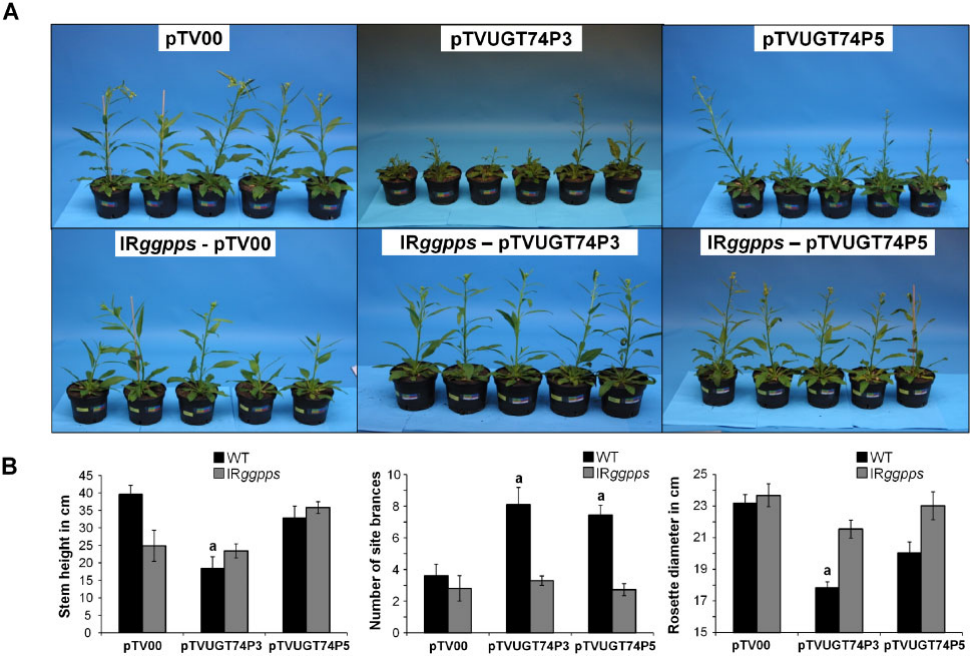
Abolishing 17-HGL aglycone synthesis by silencing Na*GGPPS* abrogates morphological alterations resulting from the silencing of *UGT74P3* and *UGT74P5.* A, Morphological alterations of 42-day-old *N. attenuata* plants stably transformed to silence Na*GGPPS* expression (IR*ggpps*) and WT after inoculation with *A. tumefaciens* harboring pTVUGT74P5, pTVUGT74P3, and the EV control (pTV00) VIGS constructs. B, Stem height, number of side branches and rosette diameter in WT and IR*ggpps* (average ± se; *N* = 10). Different letters indicate significant differences between EV control and transient silenced lines (*P ≤ 0.05).

To determine if the morphological alterations of plants transiently and stably silenced in *UGT74P3* and *UGT74P5* are mediated by altered HGL-DTG profiles, we performed a VIGS experiment involving the silencing of *UGT74P3* and *UGT74P5* in IR*ggpps* and WT plants. The quantification of 17-HGL concentrations in leaf tissues using a quantitative U(H)PLC-triple quadrupole-MS method (Supplemental Figure 23) showed accumulation of 17-HGL only in WT *N. attenuata* plants transformed with the pTVUGT74P3 or pTVUGT74P5 VIGS construct (Supplemental Figure 24). Seventeen days after inoculation, we analyzed the morphological alterations triggered by *UGT74P3* and *UGT74P5* silencing (Figure 7). WT plants transiently transformed with pTVUGT74P3 and pTVUGT74P5 exhibited the phenotypes described above, ranging from necrotic spots and necrotic apical meristems to stalled and aborted flower buds (Figure 7A;Supplemental Figures 25–27). The number of side branches increased and the rosette diameter decreased in these plants. IR*ggpps* plants transformed with pTVUGT74P3 and pTVUGT74P5 did not show any severe morphological alterations. Specifically, the number of side branches and rosette diameters of these plants were not different from those of WT plants (Figure 7B). This combination of genetic manipulations suggests that the ectopic accumulation of several intermediates and 17-HGL might be directly responsible for the observed morphological alterations.

### Excess of 17-HGL triggers cell necrosis in *N. attenuata* leaves

**Figure 8:**
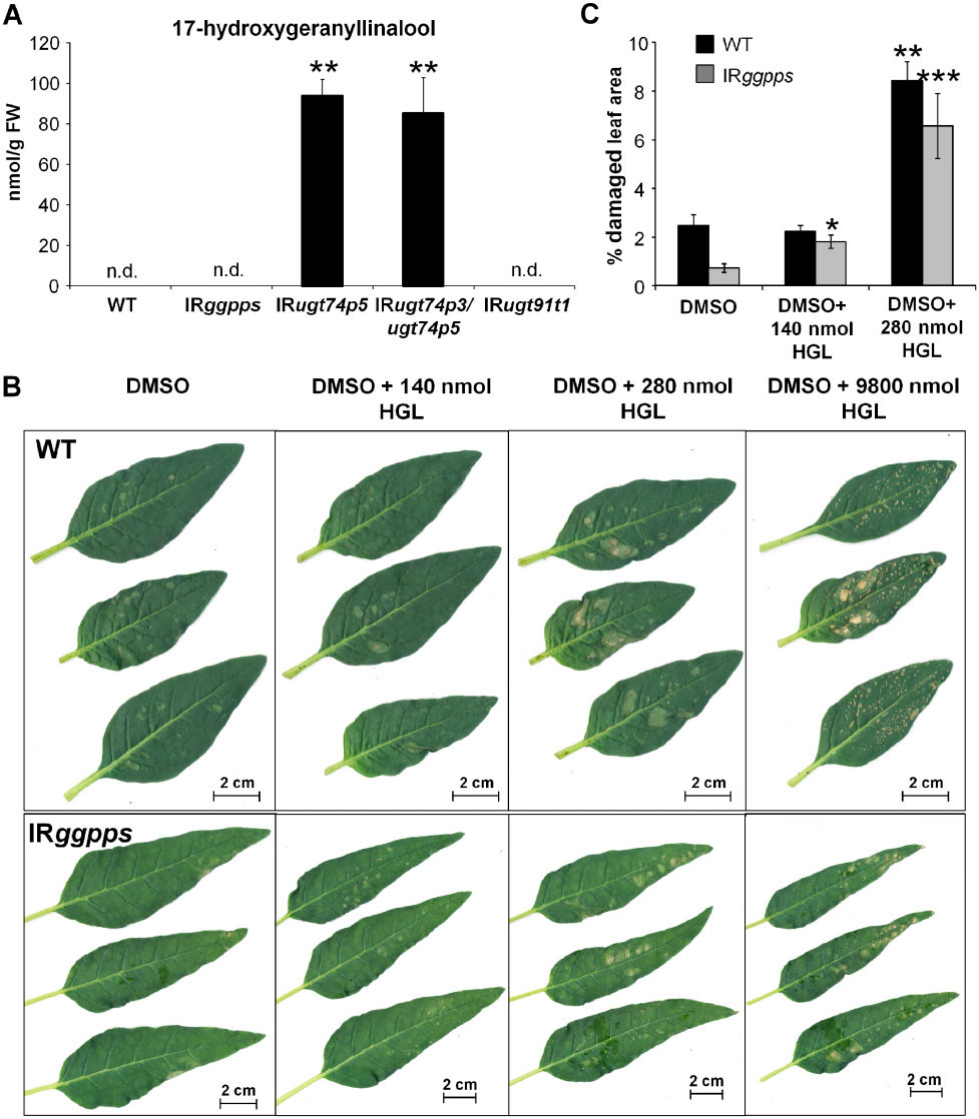
Application of 17-HGL aglycone results in necrotic lesions that phenocopy those observed in IR*ugt74p5* and IR*ugt74p3/ugt74p5* plants. A, Concentrations of HGL in leaf material of WT, IR*ggpps*, IR*ugt74p5*, IR*ugt74p3/ugt74p5*, and IR*udp91t1*. B, Necrotic leaf tissue of 32-day-old elongated WT and 48-day-old flowering IR*ggpps* plants treated with DMSO, DMSO + 140 nmol HGL, DMSO + 280 nmol HGL, and DMSO + 9800 nmol HGL after 1 day. C, Percentage of damaged leaf area in WT (*N* = 3) and IR*ggpps* (average ± se; *N* = 5). Asterisks indicate significant differences between Control (DMSO) and treated (+HGL) leaves (*P ≤ 0.05, ** *P* < 0.01, *** *P* < 0.001).

We determined the absolute concentration of 17-HGL in leaves of *N. attenuata* plants (*N* = 5) impaired in *UGT74P5* or *UGT74P3/UGT74P5* expression that exhibited the severe phenotype reported above. The concentration (mean ± sd) of 17-HGL was 94 ± 8 nmol g^−1^ FW in IR*ugt74p5* and 85.4 ± 17.6 nmol g^−1^ FW in IR*ugt74p4/ugt74p5* (Figure 8A). To determine the phytotoxic effect of 17-HGL, we used 32-day-old early elongated *N. attenuata* (*N* = 3) WT plants and 48-day-old flowering *N. attenuata* plants impaired in *GGPPS* expression (*N* = 5). The average leaf mass was estimated to be 1.3–1.6 g for the rosette leaves of WT and the stem leaves of transgenic IR*ggpps* plants. Three leaves of each plant were treated with either 20 µL of DMSO or DMSO with 140, 280, or 9800 nmol 17-HGL. Independent of the concentration of 17-HGL, DMSO application resulted in a mild dissolution of the epidermal surface. Analysis of the damaged leaf area 1 day after application revealed strong necrotic regions at all three 17-HGL concentrations (Figure 8B). Significant increases in leaf damage were detected starting at 140 nmol g^−1^ FW for IR*ggpps* plants and 280 nmol g^−1^ FW in WT plants (Figure 8C).

### *Manduca sexta* larvae perform poorly on transformed lines impaired in HGL-DTG glycosylation

HGL-DTGs are abundant, potent anti-herbivore defense metabolites in the aboveground tissues of *N. attenuata* (Heiling et al., 2010). Although 17-HGL was suggested to be a feeding deterrent (Jassbi et al., 2010), which of the many different HGL-DTGs or structural components of HGL-DTGs account for the observed deterrent (Jassbi et al., 2006) and resistance effects against herbivores (Snook et al., 1997) remains unclear. To elucidate the defensive value of glucosylated and rhamnosylated HGL-DTGs, we conducted performance assays with *M. sexta* larvae reared on leaf disks of transgenic *N. attenuata* plants impaired in rhamnosylation (IR*ugt91t1*) and glucosylation (IR*ugt74p5*, IR*ugt74p3/ugt74p5*) of HGL-DTGs. Additionally, we included IR*ggpps*, which has been shown to dramatically decrease HGL-DTG levels and enhance the growth of *M. sexta* (Heiling et al., 2010).

**Figure 9:**
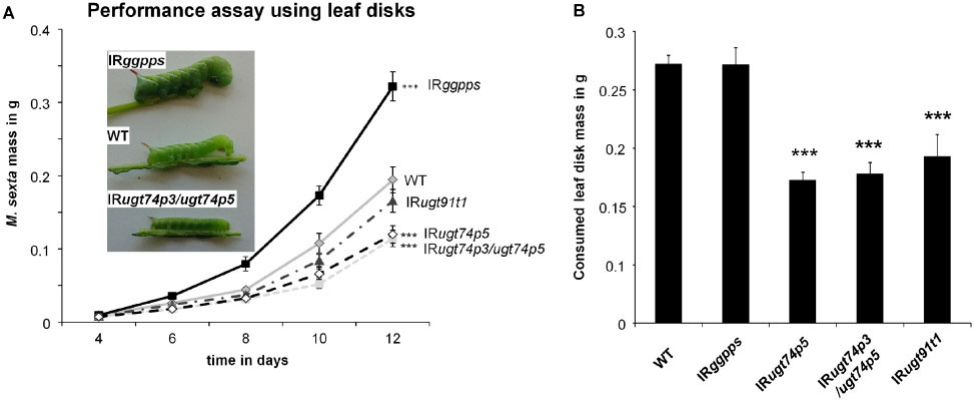
Performance assays show reduced growth of *M. sexta* fed on transformed lines impaired in HGL-DTG glycosylation. A, Mass of *M. sexta* larvae feeding on leaf disk material of four stably transformed plants impaired in glucosylation (IR*ugt74p5*, IR*ugt74p3/ugt74p5*) and rhamnosylation (IR*ugt91t1*) of HGL-DTGs as well as the formation of their precursor geranylgeranyl diphosphate (IR*ggpps*; average ± se; *n* = 24–30). Larvae grow significantly larger on IR*ggpps* (*P* = 0.005) and are significantly smaller on IR*ugt74p3/ugt74p5* (*P* = 0.005) and IR*ugt74p5* (*P* = 0.006) by day 6, as determined by Mann–Whitney–Wilcox pairwise tests. For clarity, significance is only shown for day 12: *P < 0.05, **P < 0.01, ***P < 0.001. B, Mass of leaf disks from transgenic plants consumed by caterpillars (average ± se; *n* = 26–30 leaf disks with one larva). Larvae fed IR*ugt74p5*, IR*ugt74p3/ugt74p5*, and IR*ugt91t1* consumed significantly less leaf disk material than larvae fed WT tissue, as determined by Mann-Whitney-Wilcox Pairwise Tests. *P < 0.05, **P < 0.01, ***P < 0.001.

Caterpillars feeding on IR*ggpps* plants showed enhanced growth after 6 days and gained 1.6-fold higher larval mass after 12 days of feeding (*P* < 0.001) compared to larvae feeding on WT leaf disks (Figure 9A). In contrast, *M. sexta* feeding on leaf disks from IR*ugt74p5* (∼0.6-fold, *P* = 0.028) and IR*ugt74p3/ugt74p5* (∼0.6-fold, *P* = 0.001) plants showed significantly reduced growth compared to larvae feeding on WT leaf disks. Caterpillars fed on leaf disks of IR*ugt91t1* displayed only slight reductions in growth (∼0.8-fold relative to WT).

Finally, we measured the mass of leaf tissue consumed by *M. sexta* larvae from leaf disks of IR*ggpps*, IR*ugt74p5*, IR*ugt74p3/ugt74p5*, and IR*ugt91t1* plants between days 8 and 10 and observed a strong decrease in consumption of IR*ugt74p5*, IR*ugt74p3/ugt74p5*, and IR*ugt91t1* leaf disks relative to WT (Figure 9B). A detailed statistical analysis can be found in Supplemental Data Set 11.

## Discussion

Plants have evolved highly diversified specialized metabolism pathways to resist both abiotic and biotic stresses as well as a series of mechanisms to mitigate cost/benefit trade-offs of specialized metabolite production and thereby maintain competitive ability. Innovations in specialized metabolism frequently result from the modification or direct recruitment of pre-existing scaffolds produced by core metabolic pathways. These scaffolds serve as substrates for modifying enzymes that add many different types of decorations (Gachon et al., 2005). In this respect, the large numbers of UGTs that populate plant genomes define a versatile glycosylation toolbox that has likely facilitated the functional diversification of specialized metabolism across plant lineages.

Here, we identified and phylogenetically characterized 107 UDP-glycosyltransferases of the superfamily 1 in *N. attenuata*. We used a co-expression approach relying on overall gene-to-gene correlations across all analyzed tissues and response to herbivory (Supplemental Figure 4) and identified three novel UGTs responsible for the synthesis of 17-HGL-DTGs in *N. attenuata*. UGT74P3 and UGT74P4 are GT-type enzymes that use UDP-glucose to attach the first glucose moieties to the C-3 and C-17 hydroxyl groups of 17-HGL. Additionally, we showed that the glucosylation of the 17-HGL aglycone is crucial to prevent an autotoxic effect of 17-HGL accumulation, which causes severe necrosis of the leaves. Collectively, this functional study provides important insights into the biosynthesis of broad-spectrum anti-herbivore HGL-DTGs and provides evidence consistent with the hypothesis that UGTs play a central role in a plant’s ability to manage the “toxic waste dump” problem of chemical defense.

### A GLS is required for HGL-DTG production

Geranyllinalool is an acyclic diterpene alcohol that is widely distributed across the plant kingdom, occurring in several essential oils (Sandeep and Paarakh, 2009). Geranyllinalool is a precursor for both HGL-DTGs in solanaceous species and for the volatile C_16_-homoterpene 4,8,12-trimethyltrideca-1,2,7,11-tetraene (TMTT), which is emitted from the foliage of a wide range of plant species including *S. lycopersicum* (Ament et al., 2004), maize (*Zea mays*; Hopke et al., 1994), *M. truncatula* (Leitner et al., 2010), and *A. thaliana* (Van Poecke et al., 2001). Although geranyllinalool is present in many plant species, the enzymes responsible for its biosynthesis have been discovered only recently in *A. thaliana* (Herde et al., 2008), *S. lycopersicum*, and *N. attenuata* in which *GLS* is constitutively expressed in leaf and flower tissues and is induced by methyl jasmonate treatment (Falara et al., 2014). Here we provide additional in vivo evidence demonstrating the function of NaGLS in HGL-DTG biosynthesis in *N. attenuata* plants.

### 17-HGL-DTG rhamnosyltransferase activity of UGT91T1

Very little is known about the physiological function of rhamnosylation in specialized metabolism. Hsu and colleagues suggested that rhamnosylation is an essential step in the biosynthesis of lobelinin and is therefore responsible for the color of Lobelia flowers (Hsu et al., 2017). Rhamnosylation of flavonols is thought to modulate auxin homeostasis in *rol1-2* mutants of *A. thaliana* (Kuhn et al., 2016). However, the enzymatic characterization of UDP-rhamnosyltransferases is still thwarted by the high costs of UDP-rhamnose (UDP-Rha). Despite important efforts in the establishment of efficient UDP-Rha production systems (Irmisch et al., 2018), only a few UDP-rhamnosyltransferases have been functionally characterized, and enzymatic assays remain challenging (Mo et al., 2016, Irmisch et al., 2018). Here, we show that UGT91T1 shares a high degree of sequence similarity with functionally characterized rhamnosyltransferases for flavonoids and anthocyanins (CmF7G12RT, GmF3G6R, and PhA3ART–Supplemental Figure 6), is tightly and tissue-specifically co-regulated with the accumulation patterns of rhamnosylated HGL-DTGs, and exhibits strong co-expression with *GLS*, *GGPPS*, and *UGT74P3*. Silencing of *UGT91T1* expression in *N. attenuata* resulted in the almost complete loss (88.5%–98.8% in different tissues compared to WT, Supplemental Data Set 12) of rhamnosylated HGL-DTGs. These changes in HGL-DTG rhamnosylation pattern suggest that UGT91T1 is responsible for the rhamnosylation at the C′-4 hydroxyl-group of glucose on both the C-3 and the C-17 hydroxyl-group of the aglycone. Due to the high biological variability among transiently silenced plants and the complexity of the HGL-DTG profile in *N. obtusifolia*, which produces lower levels of rhamnosylated HGL-DTGs, the biochemical function of No*UGT91T1-like*, the orthologue of UGT91T1 in *N. obtusifolia*, could not be conclusively evaluated.

### 17-HGL-DTG glucosyltransferase activity of UGT74P3 and UGT74P4

Both UDP-glycosyltransferases clustered together in the UGT74 family, which belongs to phylogenetic group L and includes glycosyltransferases that are responsible for the glucosylation of indole-3-acetic acid in *Z. mays* (Szerszen et al., 1994) and *A. thaliana* (Jin et al., 2013). Importantly, additional members of this family show catalytic activity toward diterpene and triterpene glycosides. For example, UGT74M1 glucosylates the carboxylic acid moiety of the triterpene gypsogenic acid (Meesapyodsuk et al., 2007), UGT74-345-2 is involved in the glucosylation of mogroside (Itkin et al., 2016, 2018), and UGT74G1 catalyzes the glucosylation of cyclic DTGs in *S. rebaudiana* (Richman et al., 2005). Both UGT74P3 and UGT74P4 are closely related to UGT74G1 and CsGLT2; these UGTs are responsible for crocetin glucosylation in *C. sativus* (Moraga et al., 2004).

Recombinant enzyme activity assays demonstrated that UGT74P3 and UGT74P4 catalyze the transfer of glucose to the C-3 and C-17 hydroxyl groups of 17-HGL in order to form the putative intermediate products 3-*O*-glucopyranosyl-17-HGL (G-3-HGL) and 17-*O*-glucopyranosyl-17-HGL (G-17-GHL; Figure 4), as well as lyciumoside I, in which both hydroxyl groups are glucosylated. Metabolic profiling of transiently and stably silenced lines provided further evidence that UGT74P3 and UGT74P4 are essential for the formation of lyciumoside I through the attachment of two glucose moieties, as well as the formation of compounds further downstream in the HGL-DTG biosynthetic pathway (lyciumoside II, nicotinanoside III, attenoside). We also observed an accumulation of the novel intermediate HGL-DTGs, which lacked the glucosylation either at the C-3 or C-17 hydroxyl group of the aglycone, as well as the 17-HGL aglycone itself in both stably and transiently silenced lines, suggesting that *N. attenuata* is unable to reroute the excess of 17-HGL.

In contrast to UGT74P3 and UGT74P4, we were not able to fully ascertain that UGT74P5 in *N. attenuata* and its ortholog in *N. obtusifolia* function in HGL-DTG metabolism. Under enzyme assay conditions optimized for the two former enzymes, when incubated with 17-HGL, UGT74P5 produced an extremely low-intensity signal that could correspond to a mono-glucosylated conjugate. However, further work would be needed to evaluate the possibility that UGT74P5 contributes more significantly to the biosynthesis of HGL-DTGs, for instance by glucosylating HGL-DTGs not tested as substrates in this work. Based on the enzymatic assays, we inferred that the reduction of lyciumoside I and the accumulation of G-3-HGL, G-17-HGL, and 17-HGL directly result from the silencing of *UGT74P3*, leaving it unclear whether UGT74P5 is involved in the synthesis of HGL-DTGs or has a still-unknown enzymatic function beyond diterpene metabolism. Notably, the enzymatic assay using both recombinant proteins ruled out the possibility that UGT74P3 and UGT74P5 catalyze consecutive glucosylation steps. Additionally, the co-silencing of *UGT74P5* and *UGT74P3* expression in the stable and transiently silenced lines (Figure 6; Supplemental Figure 8), due to the high sequence similarity of both UGTs, did not allow us to further characterize the specific function of UGT74P5. Hence, the metabolic alterations observed in these lines likely reflect the silencing of both genes.

### Phytotoxicity observed in *UGT74P3*/*UGT74P5* silenced lines

In addition to the striking shifts in HGL-DTG metabolism detected in the stably and transiently silenced lines impaired in *UGT74P3* and *UGT74P5* expression, we also observed strong morphological alterations that ranged from small deformed or thicker succulent leaves to numerous stalled flower buds as well as necrotic spots, stunted growth, and apical meristem necrosis.

The loss of glycosylation results in the ectopic accumulation of hydrophobic aglycones, which leads to a wide range of morphological alterations and growth retardation. Some of the best examples come from the biosynthesis of steroidal alkaloids. Silencing *GAME-1*, encoding a UDP-galactosyltransferase responsible for the glycosylation of steroidal alkaloids, results in severe developmental defects due to the altered sterol composition in membranes triggered by the accumulation of tomatidine (Itkin et al., 2011). Moreover, alterations of the glycosylation of the saponin hederagenin severely affected growth in *M. truncatula* (Naoumkina et al., 2010). The loss-of-function mutants *sad3* and *sad4* in *A. sativa* accumulate the intermediate monodeglucosylated DTG avenacin A-1, which disrupts membrane trafficking, resulting in epidermal degeneration and reduced root hair formation (Mylona et al., 2008). Knocking out *UGT74B1*, encoding a UGT responsible for glucosinolate biosynthesis in *A. thaliana*, leads to the accumulation of toxic levels of thiohydroximates, increased auxin levels in seedlings, and a chlorotic phenotype (Grubb et al., 2004).

There are several possible explanations for the severe growth defects, stalled flower buds, and necrosis we observed in *UGT74P3/UGT74P5*-silenced plants. First blocking HGL-DTG glycosylation might increase the endogenous pool of UDP-glucose, resulting in the preferential synthesis of other glycosides or the accumulation of other compound classes (Naoumkina et al., 2010). This might influence hormone homeostasis and therefore contribute to the severe alterations (Grubb et al., 2004; Kuhn et al., 2016). Auxin levels, which might mediate some of the observed developmental changes, were only altered in *GGPPS*-silenced plants but not in the UGT-silenced lines showing the morphological defects. Alterations of the UDP-glucose pool can also influence UDP-glucuronic acid (UDP-GlcA) biosynthesis. Reduced UDP-GlcA levels in the *ugd2* and *ugd3* mutants of *A. thaliana* lead to swollen cell walls and developmental defects associated with changes in the pectic network (Reboul et al., 2011). IR*ugt74p5* and IR*ugt74p3/ugt74p5* plants exhibited reduced glucuronic acid levels (Supplemental Data Set 9), suggesting that UDP-GlcA production is downregulated, which could mediate the smaller leaves and dwarfish growth phenotype observed in these lines. However, we observed a similar reduction in glucuronic acid levels in *GGPPS*-silenced plants, which did not show these altered growth effects. Alternatively, *UGT74P3/UGT74P5*-silenced plants also exhibited a dramatic increase in levels of intermediate HGL-DTGs and the aglycone 17-HGL compared to other transgenic lines that did not show any morphological phenotypes (Figure 8A). These results suggest that the accumulation of these intermediates may be toxic for the plant.

When *UGT74P3* and *UGT74P5* were silenced in the background of stably silenced IR*ggpps* lines, which do not produce the 17-HGL precursor, no developmental abnormalities were detected, confirming the notion that the developmental abnormalities are associated with the altered HGL-DTG metabolism and not due to unexpected off-target effects (Senthil-Kumar and Mysore, 2011). The application of a synthetic mixture of 17-HGL isomers to leaves of *N. attenuata* resulted in necrotic lesions that pheno-copied those observed in IR*ugt74p5* and IR*ugt74p3/ugt74p5* plants. IR*ggpps* plants, which show strongly reduced HGL-DTG accumulation, were more susceptible to the toxic effects of the exogenous application of 17-HGL than WT plants and showed necrotic lesions in response to 17-HGL concentrations lower than those observed in IR*ugt74p3/ugt74p5* plants. The weaker toxic effects of 17-HGL observed in WT leaves may be due to the stronger chemical sequestration of 17-HGL by active glycosylation. Interestingly, *N. sylvestris* accumulates 17-HGL without producing the glycosylated forms (Heiling et al., 2016), suggesting that other species might be tolerant to 17-HGL or store this compound in special compartments. In short, silencing *UGT74P3* and *UGT74P5* resulted in pleiotropic morphological affects that are associated with the accumulation of the phytotoxic aglycone 17-HGL.

### Defensive function of HGL-DTGs

HGL-DTGs have been studied for more than two decades and are described as potent anti-herbivore defense compounds (Heiling et al., 2010) with deterrent (Jassbi et al., 2006) and resistance (Snook et al., 1997) effects against herbivores. However, so far, it remains unclear which of the many HGL-DTGs, their structural components or post-ingestive modifications account for their mode of action. The impressive structural diversity of HGL-DTGs in *N. attenuata* plants results largely from glycosylation and malonylation reactions. While all malonyl decorations are instantaneously lost in the alkaline pH environment of the midgut of *M. sexta* larvae and therefor may play more important roles in planta (Poreddy et al., 2015; Li et al., 2018), glycosylation leads to a constant and stable pool of potential defensive compounds. Larvae feeding on leaf disks of transgenic IR*ggpps* plants grew larger than larvae feeding on WT leaves, indicating that the overall abundance of HGL-DTGs is a major factor in the plant’s resistance against *M. sexta* (Figure 9), which is consistent with earlier findings (Jassbi et al., 2008, 2010; Heiling et al., 2010). The reduced growth of larvae feeding on leaf disks of transgenic IR*ugt74p5* and IR*ugt74p3/ugt74p5* plants, which have higher total levels of overall HGL-DTGs, is also consistent with the inference that the total abundance of HGL-DTGs is defensively relevant. *N. obtusifolia* has constitutive levels of HGL-DTGs that are five times higher than those of *N. attenuata*, and *M. sexta* larval performance is five times lower after 22 days of feeding on *N. obtusifolia* (Jassbi et al., 2010). However, the HGL-DTG profile in *N. obtusifolia* is structurally different (Heiling et al., 2016), suggesting that the composition or degree of glycosylation is central for the plant’s defense.

Importantly, the specific structures responsible for the mode of action of HGL-DTGs remain unclear. Feeding of purified lyciumoside IV, the most abundant HGL-DTG in the leaves of *N. attenuata*, causes mortality in *M. sexta* larvae silenced in *β-glucosidase 1* (*BG1*) expression (Poreddy et al., 2015). However, the single-compound feeding experiment does not reflect the chemical diversity of HGL-DTGs that is normally consumed by larvae when they feed on plants. While they consumed less leaf tissue, *M. sexta* larvae grew normally when feeding on leaf disks of transgenic IR*ugt91t1* plants, which have reduced levels of lyciumoside IV but increased levels of the non-rhamnosylated HGL-DTGs lyciumoside I and II. Furthermore, we observed increased levels of novel intermediate HGL-DTGs and the aglycone 17-HGL in IR*ugt74p5* and IR*ugt74p3/ugt74p5* plants, which showed a strong antifeedant/deterrent effect (Figure 9B) and reduced growth performance of *M. sexta* larvae (Figure 9A). The differential consumption and growth performance responses on transgenic plants with different HGL-DTG profiles suggest that both pre- and post-ingestive resistance mechanisms may be at play. Given the severe metabolic and pleiotropic morphological phenotypes of IR*ugt74p5*, IR*ugt74p3/ugt74p5*, and IR*ggpps* plants, it is challenging to draw strong inferences about the defensive functions of specific intermediates in the HGL-DTG biosynthetic pathway.

We are still in the early stages of understanding how plants solve the “toxic waste dump” problem. Two common solutions are well documented: (1) Plants produce metabolites (e.g. nicotine) that are specifically toxic to tissues or organs that they lack (e.g. nervous systems and neuro-muscular junctions populated with nicotinic acetylcholine receptors) and (2) plants sequester pro-toxins apart from their toxin-releasing lytic enzymes (e.g. compartmentalization of cyanogenic glycosides and glucosinolates and their active enzymes). However, these examples are likely to be special cases, and the solutions that plants have evolved to solve the “toxic waste dump” problem for the majority of their toxic-defensive metabolites lie in the details of their biosynthetic pathways. Here we advanced our understanding of the toxicity of HGL-DTGs by showing that glucosylation plays a central role in *N. attenuata*’s solution of maintaining a HGL-DTG-based defense. Extending the metabolomics analyses to the “digestive duet” that occurs between plant and insect (“frassomics”) will allow for a better understanding of the post-ingestive fate of DTGs in larval guts.

## Materials and methods

### Plant material and growth conditions

Seed germination and growth condition were described previously (Krugel et al., 2002). Seeds of the 31st generation of an inbred line of *N. attenuata* Torr. Ex. Watts were used as the wild-type in all experiments. Seeds of the following stably silenced inverted repeat (IR) plants impaired in HGL-DTG biosynthesis were used: IR*ugt91t1* Line A: A-11-538-05, IR*ugt91t1* Line B: A-11-538-09, IR*ugt74p5* Line A: A-11-544-09, IR*ugt74p5* Line B: A-11-544-11, IR*ugt74p2/ugt74p5*: A-12-076-07; IR*ggpps*: A-08-230-5 (Heiling et al., 2010). *Nicotiana obtusifolia* plants were cultivated under the same growth conditions except that liquid smoke was not applied to the seeds. Plants for VIGS were transferred after 20 days to a York Chamber at 22°C under a 16-h light (OSRAM Cool White L58W/640 and OSRAM FLUORA L36W/77)/8-h dark cycle.

### Manduca *sexta* growth conditions

Manduca *sexta* eggs were obtained from an in-house colony in which insects are reared in a growth chamber (Snijders Scientific, Tilburg, Netherlands, http://www.snijderslabs.com) at 26°C:16-h light and 24°C:8-h dark, 65% relative humidity, until hatching.

### Performance assays

Freshly hatched neonates were fed leaf disk material taken from the between-vein laminal tissue of the four lowest stem leaves of 43-day-old flowering *N. attenuata* plants (WT, IR*ugt91t1*, IR*ugt74p5*, IR*ugt74p2/ugt74p5*, IR*ggpps*) in round plastic PE-packing cups. Leaf disk material was exchanged every 2 days until day 6 and then exchanged every day until day 12.

### Plant transformation and screening of stably silenced *N. attenuata* plants

Transformation of *N. attenuata* was performed as described in Krugel et al. (2002) using the pRESC8 vector (Gase and Baldwin, 2012) containing the hygromycin phosphotransferase II gene (*hptII*) from pCAMBIA-1301 (GenBank AF234297) and a 306 bp long fragment for *UGT91T1*, a 310-bp long fragment for *UGT74P3*, or a 295-bp long fragment for *UGT74P5*. All primers are shown in Supplemental Data Set 13. Diploid plants were selected by flow cytometry of leaf material of elongated *N. attenuata* transformants performed on a CCA-II flow cytometer (Partec, http://www.partec.com) as described by Bubner et al. (Bubner et al., 2006). Afterward, seeds were collected and individuals with the T-DNA insertion were selected for hygromycin resistance by adding 35 mg L^−1^ hygromycin B to the germination medium. After 10 days, the ratio of seedlings surviving the antibiotic treatment was determined. Seedlings were chosen with a survival rate of 50–90%, and 12 T1 plants per line were checked for T-DNA insertion. To confirm the integrity of the T-DNA insertion, we performed a diagnostic PCR using the primer pairs PROM FOR/INT REV and INT FOR/TER REV (Gase et al., 2011). Genomic DNA (gDNA) was isolated from leaves of *N. attenuata* using a modified cetyltrimethylammonium bromide method (Bubner et al., 2004). PCR was performed using DreamTaq^TM^ DNA-Polymerase (Fermentas, http://www.fermentas.com) according to the instructions of the manufacturer with 1 µg of gDNA. Homozygosity of T2 plants was determined by screening for resistance to hygromycin B. To confirm single insertions, we performed a DNA gel blot analysis as described by Jassbi et al. (2008), except that a 287 bp *hptII* probe obtained by PCR with primer pair (HYG1-18/HYG2-18) was used (Gase et al., 2011). Labeling was performed with the GE Healthcare (http://www.gehealthcare.com) Readyprime DNA labeling system and ProbeQuant g-50 microcolumn according to the manufacturer’s protocol; 10.5 µg of gDNA was digested with the restriction enzymes EcoRV and XbaI from New England Biolabs (http://www.neb.com) and blotted onto a nylon membrane (GeneScreenPlus; Perkin Elmer, http://www.perkinelmer.com) according to the manufacturer’s instructions.

### VIGS

Vector construction, plant growth, and inoculation conditions for VIGS were as described by Saedler and Baldwin (Saedler and Baldwin, 2004). Briefly, 200- to 300-bp fragments of *N. attenuata* and *N. obtusifolia* target genes were amplified by PCR using primer pairs as listed in Supplemental Data Set 13. Amplified fragments were cloned in the vector pTV00 (Ratcliff et al., 2001). *Agrobacterium tumefaciens* strain GV3101 was transformed by electroporation with the resulting plasmids. We used the empty pTV00 vector as a negative control in all experiments. Four leaves of 24- to 27-day-old *N. attenuata* and 25-day-old *N. obtusifolia* plants were infiltrated with a 1:1 mixture of *A. tumefaciens* transformed with pBINTRA (Ratcliff et al., 2001) and one pTV00 derivative carrying a fragment of a gene of interest. pTVPDS, targeting *Phytoene desaturase* (*PDS*), was used as a positive control to monitor silencing progress. Due to the depletion of carotenoids, silencing the PDS gene causes bleaching of tobacco leaves. VIGS-silenced plants were treated 14 days after inoculation, when the bleaching phenotype was fully established in the pTVPDS plants.

### Plant treatment

In order to analyze the regulatory function of jasmonate signaling on HGL-DTG biosynthesis, petioles of five elongated plants (38 days old) were treated with either 20 µL lanolin paste containing 150-µg methyl jasmonate (Lan + MeJA) or with 20 µL pure lanolin (Lan). Treated leaves were harvested from elicited and unelicited plants at 72 h after treatment, flash-frozen in liquid nitrogen, and stored at −80°C until use.

### Determination of 17-HGL phytotoxicity

To determine the phytotoxic effect of 17-HGL, we used 32-day-old early elongated *N. attenuata* WT plants (*N* = 3) and 48-day-old flowering *N. attenuata* plants impaired in *GGPPS* expression (*N* = 5). Three leaves of each plant were inoculated with either 20 µL DMSO, DMSO with 140 nmol HGL, DMSO with 280 nmol HGL, or DMSO with 9800 nmol HGL. The damaged leaf tissue was analyzed after 24 h using ImageJ (Fiji, https://fiji.sc).

### RT-qPCR analysis of transcript levels

Total RNA was extracted from an aliquot of ∼200 mg of powdered leaf material of *N. attenuata* and *N. obtusifolia* ground in liquid nitrogen following the protocol of Kistner and Matamoros (2005). DNase treatment was performed using a TURBO DNA-free™ kit (Invitrogen). RNA quality was checked on a 1% agarose gel and the concentration was determined spectrophotometrically at 260 nm. A total of 1 µg of DNA-free RNA was reverse transcribed using oligo(dT)18 primers and the SuperScript II enzyme (Invitrogen) following the manufacturer’s recommendations. All RT-qPCR assays were performed using Taykon^TM^ No ROX SYBR^®^ Master Mix dttp Blue (Eurogenetics, http://www.eurogentec.com) on a Stratagene MX3005P instrument (http://www.stratagene.com) as recommended by the manufacturer. To normalize transcript levels, primers specific for the *Nicotiana attenuata elongation factor-1α* gene (EF1-α; accession no. GBGF01000210.1) were used. Specific primers in the 5′- to 3′-direction used for SYBR Green-based analyses are listed in Supplemental Data Set 13.

### Heterologous expression of UDP-glucosyltransferases and enzymatic activity assays

The four UGT cDNAs coding for NaUGT74P3, NoUGT74P4, NaUGT74P5, and NoUGT74P6 were cloned into a Gateway^®^ pDEST17 expression vector (ThermoFisherScientific, http://www.thermofisher.com) using pET28a EV as a control. Integrity of the sequence was checked by Sanger-sequencing using an ABI PRISM 3130 Genetic analyzer (AppliedBiosystems, http://www.thermofisher.com) and the appropriate gene-specific primers (Supplemental Data Set 13).

The resulting plasmid was transformed into BL21 (DE3) *E. coli*, which is optimized for the expression of eukaryotic genes; 50 mL LB medium containing 50 µg mL^−1^ carbenicillin was inoculated with 500 µL of an overnight culture corresponding to each candidate gene. Cultures were grown at 37°C until the OD_600_ reached 0.6. Protein expression was induced by adding 1 mM IPTG and incubation at 18°C overnight. The cells were harvested by centrifugation for 10 min at 4,500*g*. The pellet was suspended in 10 mL ice cold lysis buffer [50 mM Tris-HCl pH 7.5, 1% Triton 100, 200 mM NaCl, 1 mg mL^−1^ lysozyme, and 1 tablet of Protease inhibitor Cocktail (Roche)] and sonicated six times for 10 s with 10 s pauses at 200–300 W, followed by centrifugation at 10,000*g* for 60 min. The supernatant was purified using Ni-NTA agarose (Qiagen) in accordance with the manufacturer’s instructions. The purified protein was desalted using an Amicon Ultra-15 Centrifugal Filter Unit (Merck) and the desalted protein was used for activity assays. Reactions were performed in 100-μL reaction volumes containing 100 mM Tris–HCl pH 7.5, 2.5 µg protein, 5 mM UDP-α-d-glucose (Calbiochem, http://www.merckmillipore.com), and 250 µg mL^−1^ 17-HGL for 3 h at 30°C. The reaction was stopped by adding 400 µL methanol, and the mixture was used for HGL-DTG analysis using a high resolution time-of-flight mass spectrometer.

### Quantification of primary metabolites and phytohormones in plant tissues

For the quantitative analysis of primary metabolites and phytohormones, we used leaf material of 42-day-old elongated *N. attenuata* transgenic lines silenced in *GGPPS*, *UGT91T1*, *UGT74P5*, and *UGT74P3/UGT74P5* expression as well as WT plants. Sample preparation and analysis of primary metabolites and phytohormones were performed based on Schafer et al. (2016). Peak integration was performed using MS Data Review software (Bruker Daltonics). For the quantitative analysis of GPP, FPP, and GGPP, we followed the protocol developed by Nagel et al. (2014).

### Quantification of 17-HGL in plant tissues

Approximately 50 mg of leaf material was flash frozen, ground in liquid nitrogen, and aliquoted. Each aliquot was extracted with 500 µL 80% methanol containing 0.2 ng µL^−1^ testosterone and shaken twice at 1,000 strokes for 45 s using a GenoGrinder 2000 (SPEX SamplePrep, http://www.spexsampleprep.com/). Homogenized samples were then centrifuged at 16,000*g* for 20 min at 4°C. The supernatant was centrifuged again at 16,000*g* for 20 min at 4°C and diluted 1:10 with 80% methanol. We established a chromatographic method using a mixture of solvent A: water (Milli-Q, Merck, http://www.emdmillipore.com) with 0.1% acetonitrile and 0.05% formic acid and solvent B: methanol. U(H)PLC for the quantification of 17-HGL was performed using a Zorbax Eclipse XDB-C18 column (3.0 × 50 mm, particle size 1.8 µm) from Agilent Technologies (http://www.agilent.com). The chromatographic separation was achieved using a U(HPLC) Advance (Bruker Daltonics) with the following gradient: 0–0.5 min at 10% of B, 0.5–1 min up to 90% of B, 1–4 min up to 100% of B, 4–5 min at 100% of B, 5–5.05 min down to 10% of B, and from 5.05–6 min at 10% of B. The injection volume was 5 µL and the flow rate 0.5mL min^−1^.

MS detection was performed on an EvoQ Elite triple quadrupole-MS equipped with a heated electrospray ionization (HESI) ion source (Bruker Daltonics) and the following HESI conditions: spray voltage 4500 V, cone temperature 350°C, cone gas flow 35^a^ (arbitrary units), heated probe temperature 500°C, probe gas flow 60^a^, and nebulizer gas 60^a^. Compounds were detected in multiple reaction monitoring mode using specific precursor ion/product ion transitions after positive ionization: the [M-H_2_O+H]^+^ ion was used as precursor for 17-HGL, 289/81 (quantifier), and 289/107 (qualifier); the [M + H]^+^ ion was used as precursor for testosterone, 289/97 (quantifier), and 289/109 (qualifier). Further details are given in Supplemental Figure 23. Peak areas were analyzed using MS Data Review operating software from Bruker Daltonics (http://www.bruker.com).

### Structural determination by nuclear magnetic resonance spectroscopy

17-HGL was provided by HPC24 Standards (www.hpc-standards.com) and the general structure was verified by 1D and 2D nuclear magnetic resonance (NMR) spectroscopy (for ^1^H NMR, see Supplemental Figure 12). A Bruker AVANCE 400 NMR spectrometer (Bruker, Rheinstetten, Germany), equipped with a 5 mm BBFO probe, was used to record ^1^H NMR, DEPT 135, ^1^H-^1^H COSY, HSQC, and HMBC spectra in MeOH-*d*_4_ at 300 K. Spectra were processed using TOPSPIN 3.1 (BrukerBiospin).

### Rapid screening of HGL-DTGs via ultrahigh-pressure liquid chromatography/time of flight mass spectrometry

All materials were ground in liquid nitrogen and split into aliquots of 10–100 mg fresh weight (FW), depending on the tissue. Each aliquot was extracted in 100 µL–1 mL extraction solution (80% methanol; ratio 1/10 FW/extraction solution) containing two steel balls by shaking twice at 1,200 strokes/min for 60 s using a Geno/Grinder 2000. Homogenized samples were then centrifuged at 16,000*g* for 20 min at 4°C. The supernatant was centrifuged again at 16,000*g* for 20 min at 4°C. Two independent chromatographic methods were used to resolve HGL-DTGs. Both methods used a mixture of solvent A: water with 0.1% acetonitrile and 0.05% formic acid and solvent B: acetonitrile with 0.05% formic acid. U(H)PLC was performed using a Dionex UltiMate 3000 rapid separation LC system (Thermo Fisher, http://www.thermofisher.com), combined with a Thermo Acclaim RSLC 120 C18 column (particle size 2.2 µm, average pore diameter 120Å, column dimension 2.1 × 150 mm). The gradient e steps were as follows: 0–0.5 min at 10% of B, 0.5–6.5 min up to 80% of B and 6.5–8 min at 80% of B followed by returning to the starting conditions and column equilibration. For method B, the sample gradient steps were as follows: 0–3 min at 10% B, 3–12 min up to 20% B, 12–17 min up to 35% B, 17–23 min up to 40% B, 23–25 min up to 45% B, 25–30 min up to 50% B, 30–40 min up to 90% B and 40–45 min at 90% B, followed by returning to the starting conditions and column equilibration. The injection volume was 2 µL and the flow rate 0.4 mL min^−1^ for method A and B.

MS detection was performed using a micrOTOF-Q II, an Impact II, and a maXis UHR-Q-TOF-MS system (Bruker Daltonics) equipped with an electrospray ionization (ESI) source operating in positive ion mode. ESI conditions for the micrOTOF-Q II system were end plate offset 500 V, capillary voltage 4,500 V, capillary exit 130 V, dry temperature 180°C, and a dry gas flow of 10 L min^−1^. ESI conditions for the Impact II UHR-Q-TOF-MS system were capillary voltage 4,500 V, end plate offset 500 V, nebulizer 2 bar, dry temperature 200°C and a dry gas flow of 8 L min^−1^. ESI conditions for the maXis UHR-Q-TOF-MS system were capillary voltage 4,500 V, end plate offset 500 V, nebulizer 1.8 bar, dry temperature 200°C and a dry gas flow of 8 L min^−1^. MS data were collected over a range of *m/z* from 100 to 1,600. Mass calibration was performed using sodium formate (50 mL isopropanol, 200 µL formic acid, 1 mL 1 M NaOH in water). Data files were calibrated using the Bruker high-precision calibration algorithm. Lock mass calibration was performed for the profiling of the stable lines using signal *m/z* 622.0289 (molecular formula C_12_H_19_F_12_N_3_O_6_P_3_) from the ESI Tuning Mix (Agilent Technologies, http://www.agilent.com). MS/MS experiments were performed using AutoMS/MS runs at various CID voltages from 12.5 to 22.5 eV for ammonium adducts. Instrument control, data acquisition, and reprocessing were performed using HyStar 3.1 (Bruker Daltonics). Molecular formulae were determined using SmartFormula 3D. SmartFormula calculates the elemental compositions from accurate mass as well as the isotopic pattern information using MS (SmartFormula) and MS + MS/MS information (SmartFormula 3D; Krebs and Yates, 2008; Kind and Fiehn, 2010). The mass tolerance was set to 4 mDa, and the filter H/C element ratio was set between 1 and 3. Isotope peaks were assigned using the Simulate Pattern Tool of DataAnalysis software version 4.2 (Bruker Daltonics). We used QuantAnalysis (Bruker Daltonics) to integrate the peak areas.

### Dereplication of HGL-DTGs

The dereplication workflow relies on a comprehensive MS and MS/MS database constructed by our group for HGL-DTGs of several solanaceous species (Heiling et al., 2016) and a detailed rule-set for the annotation of fragmentation patterns of the different moieties decorating the 17-HGL aglycone. The MS and MS/MS database is based on the retention time and mass spectrometric data of purified HGL-DTGs, which are used as authentic standards. Novel HGL-DTGs are annotated based on their spectral similarity to the MS and MS/MS in-house database. For the visualization and identification of HGL-DTG profiles, we computed the extracted ion chromatogram (EIC) *m/z* 271.2420. This *m/z* fragment corresponds to the 17-HGL aglycone lacking both hydroxyl-groups and is produced by in-source fragmentation during ionization for all HGL-DTGs independently of the type and degree of metabolic decorations. The trace returned for *m/z* 271.2420 allows for the visualization of the complete HGL-DTG chemotype and to rapidly assess variations within this chemotype that result from the single gene manipulations. Following application of the de-replication workflow, we subdivided the chemotype between rhamnosylated and non-rhamnosylated HGL-DTGs based on the presence of diagnostic *m/z* signals. Lyciumosides I and II and their malonylated derivatives corresponded to nonrhamnosylated HGL-DTGS, while lyciumoside IV, attenoside, and nicotianoside III as well as their malonylated forms represented the major rhamnosylated compounds. We provide a detailed description of the identification of known and novel HGL-DTGs in Supplemental Data Set 6. The identification levels are based on community standards reported in (Sumner et al., 2007). Raw MS metabolomics data have been deposited in the open metabolomics database Metabolights, www.ebi.ac.uk/metabolights (accession no. MTBLS1819).

### Statistical analysis

Data were analyzed using Excel (Microsoft, http://www.microsoft.com), SPSS 20.0 (SPSS Inc, http://www-01.ibm.com/software/analytics/spss/) and RStudio (RStudio Inc, https://www.r-project.org) using the package xlsx. Unless otherwise stated, parametric data were compared using ANOVA followed by Fisher LSD/Holm-Bonferroni post hoc tests or Mann–Whitney–Wilcox Pairwise Test (for heteroskedastic data). The phylogenetic tree was constructed using the maximum likelihood and neighbor-joining methods in MEGA5.0 (http://www.megasoftware.net/). Sequence Alignment and machine-readable tree files are provided in Supplemental Data Set 14.]

### Accession numbers

Sequence data for the *N. attenuata* genes used for the analysis of the HGL-DTG pathway can be found in the GenBank database under the following accession numbers: NaGLS, KJ755868; NaGGPPS, EF382626; NaUGT74P3, KX752207; NaUGT91T1, KX752209; NaUGT74P5, KX752208), *N. obtusifolia* (NoUGT74P4, KX752210; NoUGT74P6, KX752211; NoUGT91T1-like, MG051326). All GenBank database accession numbers for the known UGTs and putative *N. attenuata* UGTs can be found in the Supplemental Methods. Microarray data are publicly available at the Gene Expression Omnibus database (accession number GSE30287). The MS metabolomics data set has been deposited in the open metabolomics database Metabolights, www.ebi.ac.uk/metabolights (accession no. MTBLS1819).

## Supplemental data

The following materials are available in the online version of this article.

**Supplemental Figure 1.** Alignment of the UGT C-terminal consensus sequence of 112 family 1 glycosyltransferases from *N. attenuata* and *N. obtusifolia*.

**Supplemental Figure 2.** Phylogenetic analysis of the *N. attenuata* UGT superfamily shows 16 major groups.

**Supplemental Figure 3.** Amino acid composition of all identified UGTs of the superfamily 1 in *N. attenuata.*

**Supplemental Figure 4.** Phylogenetic relationships and herbivory-induced tissue-specific expression of 110 predicted UDP-glycosyltransferases (UGT).

**Supplemental Figure 5.** Transcriptomic variation of UGTs after treatment with OS in *N. attenuate.*

**Supplemental Figure 6.** Phylogenetic tree analysis for the UDP-glycosyltransferases used for stable and transient silencing.

**Supplemental Figure 7.** Silencing efficiency for the three transiently silenced 17-HGL-DTG biosynthetic UGTs in pTVUGT91T1, pTVUGT74P3 and pTVUGT74P5.

**Supplemental Figure 8.** Co-silencing efficiency of UGT74P3 and UGT74P5 in pTVUGT74P3 and pTVUGT74P5.

**Supplemental Figure 9.** Silencing efficiency for the three transiently silenced 17-HGL-DTG biosynthetic UGTs in pTVUGT91T1-like, pTVUGT74P4 and pTVUGT74P6 in *N. obtusifolia*.

**Supplemental Figure 10.** Mass spectrometric characterization and annotation of novel HGL-DTGs in transiently silenced *N. obtusifolia* plants impaired in No*UGT74P4* and No*UGT74P6* expression.

**Supplemental Figure 11.** Characterization and annotation of novel HGL-DTGs via MS/MS in stably silenced *N. attenuata* plants impaired in *UGT74P3* and *UGT74P5* expression.

**Supplemental Figure 12.** ^1^H NMR spectrum of synthetic 17-hydroxygeranyllinalool (17-HGL, HPC24 Standards).

**Supplemental Figure 13.** Morphological characterization of *N. attenuata* plants transiently silenced in *UGT91T1, UGT74P3*, and *UGT74P5* expression.

**Supplemental Figure 14.** Morphological characterization of *N. obtusifolia* plants transiently silenced in No*UGT91T1-like*, No*UGT74P4*, No*UGT74P6*, and No*UGT74P4*/*UGT74P6* expression.

**Supplemental Figure 15.** Metabolite profiling and morphological characterization of *N. attenuata* plants transiently silenced via virus-induced gene silencing (VIGS) of geranyllinalool synthase (GLS).

**Supplemental Figure 16.** DNA gel blot analysis.

**Supplemental Figure 17.** Morphological characterization of the stably transformed *IRugt74p5* Line A, Line B and *IRugt74p3/ugt74p5*.

**Supplemental Figure 18.** Characterization of growth parameters in IR*ugt91t1*, IR*ugt74p5*, IR*ugt74p3/ugt74p5* and IR*ggpps.*

**Supplemental Figure 19.** Morphological characterization of IR*ugt91t1.*

**Supplemental Figure 20.** Overall abundance of HGL-DTGs.

**Supplemental Figure 21.** Disrupting HGL-DTG glycosylation reorganizes general, specialized and hormonal metabolic pathways.

**Supplemental Figure 22.** Characterization of free prenyldiphosphates in IR*ggpps* and WT.

**Supplemental Figure 23.** Quantitative 17-HGL method.

**Supplemental Figure 24.** 17-HGL concentration in IR*ggpps* and WT plants transiently transformed with pTV00, pTVUGT74P3 and pTVUGT74P5.

**Supplemental Figure 25.** Characterization of the morphological phenotypes of WT and IR*ggpps* plants transiently transformed with pTV00.

**Supplemental Figure 26.** Characterization of the morphological phenotypes of WT and IR*ggpps* plants transiently transformed with pTVUGT74P3.

**Supplemental Figure 27.** Characterization of the morphological phenotypes of WT and IR*ggpps* plants transiently transformed with pTVUGt74P5.

**Supplemental Table 1.** Phylogenetic grouping of 107 UGTs in *N. attenuata.*

**Supplemental Table 2.** Molecular weights of UGTs in *N. attenuata.*

**Supplemental Table 3.** SignalIP4.1 – signal peptide cleavage sites.

**Supplemental Table 4.** Pearson Correlation of all UGTs to NaGLS and NaGGPPS.

**Supplemental Methods**

**Supplemental Data Set 1.** UGT Amino acid composition in *N. attenuata.*

**Supplemental Data Set 2.** Relative UGT expression vs. time in *N. attenuata.*

**Supplemental Data Set 3.** HGL-DTG profiles of transiently silenced *N. attenuata* plants.

**Supplemental Data Set 4.** HGL-DTG profiles of transiently silenced NaGLS *N. attenuata* plants.

**Supplemental Data Set 5.** MS/MS Measurements for HGL-DTGs in *N. obtusifolia.*

**Supplemental Data Set 6.** MS/MS Measurements for HGL-DTGs in *N. attenuata.*

**Supplemental Data Set 7.** HGL-DTG profiles of transiently silenced *N. obtusifolia* plants.

**Supplemental Data Set 8.** HGL-DTG profiles of stably silenced *N. attenuata* plants.

**Supplemental Data Set 9.** General and specialized metabolites in stably silenced *N. attenuata* plants.

**Supplemental Data Set 10.** Phytohormone profiles in stably silenced *N. attenuata* plants.

**Supplemental Data Set 11.** Statistical analysis of the performance assay and consumed leaf disk mass.

**Supplemental Data Set 12.** Tissue-specific HGL-DTG modulation.

**Supplemental Data Set 13.** Primers used in this study.

**Supplemental Data Set 14.** Sequence alignment and tree files for phylogenetic analysis.

## Supplementary Material

koab048_Supplementary_Data

## Notes

The authors responsible for distribution of materials integral to the findings presented in this article in accordance with the policy described in the Instructions for Authors (https://academic.oup.com/plcell) are Ian T. Baldwin (baldwin@ice.mpg.de), Emmanuel Gaquerel (http://emmanuel.gaquerel@ibmp-cnrs.unistra.fr), and Sven Heiling (sven_heiling@web.de).
