# Supplementary material for: Specific decorations of 17-hydroxygeranyllinalool diterpene glycosides solve the autotoxicity problem of chemical defense in Nicotiana attenuata
Source: Plant Cell. 2021 Feb 9;33(5):1748–70. doi: 10.1093/plcell/koab048 (PMC8254506; doi:10.1093/plcell/koab048)
Supplement: koab048_Supplementary_Data [file koab048_supplementary_data.zip › tpc.00165.2020-s02.pdf]

Supplemental Data. Heiling et al. (2020). Specific decorations of 17-hydroxygeranyllinalool diterpene glycosides solve the autotoxicity problem of chemical defense in *Nicotiana attenuata*. Plant Cell.

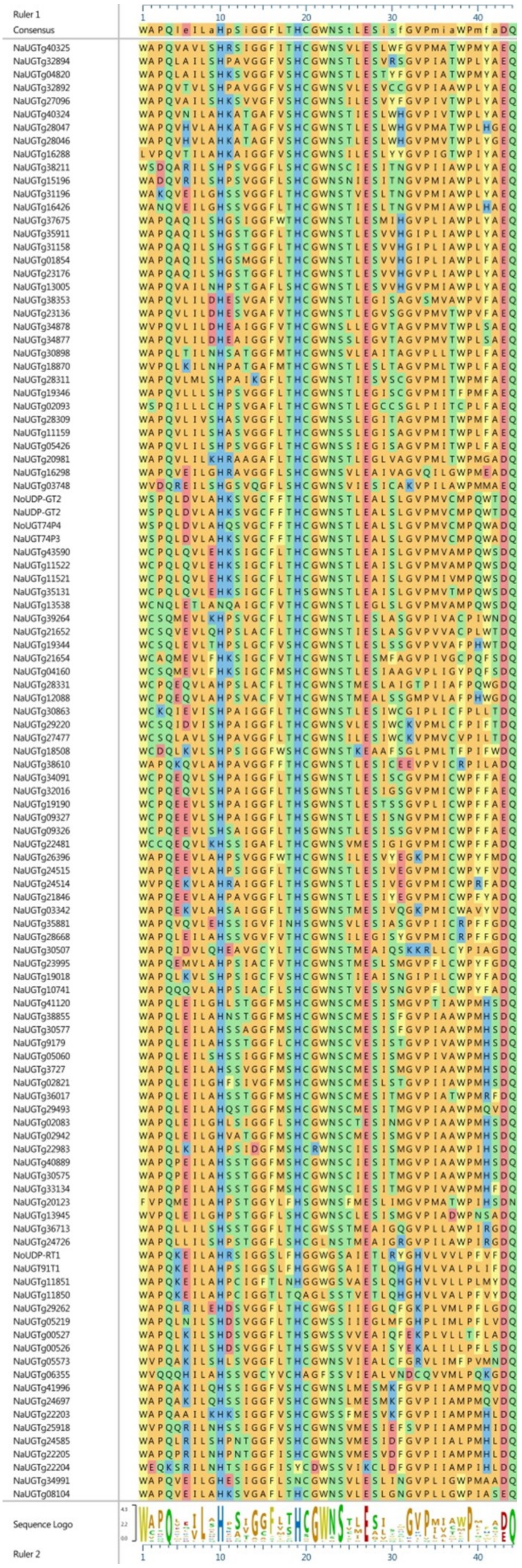

Supplemental Figure 1: Alignment of the UGT C-terminal consensus sequence of 112 family 1 glycosyltransferases from *N. attenuata* and *N. obtusifolia*. (Supports Figures 2, 3 and 5)

To identify members of the UGT family, the 44 amino acids conserved sequence of the PSPG motif was verified using HMMER and aligned using MUSCLE. The consensus sequence is shown in the upper part of the figure and the percentage of similar residues is displayed by the height of the letters in the sequence logo in the lower part of the figure.

Supplemental Data. Heiling et al. (2020). Specific decorations of 17-hydroxygeranyllinalool diterpene glycosides solve the autotoxicity problem of chemical defense in *Nicotiana attenuata*. Plant Cell.

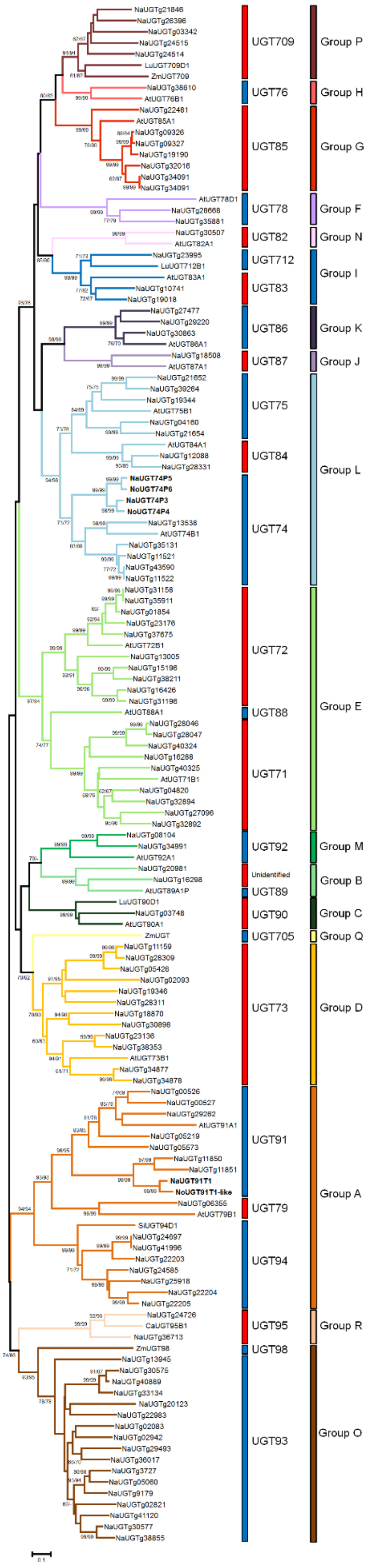

Supplemental Figure 2: Phylogenetic analysis of the *N. attenuata* UGT superfamily shows 16 major groups (Supports Figures 2, 3 and 5)

The phylogenetic tree was obtained by aligning 138 full length amino acid sequences coding for UGTs of the superfamily 1. The phylogenetic relationship was inferred by using either Neighbor-Joining or Maximum Likelihood based on the JTT matrix-based model (both bootstrap = 1000). Bootstrap values over 60% are indicated above the nodes, with the number on the left for the Neighbor-Joining and right for Maximum-Likelihood. 19 *Arabidopsis thaliana*, one *Cicer arietinum*, three *Linum usitatissimum*, one *Sesamum indicum* and three *Zea mays* sequences from each UGT subgroup were included as references in the analysis. Subgroups were created based on sequence similarity (45% similarity for major group, 60% similarity for subgroups) to the reference UGTs and are indicated next to the phylogenetic tree. Sequence similarity to group O was established using ZOG1 and ZOX1 from *Phaseolus vulgaris*. Evolutionary analyses were conducted in MEGA5. All positions containing gaps and missing data were eliminated. NaUGT74P3, NoUGT74P4, NaUGT91T1, NoUGT91T1-like, NaUGT74P5 and NoUGT74P6 analyzed in the present study are in bold letters.

For the construction of the UDP-Glycosyltransferase tree in *N. attenuata*, we used the following Genbank accessions as markers for *Arabidopsis thaliana* (AtUGT71B1 – AB025634; AtUGT72B1 – AC023628; AtUGT73B1 - AT4G34138; AtUGT74B1 - AT1G24100; AtUGT75B1 - AT1G05560; AtUGT76B1 - AT3G11340; AtUGT78D1 - AT1G30530; AtUGT79B1 - AT5G54060; AtUGT82A1 - AT3G22250; AtUGT83A1 - AT3G02100; AtUGT84A1 - AT4G15480; AtUGT85A1 - AT1G22400; AtUGT86A1 - AT2G36970; AtUGT87A1 - AT2G30150; AtUGT88A1 - AT3G16520; AtUGT89A1P – AC006085 (40400-41711); AtUGT90A1 – AC005167 (24146-26230); AtUGT91A1 - AT2G22590; AtUGT92A1 - AT5G12890); *Cicer arietinum* (CaUGT95B1 - gi|533214762); *Linum usitatissimum* (LuUGT709D1 - JN088380; LuUGT712B1 - JN088353; LuUGT90D1 - JN088402); *Sesamum indicum* (SiUGT94D1 - AB333799); *Zea mays* (ZmUGT709 - NP_001148991.1; ZmUGT - NP_001130895.2; ZmUGT98 - NP_001141165.1)

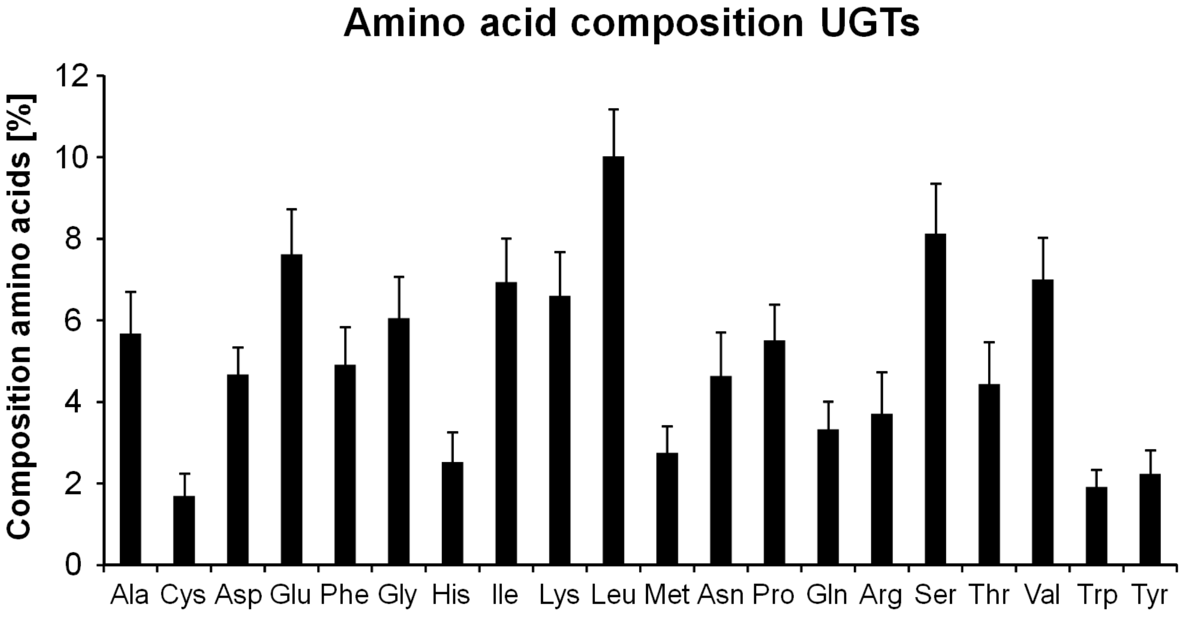

Supplemental Figure 3: Amino acid composition of all identified UGTs of the superfamily 1 in *N. attenuata* (Supports Figures 2)

Shown is the amino acid composition in percentage [%] for all identified UGTs of the superfamily 1 in *N. attenuata*. Error bars represent the standard error of the mean.

Supplemental Data. Heiling et al. (2020). Specific decorations of 17-hydroxygeranyllinalool diterpene glycosides solve the autotoxicity problem of chemical defense in *Nicotiana attenuata*. Plant Cell.

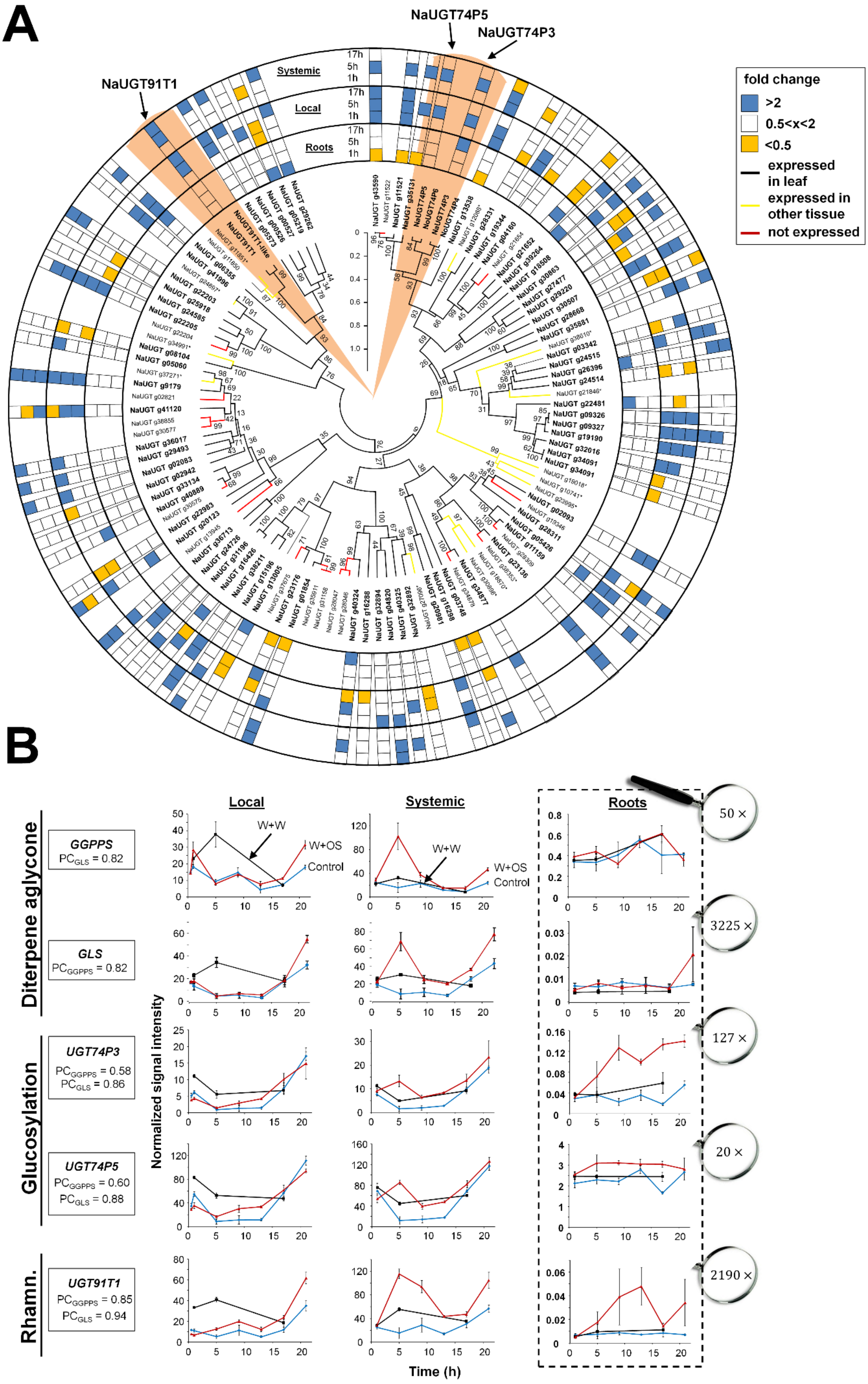

Supplemental Figure 4: Phylogenetic relationships and herbivory-induced tissue-specific expression of 110 predicted UDP-glycosyltransferases (UGT) (Supports Figures 2, 3 and 5)

A) UGT phylogenetic tree generated from the alignment of 107 UGTs from *N. attenuata* and three UGTs from *N. obtusifolia* inferred from the Maximum Likelihood method based on the JTT matrix based model (bootstrap = 1000) (Jones et al. 1982). UGTs were identified based on the presence of the PSPG-Box motif. Shown are the changes in transcript abundance (fold change W+OS vs control: <0.5 and >2) after 1h, 5h and 17h (N=3 biological replicates per time point and per treatment group) resulting from puncture wounds being immediately treated with oral secretions (W+OS) of *M. sexta* larvae in local treated leaves, orthostichous systemic leaves and roots. Expression of 77 UGTs was detected in these tissues, 12 UGTs were detected in other tissue types from an RNAseq tissue atlas and 18 UGTs were not detected in any of the profiled tissues. Light orange sectors highlight the three UGTs characterized in this study for their involvement in HGL-DTG biosynthesis in *N. attenuata* (black). B) Kinetic analysis of W+OS elicited transcript levels of *GGPPS*, *GLS*, *UGT74P3*, *UGT74P5* and *UGT91T1* in locally elicited, systemic leaves and root tissues. Normalized transcript levels were obtained from a previously published full transcriptome microarray experiment (Kim *et al.* 2011). To identify candidate UGTs responsible for HGL-DTG biosynthesis, tissue-level Pearson correlations (PC) were calculated between candidate transcript abundances and those of known (GGPPS, Jassbi *et al.* 2008; GLS, Falara *et al.* 2014) genes in the pathway. *UGT74P3*, *UGT74P5* and *UGT91T1* returned high PC scores with *GLS* in systemic tissues, as did *UGT91T1* with *GGPPS* and *GLS*. Values within the magnifier represent the average magnification factor between root and shoot tissue. Error bars represent the standard deviation of the mean.

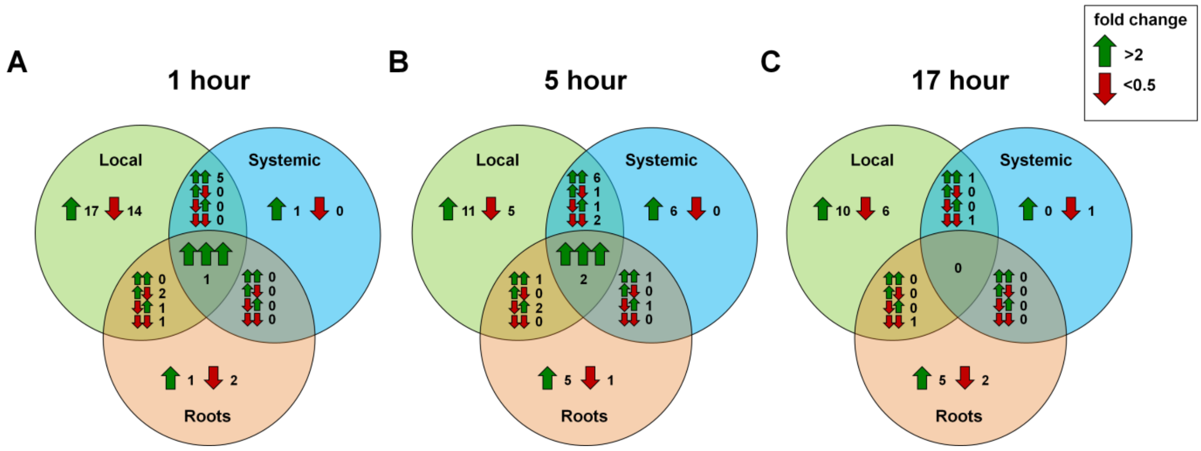

Supplemental Figure 5: Transcriptomic variation of UGTs after treatment with OS in *N. attenuata* (Supports Figure 2)

Venn-diagrams in panel A), B) and C) display the up- and down-regulated UGTs at 1, 5 and 17h after treatment with W+OS in local and systemic leaf tissue as well as in root tissue in *N. attenuata* (N=3). Arrows represent an increase or decrease in the fold change of the expression of UGTs of W+OS-treated vs. Control tissue.

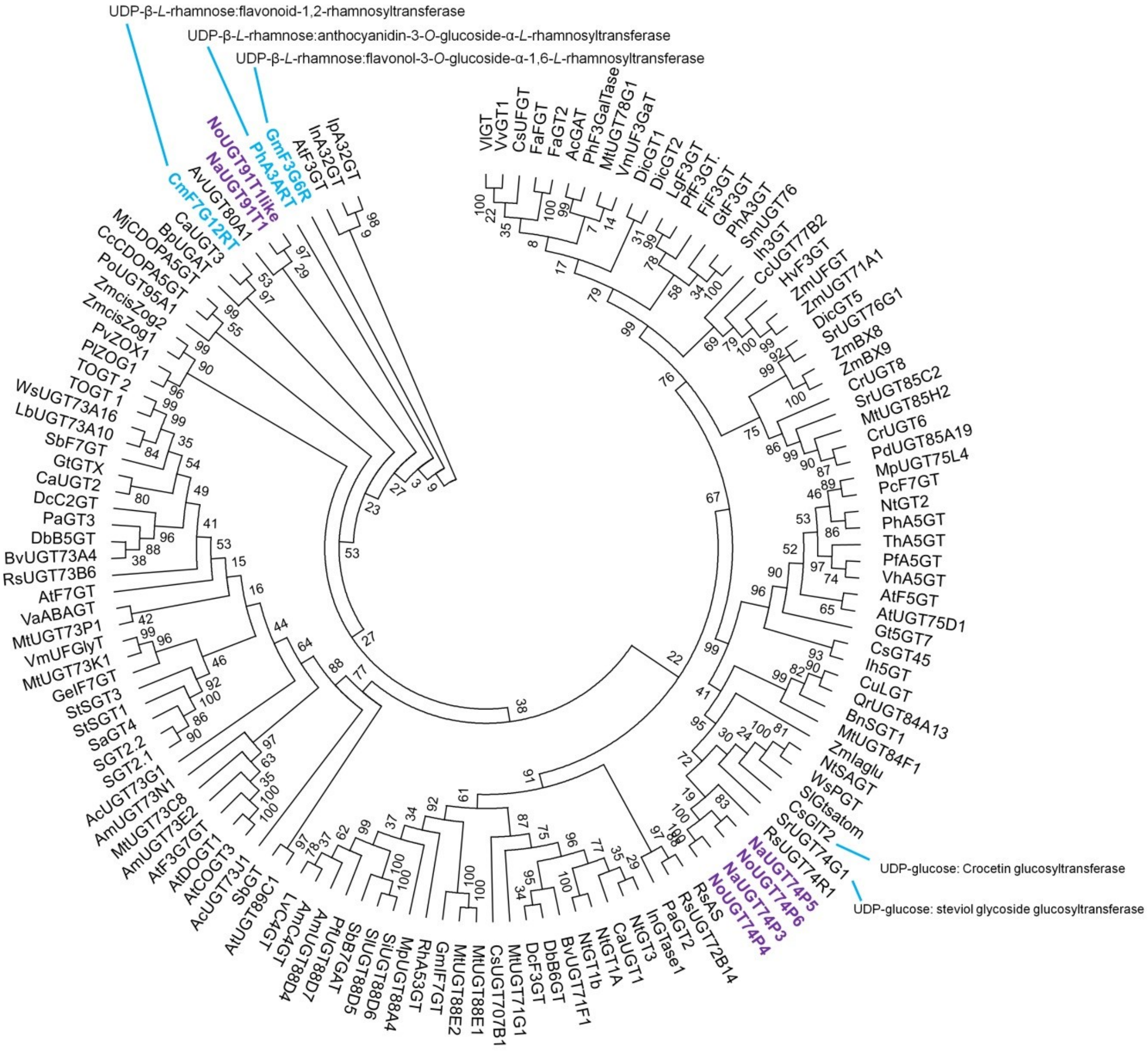

Supplemental Figure 6: Phylogenetic tree analysis for the UDP-glycosyltransferases used for stable and transient silencing. (Supports Figures 2 and 5)

The tree was obtained by aligning characterized glycosyltransferases of the GT superfamily 1 (GT 1, 129 GT amino acid sequences) and inferring their phylogenetic relationship using the Maximum Likelihood method (bootstrap = 1000) based on the JTT matrix-based model. Evolutionary analyses were conducted in MEGA5. NaUGT74P3, NoUGT74P4, NaUGT91T1, NoUGT91T1-like, NaUGT74P5 and NoUGT74P6 analyzed in the present study are highlighted with purple branches. Blue branches indicate closely related UGTs with similar function.

For the construction of the UDP-glycosyltransferase tree in different species, we used the following Genbank accessions for *Allium cepa* (UGT73G1, AAP88406.1; UGT73J1, AAP88407.1), *Antirrhinum majus* (AmC4GT, BAE48239; UGT73E2 (Amugt36), BAG16513.1; UGT73N1 (Amugt38), BAG16514.1; UGT88D4, BAG31945), *Arabidopsis thaliana* (AtF3G7GT, Q9ZQ95; AtF3GT, AAM91339; AtF5GT, AAM91686; AtF7GT, AAL90934; AtUGT89C1, AAP31923; AtDOGT1,

NP_181218; AtUGT75D1, AAB58497.1), *Aralia cordata* (AcGAT, BAD06514), *Avena sativa* (AvUGT80A1, CAB06081), *Bellis perennis* (UGT94B1 (BpUGAT), BAD77944), *Beta vulgaris* (BvUGT71F1, AAS94330; BvUGT73A4, AAS94329.1), *Brassica napus* (UGT84A9 (BnSGT1), AF287143_1), *Catharanthus roseus* (CaUGT3, BAH80312; CaUGT1, BAD29721; CaUGT2, BAD29722; UGT85A2a (CrUGT6), BAK55749; UGT709C2 (CrUGT8),BAO01109), *Celosia cristata* (CcCDOPA5GT, BAD91804), *Citrus maxima* (CmF7G12RT, AAL06646), *Citrus sinensis* (CsUFGT, AAS00612), *Citrus unshiu* (CuLGT, BAA93039), *Crocosmia x crocosmiiflora (*CcUGT77B2, MG938542); *Crocus sativus* (CsGT45, ACM66950.1; CsUGT707B1, CCG85331; Glt2 (UGTCs2), AAP94878.1), *Dianthus caryophyllus* (DcF3GT, BAD52004; DicGT1, BAD52003; DicGT2, BAD52005; DicGT4 (DcC2GT), BAD52006; DicGT5, BAD52007), *Dorotheanthus bellidiformis* (DbB5GT, CAB56231; DbB6GT, AAL57240), *Forsythia x intermedia* (FiF3GT, AAD21086), *Fragaria x ananassa* (FaFGT, AAU12367; FaGT2, AAU09443), *Gentiana triflora* (Gt5GT7, BAG32255; GtF3GT, BAA12737; GtGTX, BAC54092), *Glycine max* (GmF3G6R, BAN91401; GmIF7GT, BAF64416), *Glycyrrhiza echinata* (GeIF7GT, BAC78438), *Hordeum vulgare subsp. vulgar* (HvF3GT, CAA33729), *Ipomoea nil* (In3GGT(InA32GT), BAD95885; InGTase1, BAF75917), *Ipomoea purpurea* (Ip3GGT(IpA32GT), BAD95882), *Iris x hollandica* (Ih3GT, BAD83701; Ih5GT, BAD06874), *Lamium galeobdolon* (LgF3GT, AEB61487), *Linaria vulgaris* (LvC4GT, BAE48240), *Lycium barbarum* (Ugt73a10, BAG80536), *Maclura pomifera* (MpUGT75L4, ABL85474; MpUGT88A4, ABL85471), *Medicago trunculata* (MtUGT73C8, ABI94020; MtUGT73K1, AAW56091; MtUGT73P1, ABI94026; MtUGT78G1, ABI94025; MtUGT84F1, ABI94023; MtUGT85H2, ABI94024.1; MtUGT88E1, ABI94021; MtUGT88E2, ABI94025; MtUGT71G1, AAW56092), *Mirabilis jalapa* (CDOPA5GT, BAD91804), *Nicotiana tabacum* (NTGT1A, BAB60720.1; NTGT1b, BAB60721.1; NtGT2, BAB88935; NtGT3, BAB88934; NtSAGT, AAF61647; TOGT 1, AAK28303; TOGT 2, AAK28304), *Perilla frutescens* (PfA5GT, BAA36421; PfF3GT, BAA19659; PfUGT88D7 (F7GAT), BAG31948), *Petunia x hybrida* (PhA3ART, CAA50376; PhA3GT, BAA89008; PhA5GT, BAA89009; PhF3GalTase, AF165148_1), *Phaseolus lunatus* (PlZOG1, AAD04166); *Phaseolus vulgaris* (PvZOX1, AF116858_1), *Phytolacca americana* (PaGT2, BAG71125; PaGT3, BAG71127), *Pilosella officinarum* (PoUGT95A1, ACB56927), *Prunus dulcis* (PdUGT85A19, ABV68925), *Pyrus communis* (PcF7GT, AAY27090), *Quercus robur* (QrUGT84A13, AHA54051), *Rauwolfia serpentine* (RsAS, CAC35167), *Rhodiola sachalinensis* (RsUGT73B6, AAS55083; RsUGT74R1, ABP49574; RsUGT72B14, ACD87062), *Rosa hybrida* (RhA53GT, BAD99560), *Scutellaria baicalensis* (SbB7GAT, BAD99560; SbF7GT, BAA83484), *Scutellaria laeteviolacea var. yakusimensis* (SlUGT88D5, BAG31946), *Sesamum indicum* (SiUGT88D6, BAG31947), *Solanum aculeatissimum* (SaGT4, BAD89042.1), *Solanum berthaultii* (SbGT, AAB62270.1), *Solanum lycopersicum* (SlGtsatom, CAI62049.1), *Solanum melongena* (SmUGT76, CAA54558.1), *Solanum tuberosum* (Sgt2.1, ABB29873.1; Sgt2.2, ABB29874.1; StSgt1, AAB48444.1; StSgt3, ABB84472.1), *Stevia rebaudiana* (SrUGT74G1, AY345982; SrUGT76G1, AY345974; SrUGT85C2, AY345978), *Torenia hybrida* (ThA5GT, BAC54093), *Verbena hybrida* (VhA5GT, BAA36423), *Vigna angularis* (VaABAGT, BAB83692), Vigna mungo (VmUF3GaT, BAA36972; VmUFGlyT, BAA36410), *Vitis labrusca* (VlGT, ABR24135), *Vitis vinifera* (VvGT1, AAB81682), *Withania somnifera* (WsPGT, FJ560880; WsUGT73A16, FJ654696/ACO44747.1), *Zea mays* (ZmBX8, AF331854_1; ZmBX9, CAX02221; ZmcisZog1, AAK53551; ZmcisZog2, AAL92460; ZmIaglu, AAA59054;

ZmUFGT, CAA30760; ZmUGT71A1, CAA31856).

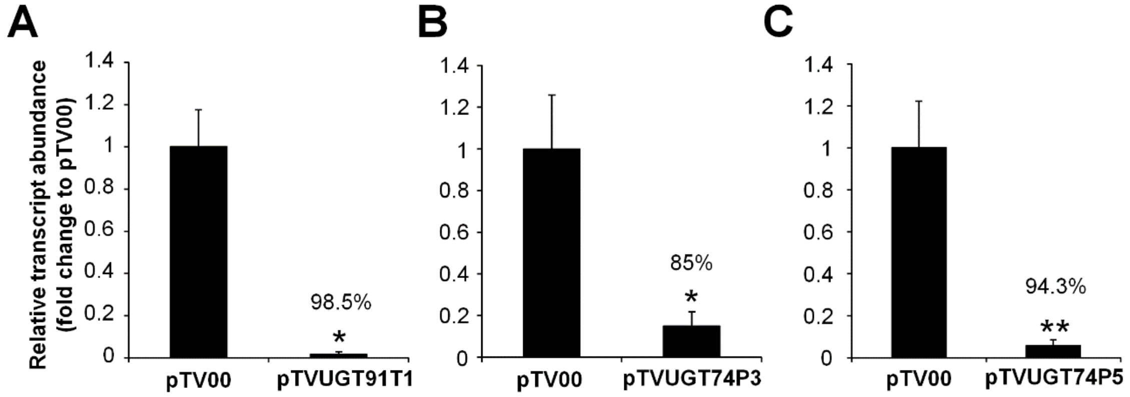

Supplemental Figure 7: Silencing efficiency for the three transiently-silenced 17-HGL-DTG biosynthetic UGTs in pTVUGT91T1, pTVUGT74P3 and pTVUGT74P5. (Supports Figure 2)

Relative transcript abundance (fold change to pTV00 empty vector *N. attenuata* plants after normalization to the transcript abundance of *Elongation Factor 1α* – Na*ELF1α*) of **A)** *UGT91T1*, **B)** *UGT74P3* and **C)** *UGT74P5* in leaves of transiently-silenced *N. attenuata* plants (average ± SE; N=4). Asterisks indicate significant differences between empty vector control (pTV00) and transiently-silenced lines (*t*-test, *P ≤ 0.05, ** P < 0.01).

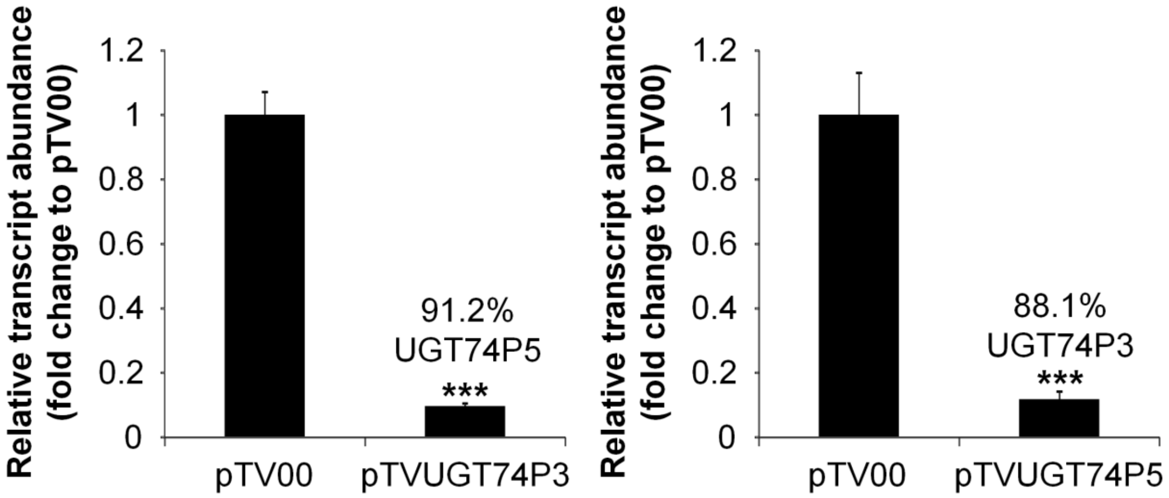

Supplemental Figure 8: Co-silencing efficiency of UGT74P3 and UGT74P5 in pTVUGT74P3 and pTVUGT74P5. (Supports Figure 2)

Relative transcript abundance (fold change to pTV00 empty vector *N. attenuata* plants after normalization to the transcript abundance of *Elongation Factor 1α* – Na*ELF1α*) of **A)** *UGT74P5* in pTVUGT74P3, **B)** *UGT74P3* in pTVUGT74P5 - in buds of transiently-silenced *N. attenuata* plants (average ± SE; N=7). Asterisks indicate significant differences between empty vector control (pTV00) and transiently-silenced lines (*t*-test, *** P < 0.001).

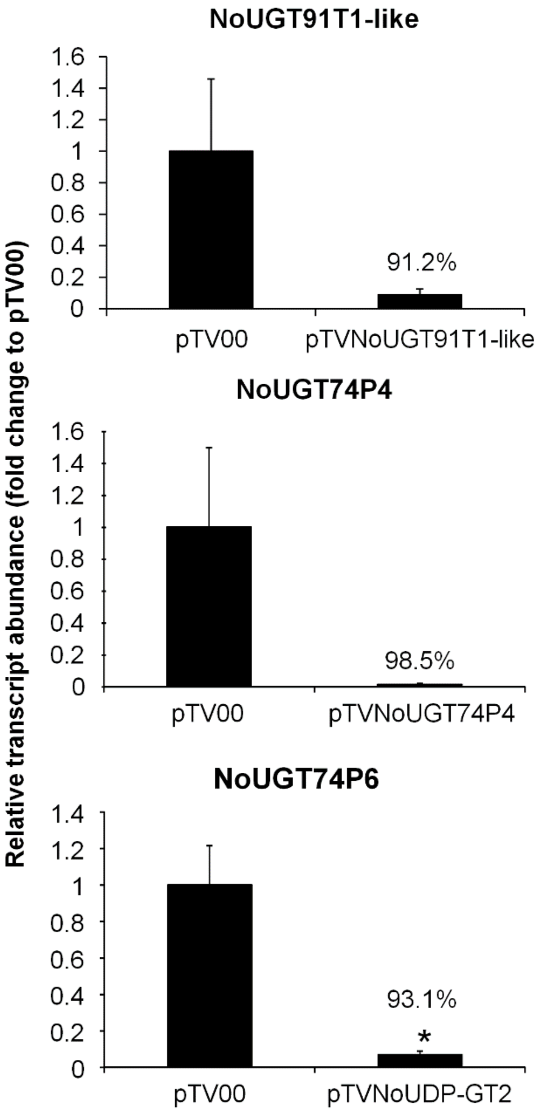

Supplemental Figure 9: Silencing efficiency for the three transiently-silenced 17-HGL-DTG biosynthetic UGTs in pTVUGT91T1-like, pTVUGT74P3 and pTVUGT74P6 in *N. obtusifolia*. (Supports Figure 3)

Relative transcript abundance (fold change to pTV00 empty vector *N. obtusifolia* plants after normalization to the transcript abundance of *Elongation Factor 1α* – No*ELF1α*) of **A)** No*UGT91T1-like*, **B)** No*UGT74P4* and **C)** No*UGT74P6* in leaves of 37-days-old elongated transiently-silenced *N. obtusifolia* plants (average ± SE; N=4). Asterisks indicate significant differences between empty vector control (pTV00) and transiently-silenced lines (*t*-test, *P ≤ 0.05).

Supplemental Data. Heiling et al. (2020). Specific decorations of 17-hydroxygeranyllinalool diterpene glycosides solve the autotoxicity problem of chemical defense in *Nicotiana attenuata*. Plant Cell.

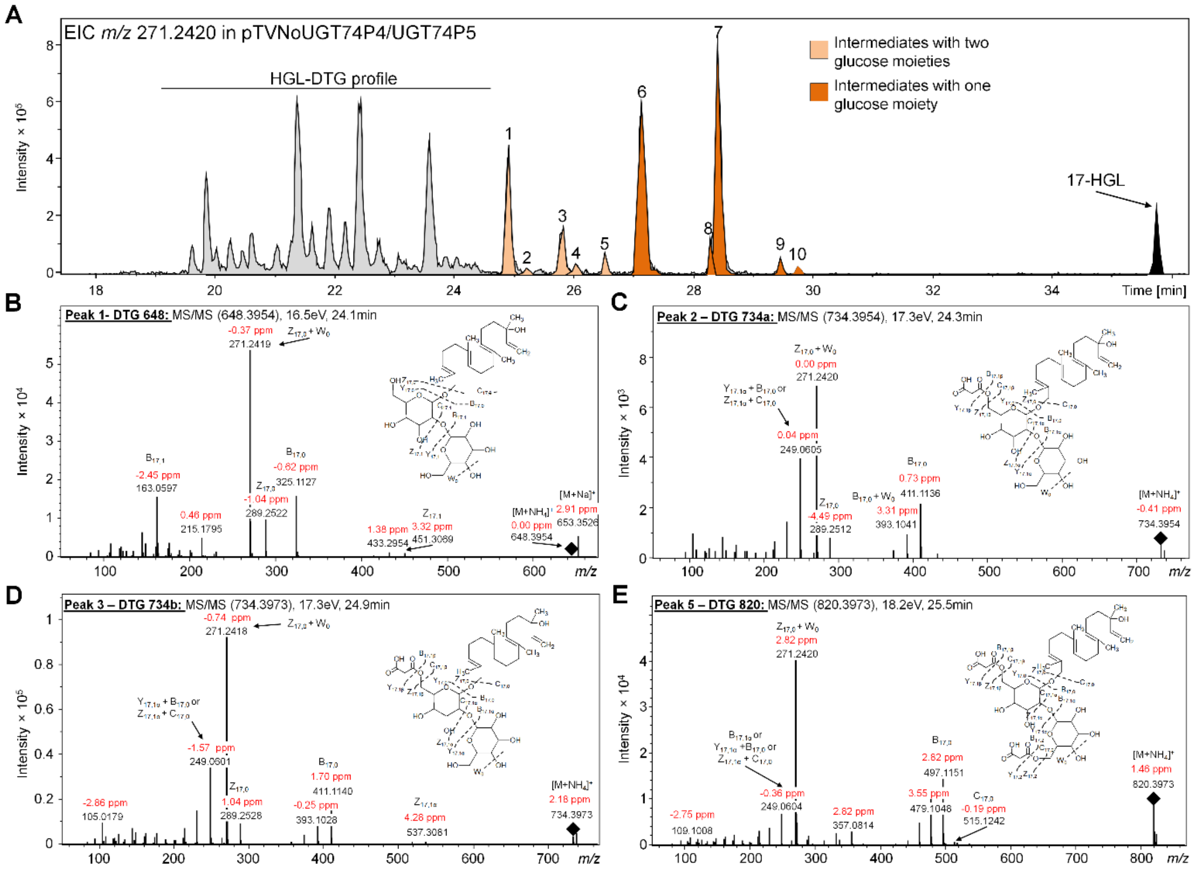

Supplemental Figure 10a: Mass spectrometric characterization and annotation of novel HGL-DTGs in transiently-silenced *N. obtusifolia* plants impaired in No*UGT74P4* and No*UGT74P6* expression. (Supports Figure 3)

A) Shown is the EIC trace *m/z* 271.2420 for the 17-HGL aglycone fragment representing the metabolic alteration of the HGL-DTG profile in transiently-silenced *N. obtusifolia* plants inoculated with *A. tumefaciens* harboring the vector pTVNoUGT74P4/UGT74P6. Additional novel HGL-DTGs are highlighted in light orange for intermediates containing at least two hexoses (presumably glucose) and in darker orange for intermediates containing only one hexose moiety. Furthermore, the associated MS/MS spectra for the highlighted Peak 1 (B), Peak 2 (C), Peak 3 (D) and Peak 5 (E) are displayed. A putative structure is assigned to all intermediate HGL-DTGs and the fragmentation scheme is explained via the predicted neutral losses from the molecular ion $[M+H]^+$.

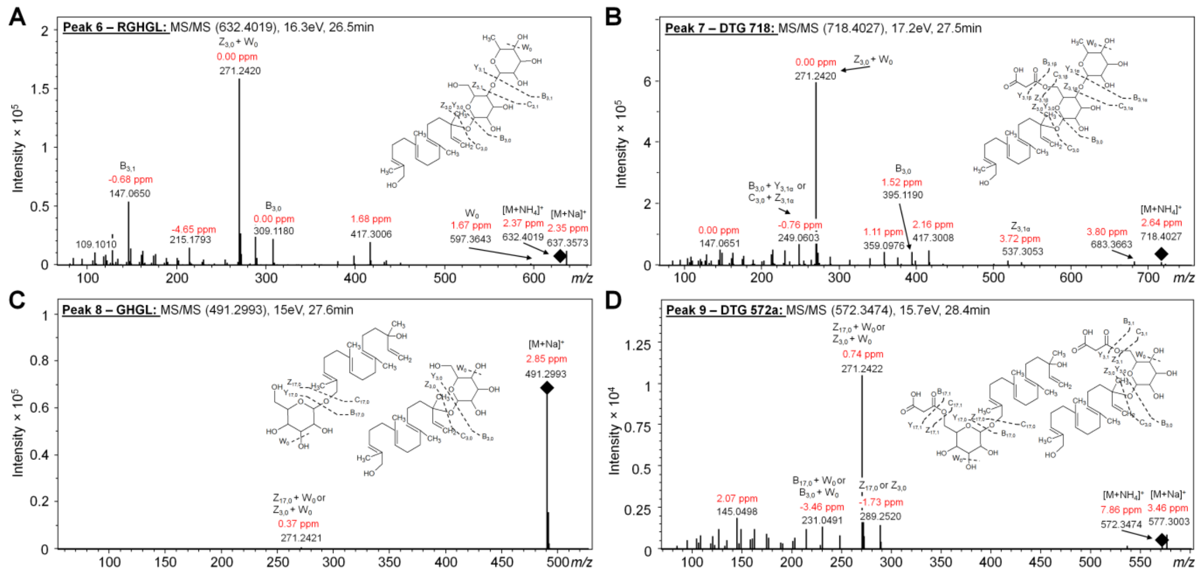

Supplemental Figure 10b: Mass spectrometric characterization and annotation of novel HGL-DTGs in transiently-silenced *N. obtusifolia* plants impaired in No*UGT74P4* and No*UGT74P6* expression. (Supports Figure 3)

MS/MS spectra for the highlighted Peak 6 (A), Peak 7 (B), Peak 8 (C) and Peak 9 (D) are displayed. A putative structure is assigned to all intermediate HGL-DTGs and the fragmentation scheme is explained via the predicted neutral losses from the molecular ion $[M+H]^+$.

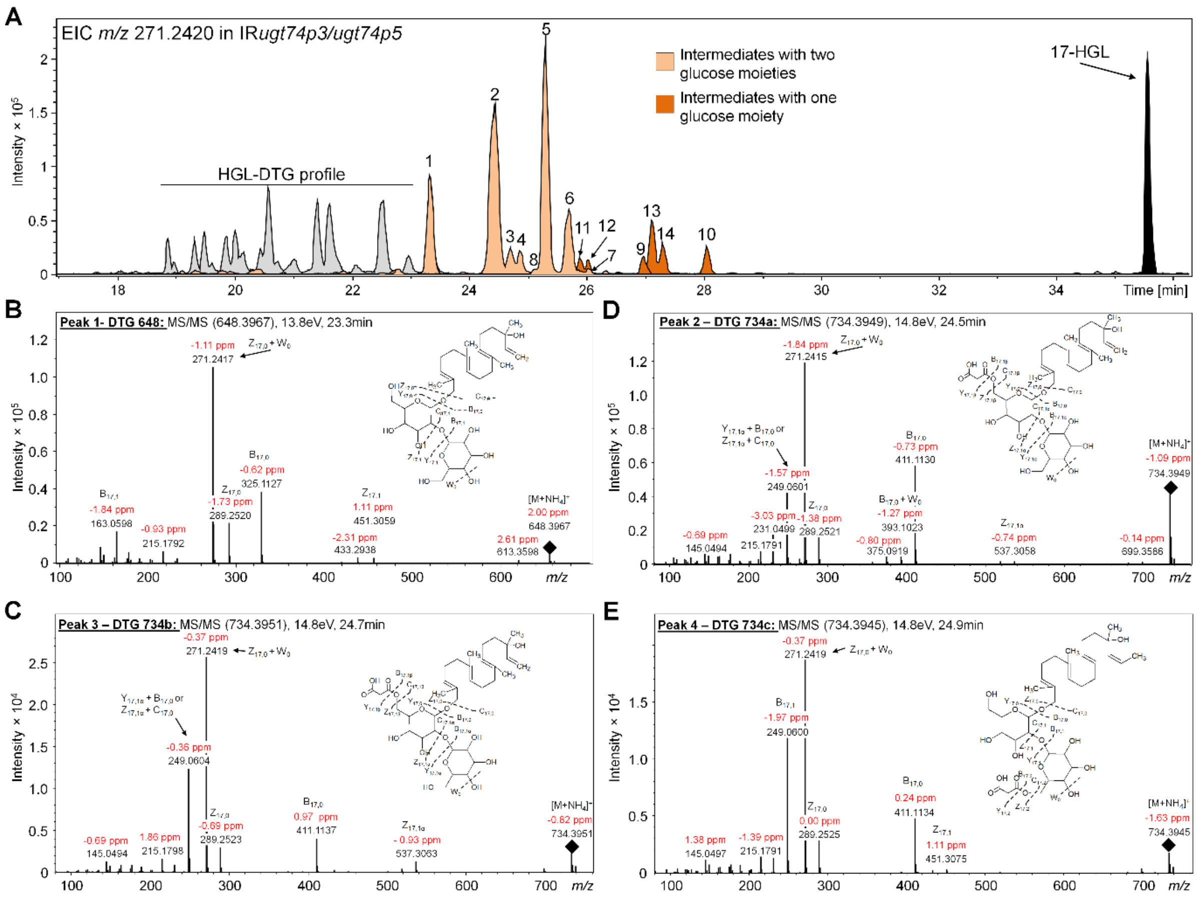

Supplemental Figure 11a: Characterization and annotation of novel HGL-DTGs via MS/MS in stably-silenced *N. attenuata* plants impaired in *UGT74P3* and *UGT74P5* expression. (Supports Figure 2, 4 and 5)

A) Shown is the EIC trace *m/z* 271.2420 for the 17-HGL aglycone fragment representing the metabolic alteration of the HGL-DTG profile in stably-silenced *N. attenuata* plants impaired in *UGT74P3* and *UGT74P5* expression. Additional novel HGL-DTGs are highlighted in light orange for intermediates containing at least two hexoses (presumably glucose) and in darker orange for intermediates containing only one hexose moiety. Furthermore, the associated MS/MS spectra for the highlighted Peak 1 (B), Peak 2 (D), Peak 3 (C) and Peak 4 (E) are displayed. A putative structure is assigned to all intermediate HGL-DTGs and the fragmentation scheme is explained via the predicted neutral losses from the molecular ion $[M+H]^+$.

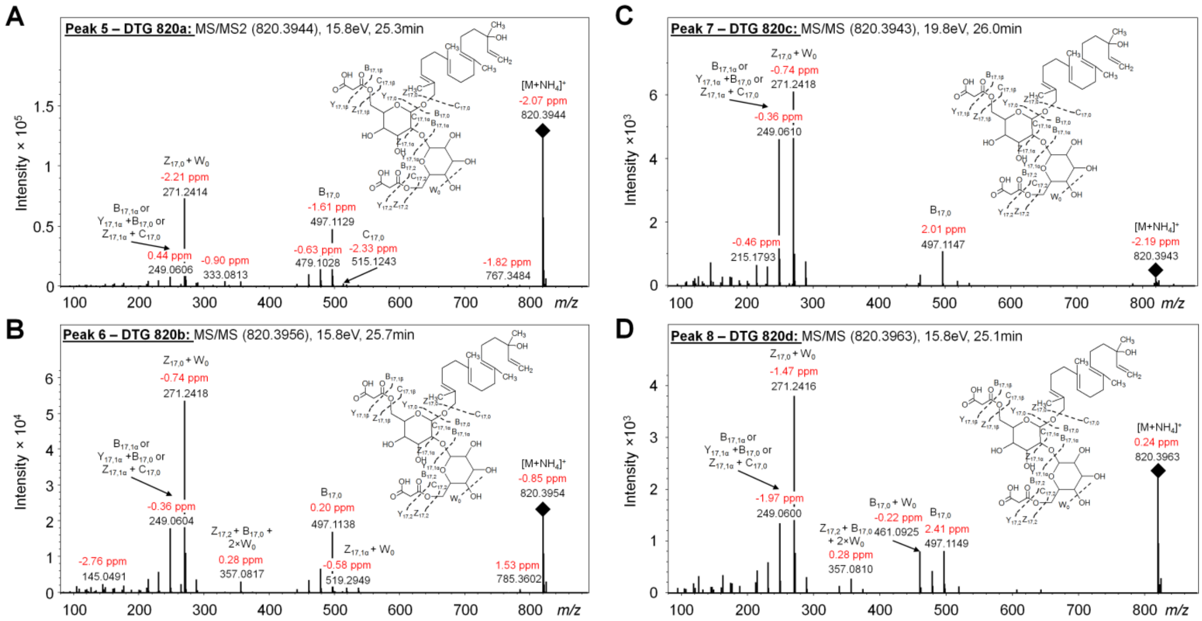

Supplemental Figure 11b: Characterization and annotation of novel HGL-DTGs via MS/MS in stably-silenced *N. attenuata* plants impaired in *UGT74P3* and *UGT74P5* expression. (Supports Figures 2, 4 and 5)

MS/MS spectra for the highlighted Peak 5 (A), Peak 6 (B), Peak 7 (C) and Peak 8 (D) are displayed. A putative structure is assigned to all intermediate HGL-DTGs and the fragmentation scheme is explained via the predicted neutral losses from the molecular ion $[M+H]^+$.

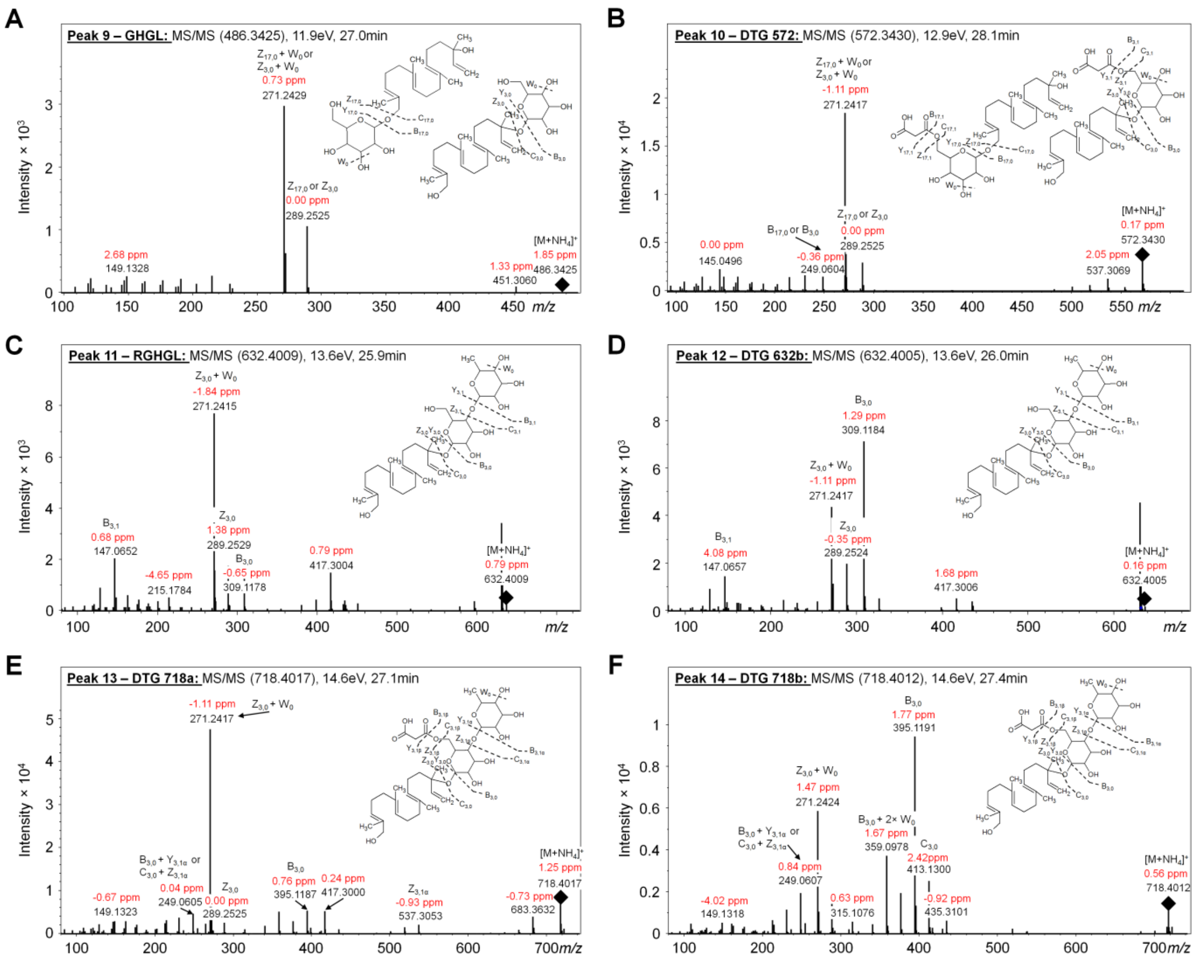

Supplemental Figure 11c: Characterization and annotation of novel HGL-DTGs via MS/MS in transiently-silenced *N. attenuata* plants impaired in *UGT74P3* and *UGT74P5* expression. (Supports Figures 2, 4 and 5)

MS/MS spectra for the highlighted Peak 9 (A), Peak 10 (B), Peak 11 (C), Peak 12 (D), Peak 13 (E) and Peak 14 (F) are displayed. A putative structure is assigned to all intermediate HGL-DTGs and the fragmentation scheme is explained via the predicted neutral losses from the molecular ion $[M+H]^+$.

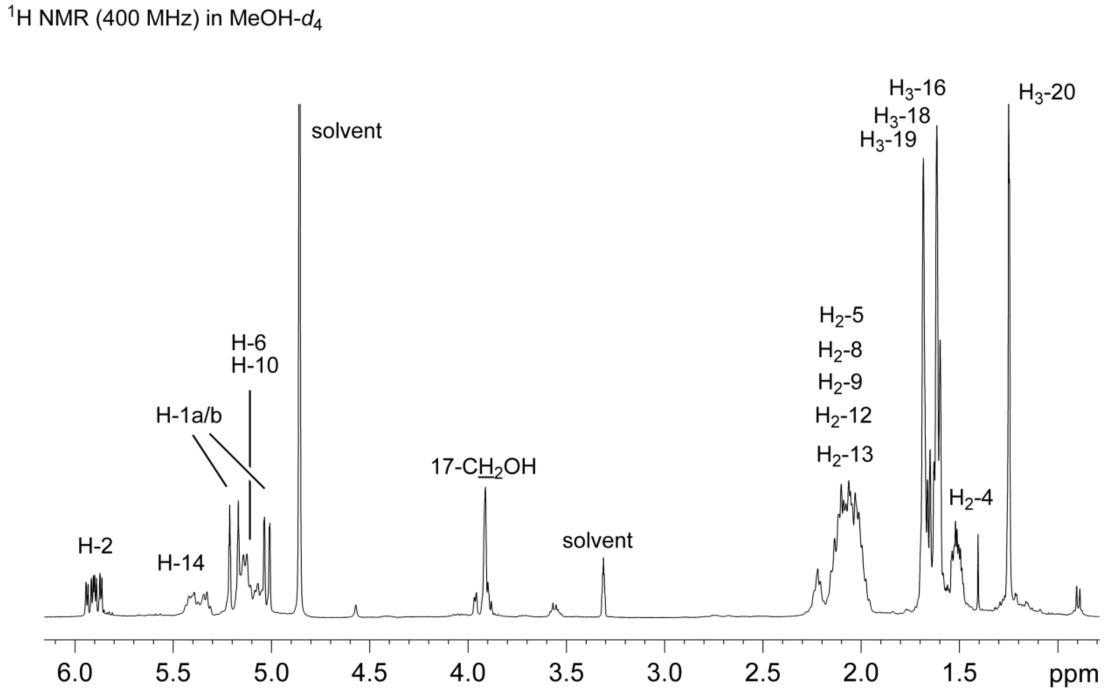

Supplemental Figure 12: ^{1}H NMR spectrum of synthetic 17-hydroxygeranyllinalool (17-HGL, HPC24 Standards) **(Supports Figures 2, 4, 5 and 8)**

17-HGL was measured at 400 MHz in MeOH-d_4 (300 K). Signals were assigned using data from DEPT 135, ^{1}H-^{1}H COSY, HSQC and HMBC spectra.

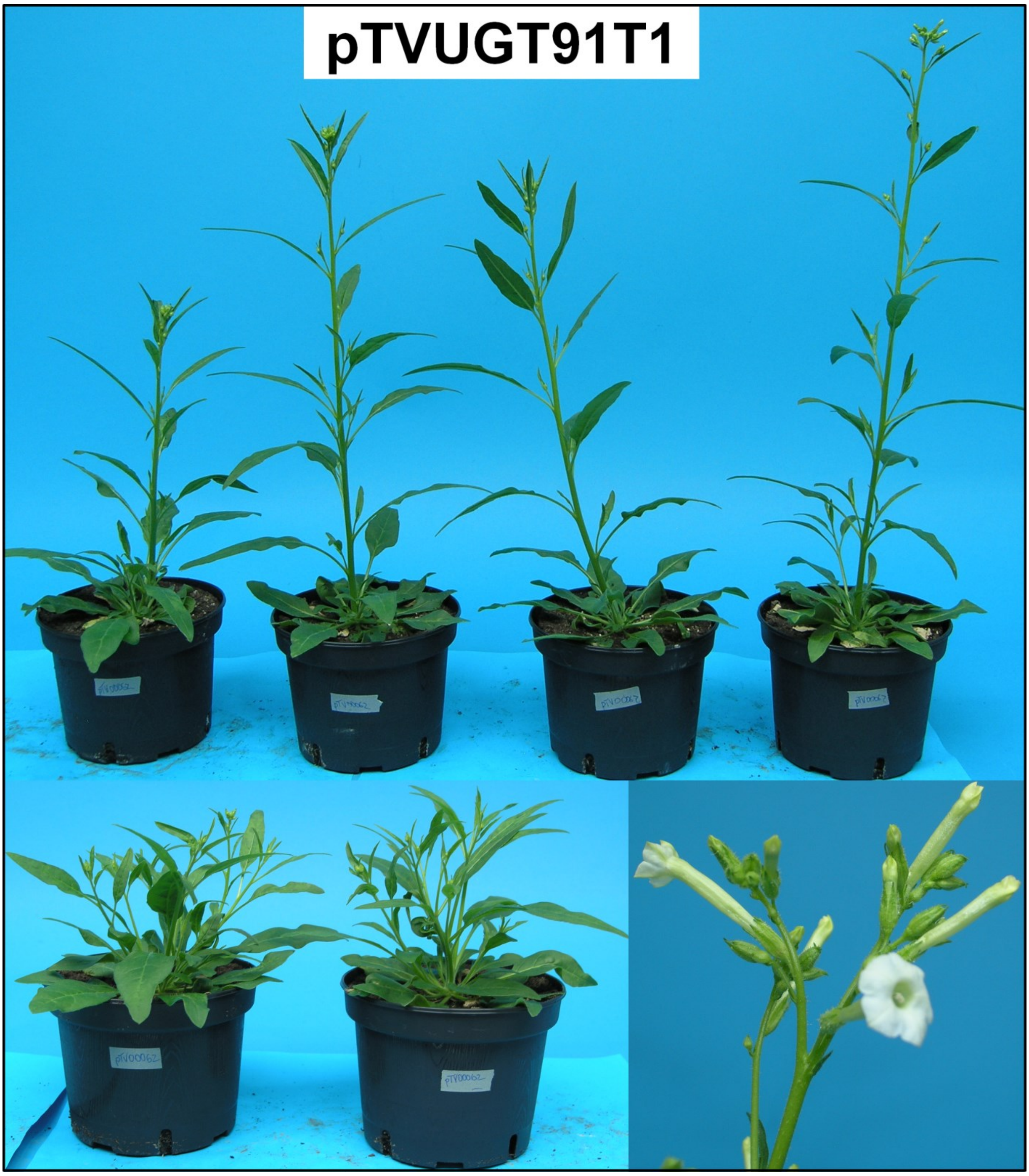

Supplemental Figure 13a: Morphological characterization of *N. attenuata* plants transiently-silenced in *UGT91T1* expression (Supports Figure 2)

Shown are 37-day-old elongated *N. attenuata* plants transiently-silenced via VIGS in *UGT91T1* expression.

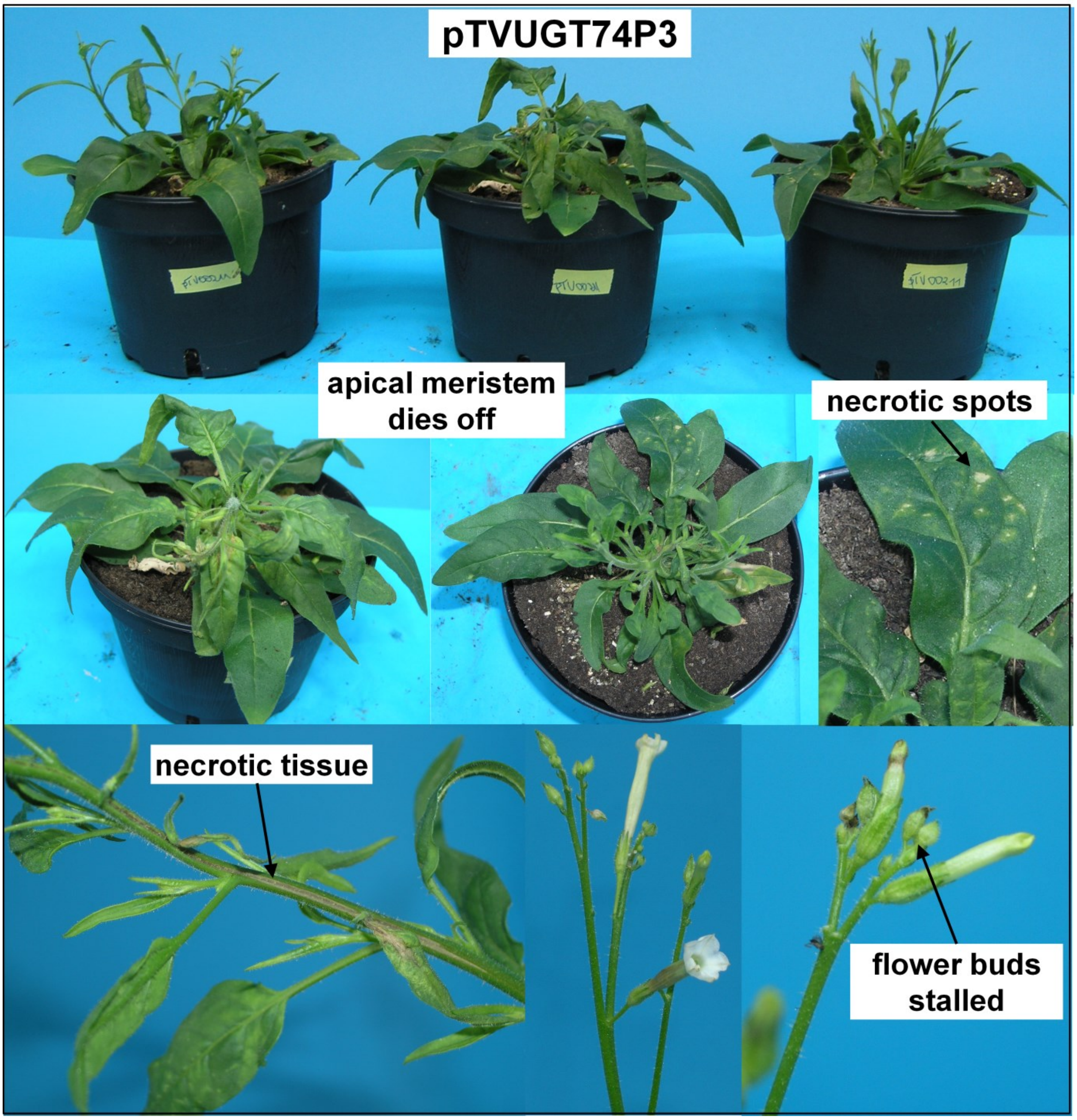

Supplemental Figure 13b: Morphological characterization of *N. attenuata* plants transiently-silenced in *UGT74P3* expression (Supports Figures 2)

Shown are 37-day-old elongated *N. attenuata* plants transiently-silenced via VIGS in *UGT74P3* expression. Morphological alterations that ranged from the presence of necrotic spots and tissues to dead apical meristems and a high percentage of stalled flower buds.

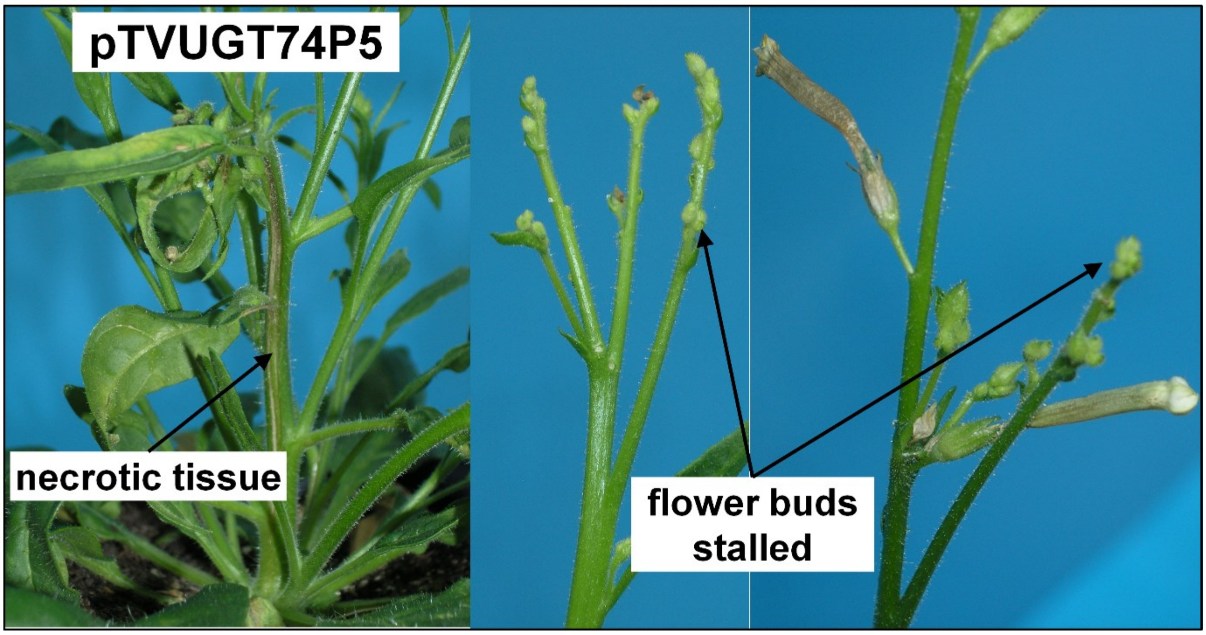

Supplemental Figure 13c: Morphological characterization of *N. attenuata* plants transiently-silenced in *UGT74P5* expression (Supports Figures 2)

Shown are 37-day-old elongated *N. attenuata* plants transiently-silenced via VIGS in *UGT74P5* expression. Morphological alterations like necrotic tissues and a high percentage of stalled flower buds were observed.

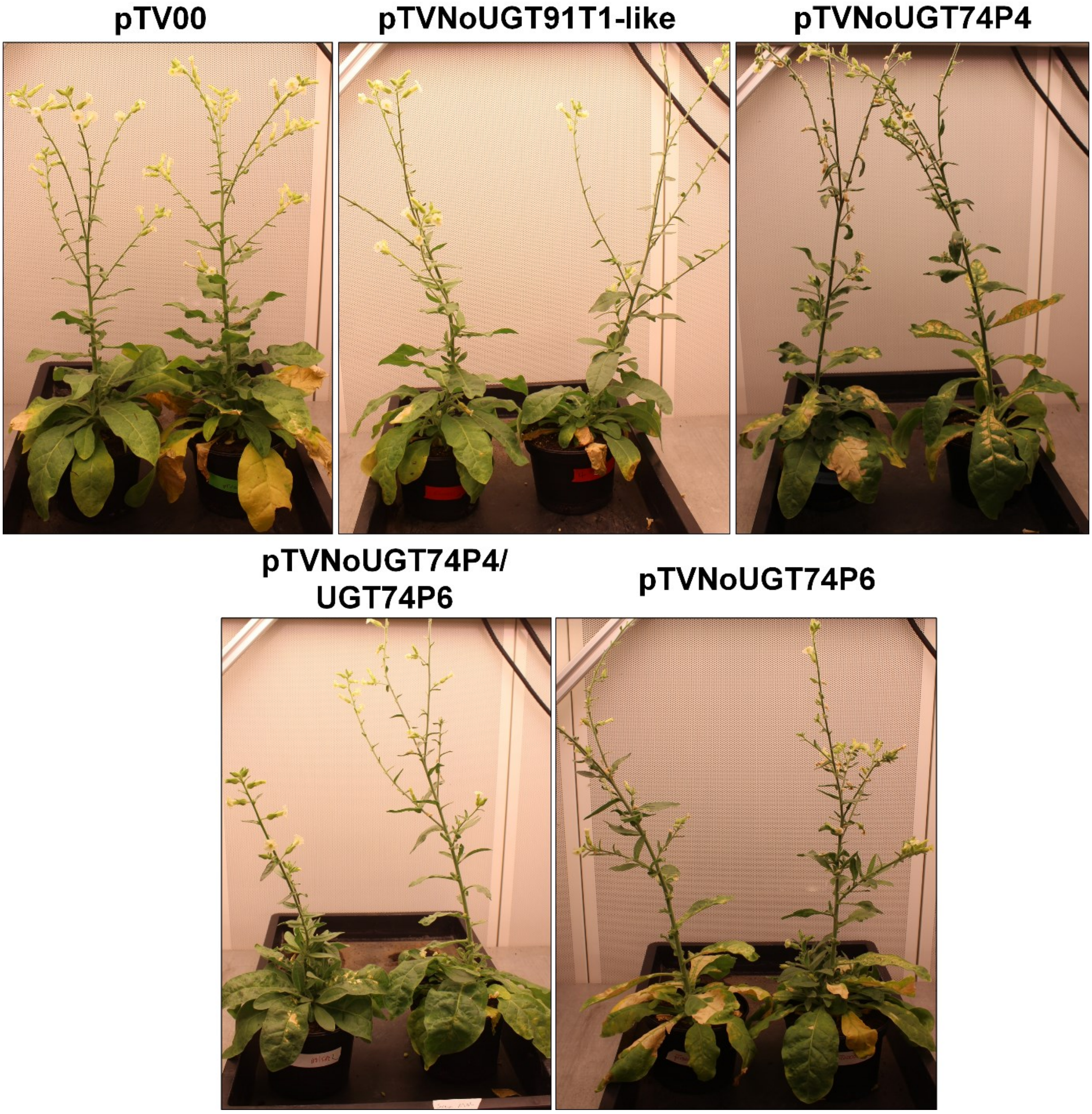

Supplemental Figure 14: Morphological characterization of *N. obtusifolia* plants transiently-silenced in No*UGT91T1-like*, No*UGT74P4*, No*UGT74P6* and No*UGT74P4*/*UGT74P6* expression. (Supports Figures 3)

Shown are 60-day-old flowering *N. obtusifolia* plants transiently-silenced via VIGS in No*UGT91T1-like*, No*UGT74P4*, No*UGT74P6* and No*UGT74P4*/*UGT74P6* expression. Withered flowers were removed. Abundant necrotic spots were only detected in pTVNoUGT74P4 and pTVNoUGT74P6. In contrast to *N. attenuata,* no apical meristem necrosis could be detected.

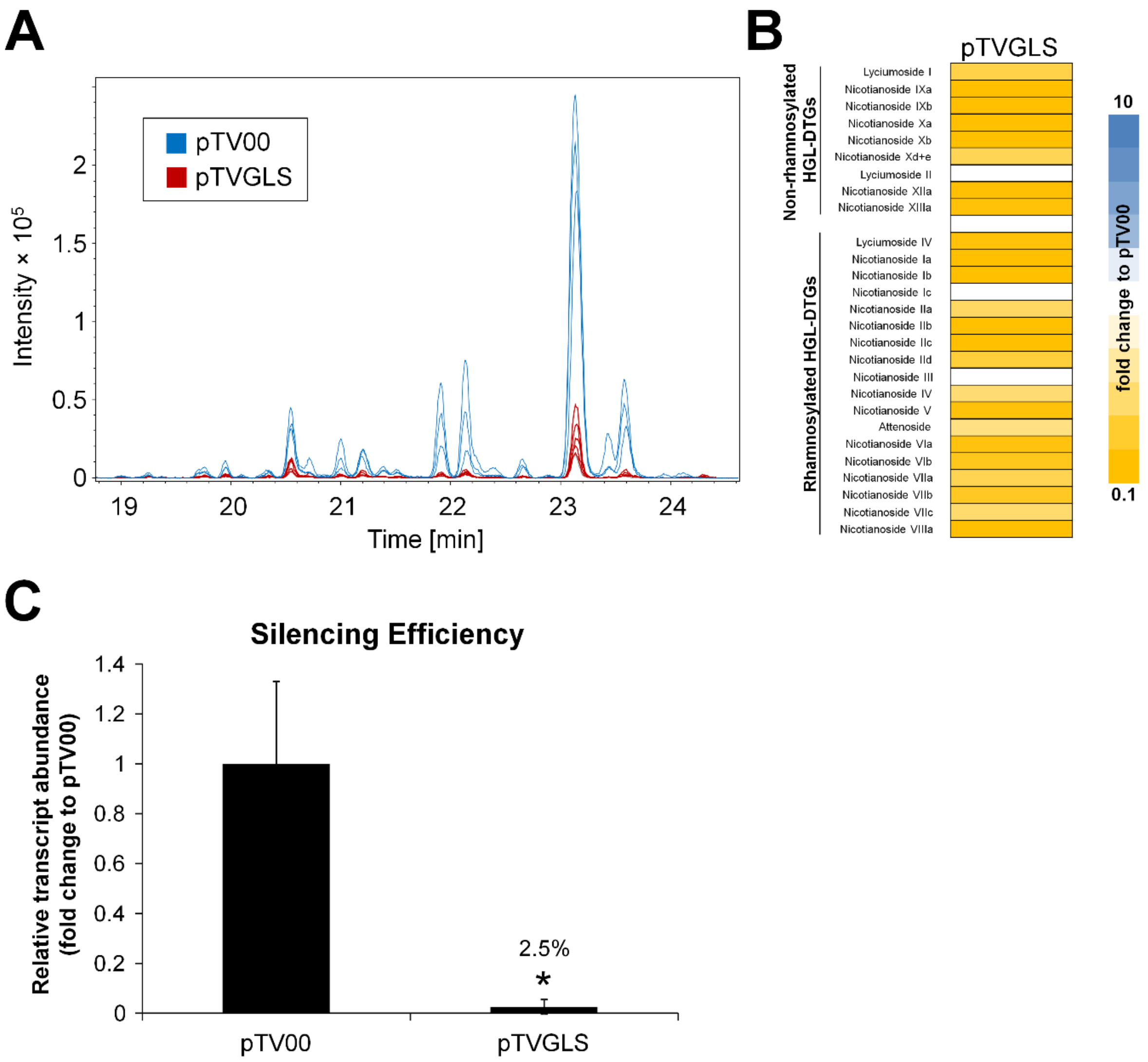

Supplemental Figure 15: Metabolite profiling and morphological characterization of *N. attenuata* plants transiently-silenced via virus-induced gene silencing (VIGS) of geranyllinalool synthase (GLS) (Supports Figure 2 and 5)

A) Shown is the EIC trace for the HGL-DTG aglycone fragment (*m/z* 271.2420) in 37 days old elongated transiently-silenced *N. attenuata* plants impaired in *GLS* expression as well as empty vector control plants (pTV00). B) Heatmap visualization of deregulations of the leaf HGL-DTG profile of pTVGLS transformed plants (N=4). The color gradient visualizes fold changes in individual HGL-DTGs for the GLS-silenced plants compared to the average in the pTV00 empty vector plants. Further details on the abundance of HGL-DTGs can be found in Supplemental Data Set 3 - GLS. C) Relative transcript abundance (fold change to *N. attenuata Elongation Factor 1α* – Na*ELF1α*) of Na*GLS* in leaves of transiently-silenced *N. attenuata* plants (average ± SE; N=4). Asterisks indicate significant differences between pTV00 empty vector control and pTVGLS (*t*-test, *P ≤ 0.05).

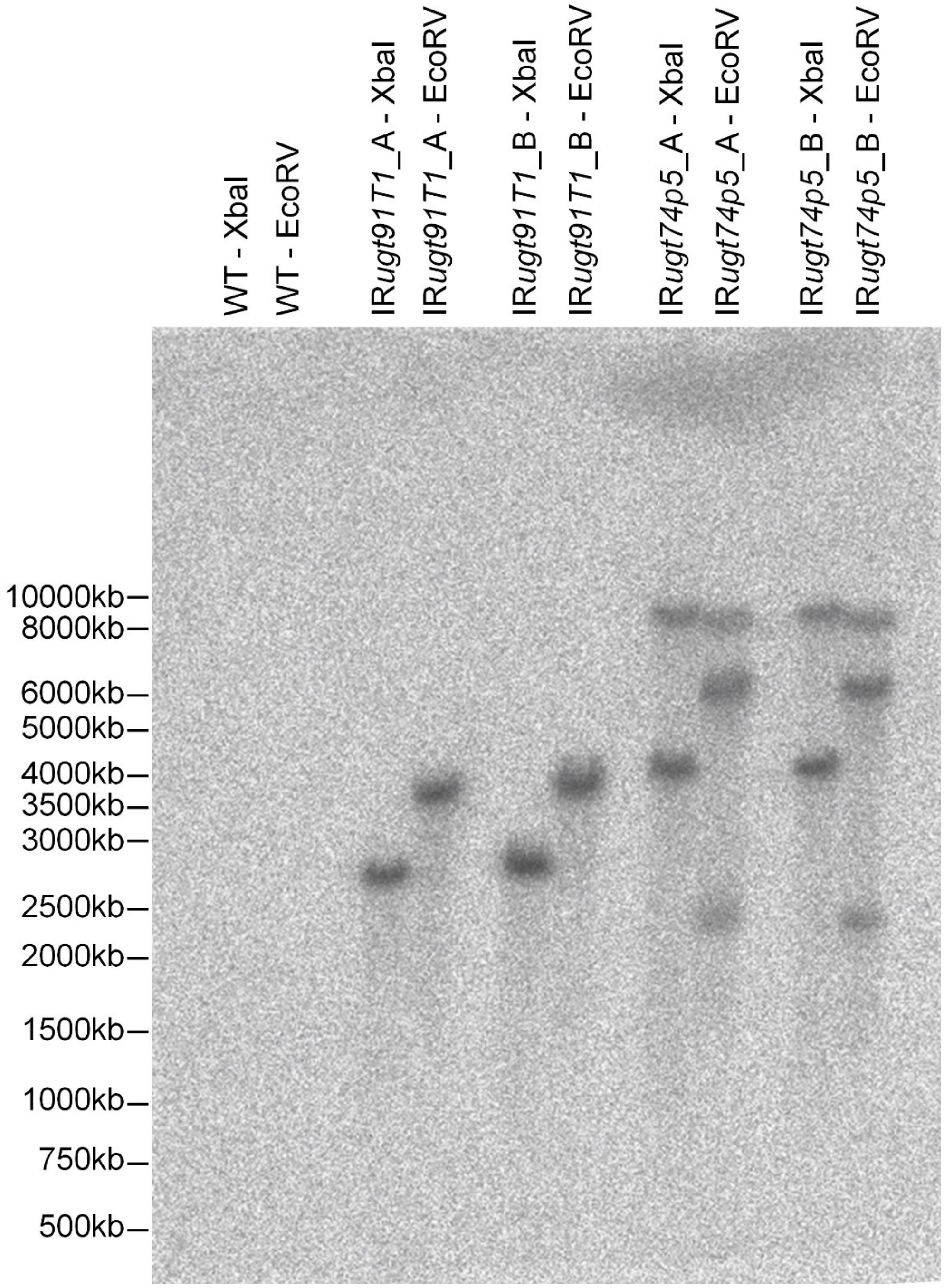

Supplemental Figure 16: DNA gel blot analysis (Supports Figure 5)

Examination of the insertion events of both IR*ugt91t1* and both IR*ugt74p5* transformed lines using XbaI and EcoRV as restriction enzymes.

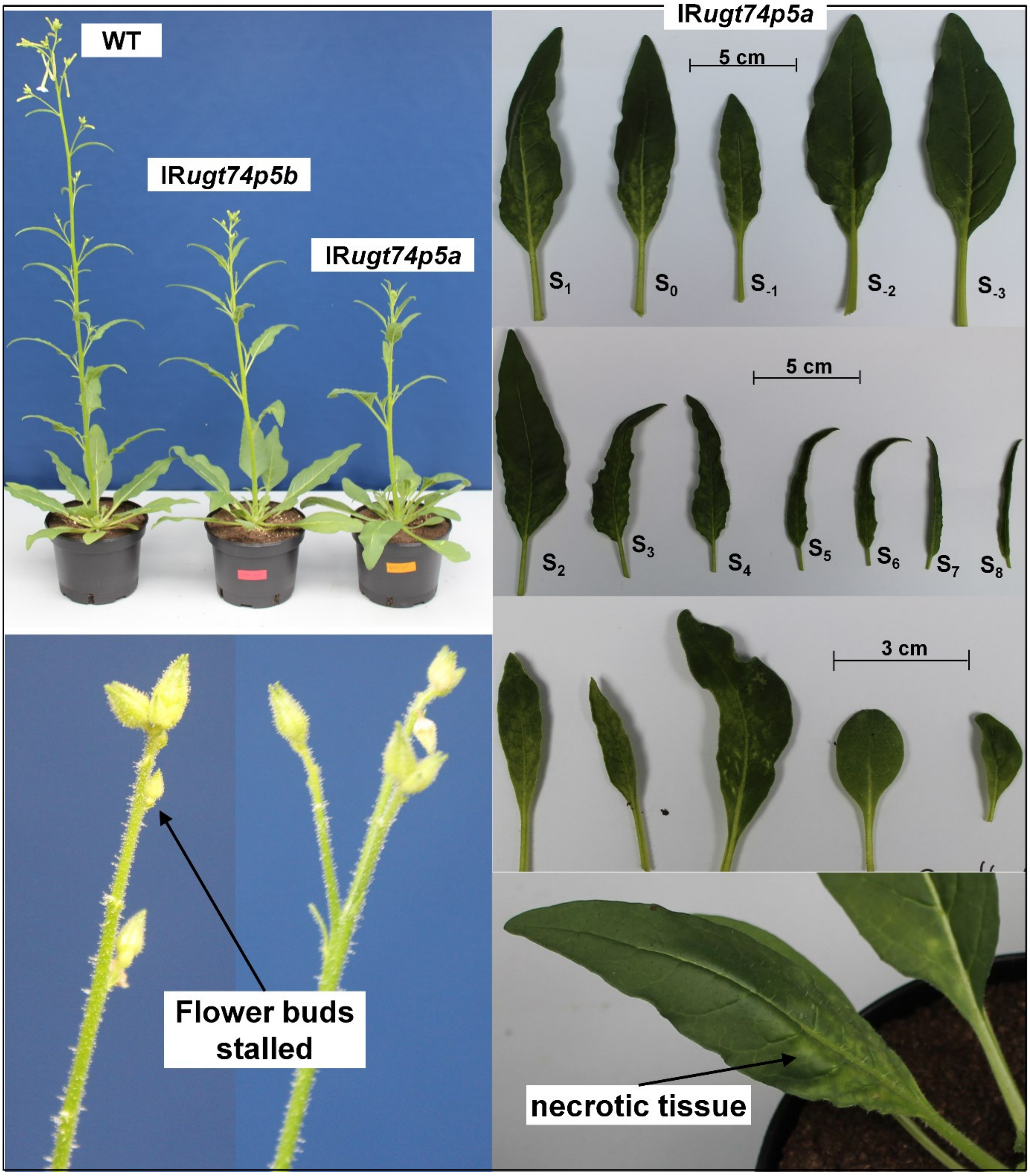

Supplemental Figure 17a: Morphological characterization of the stably transformed *IRugt74p5* Line A (Supports Figure 5)

Shown are 43-day-old flowering *N. attenuata* plants impaired in *UGT74P5* expression compared to wild type. Morphological alterations of IR*ugt74p5a* ranged from curly deformed rosette leaves with necrotic spots to deformed thin stem leaves. Additionally, several “dwarfish” or succulent round shaped leaves and chlorotic stalled flower buds were observed.

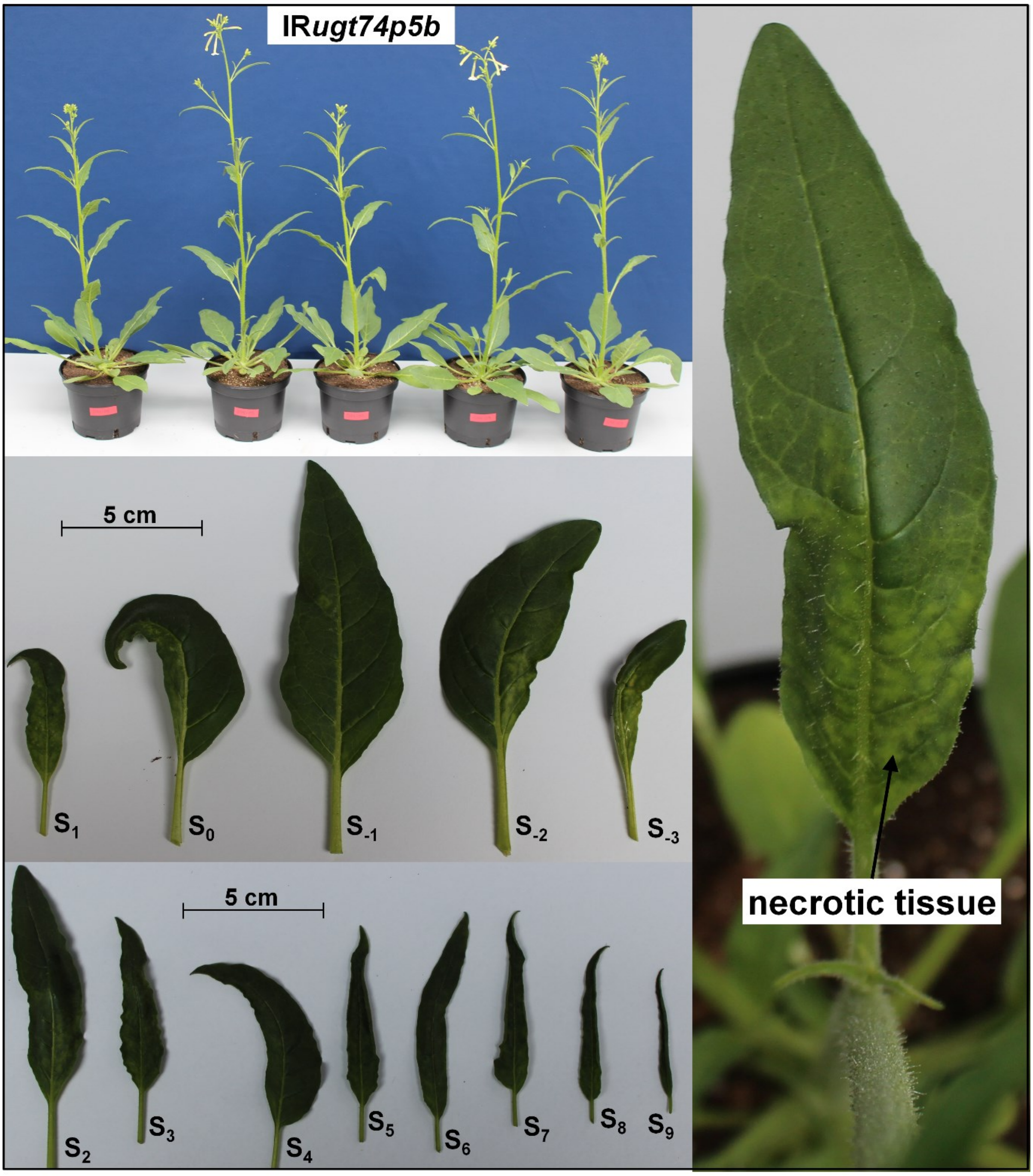

Supplemental Figure 17b: Morphological characterization of the stably transformed *IRugt74p5* Line B (Supports Figure 5)

Shown are 43-day-old flowering *N. attenuata* plants impaired in *UGT74P5* expression (Line B). Morphological alterations of IR*ugt74p5b* ranged from curly deformed thin and necrotic rosette leaves to deformed thin rippled stem leaves.

IR*ugt74p3/ugt74p5*

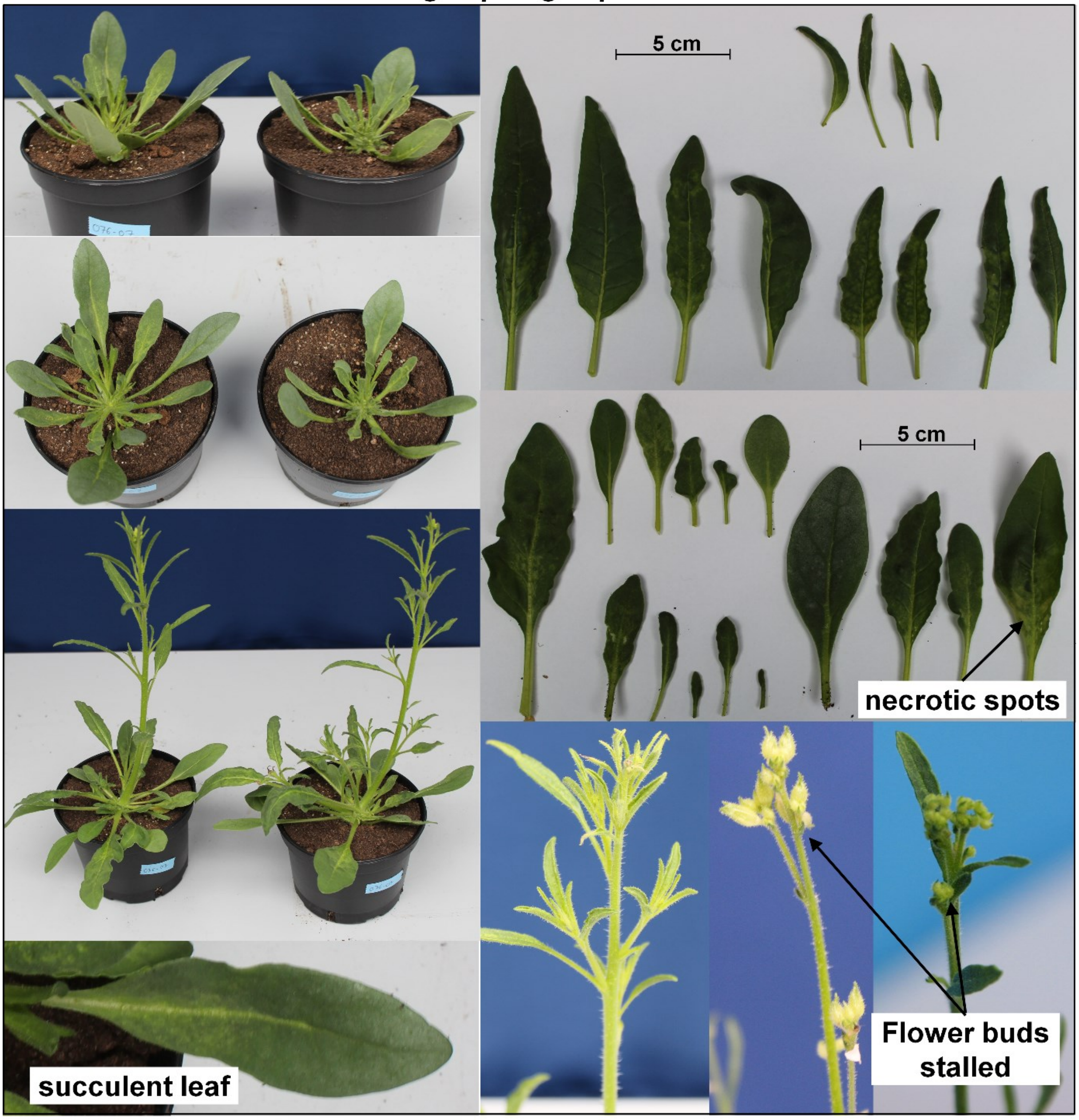

Supplemental Figure 17c: Morphological characterization of the stably transformed *IRugt74p3/ugt74p5*. (Supports Figure 5)

Shown are 43-day-old flowering *N. attenuata* plants impaired in UGT74P3 and *UGT74P5* expression. Morphological alterations of IR*ugt74p3/ugt74p5* ranged from curly, thin and deformed rosette leaves with necrotic spots to "dwarfish" or succulent round-shaped stem leaves with a high grade of deformation. Furthermore, higher branching grades as well as stalled chlorotic flower buds were observed. Additionally several impaired plants displayed a "dwarf-like" phenotype with small deformed and succulent leaves.

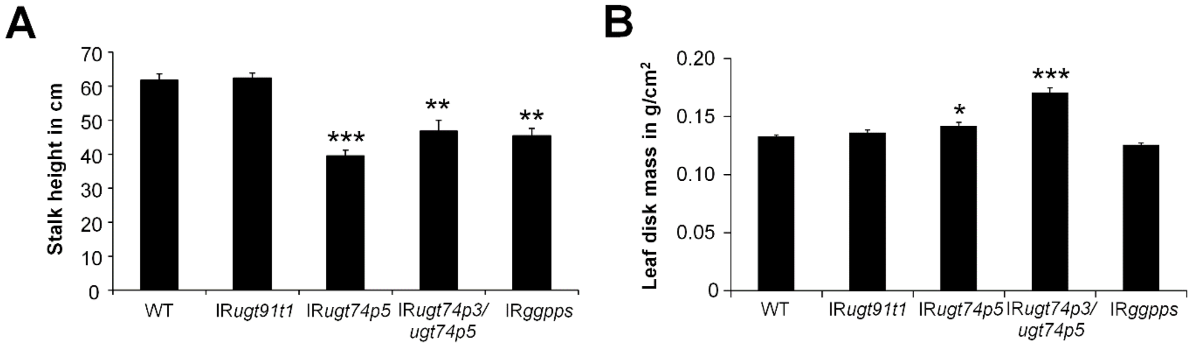

Supplemental Figure 18: Characterization of growth parameters in IR*ugt91t1*, IR*ugt74p5*, IR*ugt74p3/ugt74p5* and IR*ggpps* **(Supports Figure 5)**

Displayed is the stalk height (A) and leaf disk mass (B) of 40-day-old elongated *N. attenuata* plants stably transformed with IR*ugt91t1* Line A, IR*ugt74p5* Line B, IR*ugt74p3/ugt74p5*, IR*ggpps* at the transition to the flowering stage (average ± SE; N=15). Asterisks indicate significant differences between wild type control and stably-silenced lines (*P≤ 0.05, ** P < 0.01, *** P < 0.001).

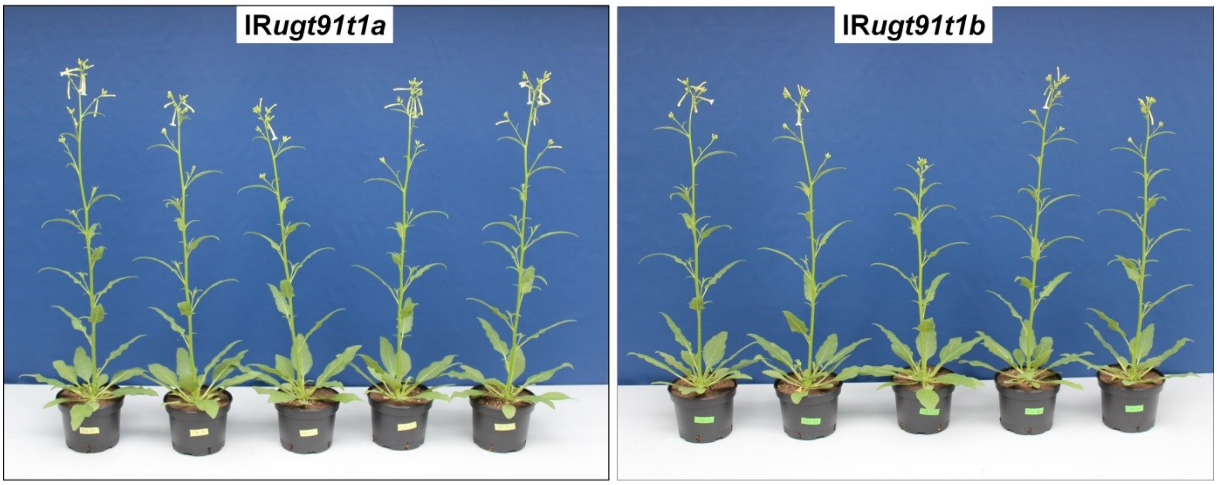

Supplemental Figure 19: Morphological characterization of IR*ugt91t1* (Supports Figure 5)

Shown are 43-day-old *N. attenuata* plants of both stably silenced IR*ugt91t1* lines.

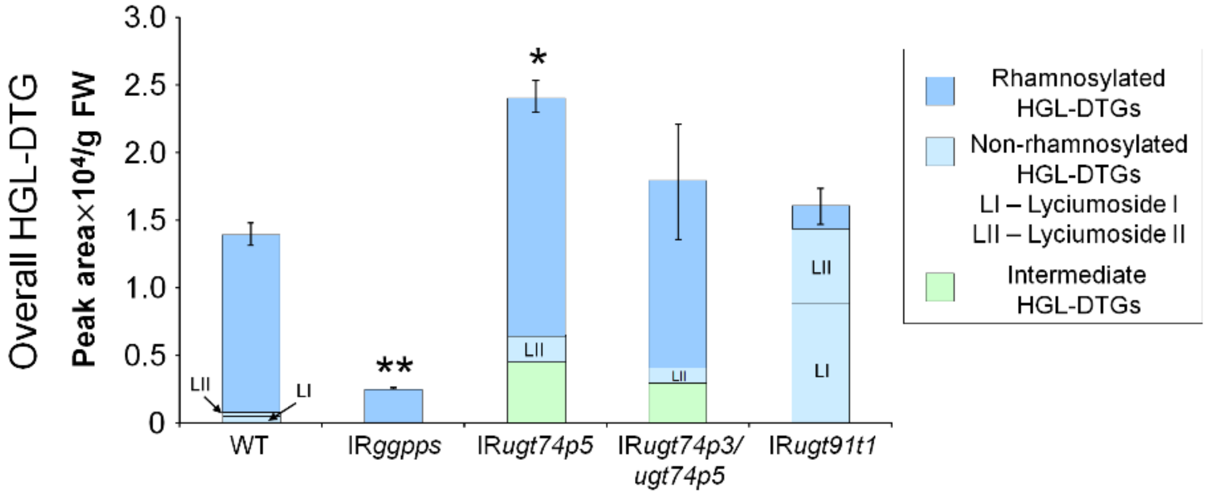

Supplemental Figure 20: Overall abundance of HGL-DTGs (Supports Figure 5)

Shown is the overall area/g FW of rhamnosylated, non-rhamnosylated and intermediate HGL-DTGs in the different stable lines (average ± SE; N=5). LI and LII represent the most abundant non-rhamnosylated HGL-DTGs lyciumoside I and lyciumoside II and their malonylated forms in the different lines. Asterisks indicate significant differences between WT and stably-silenced lines (*P ≤ 0.05, ** P < 0.01).

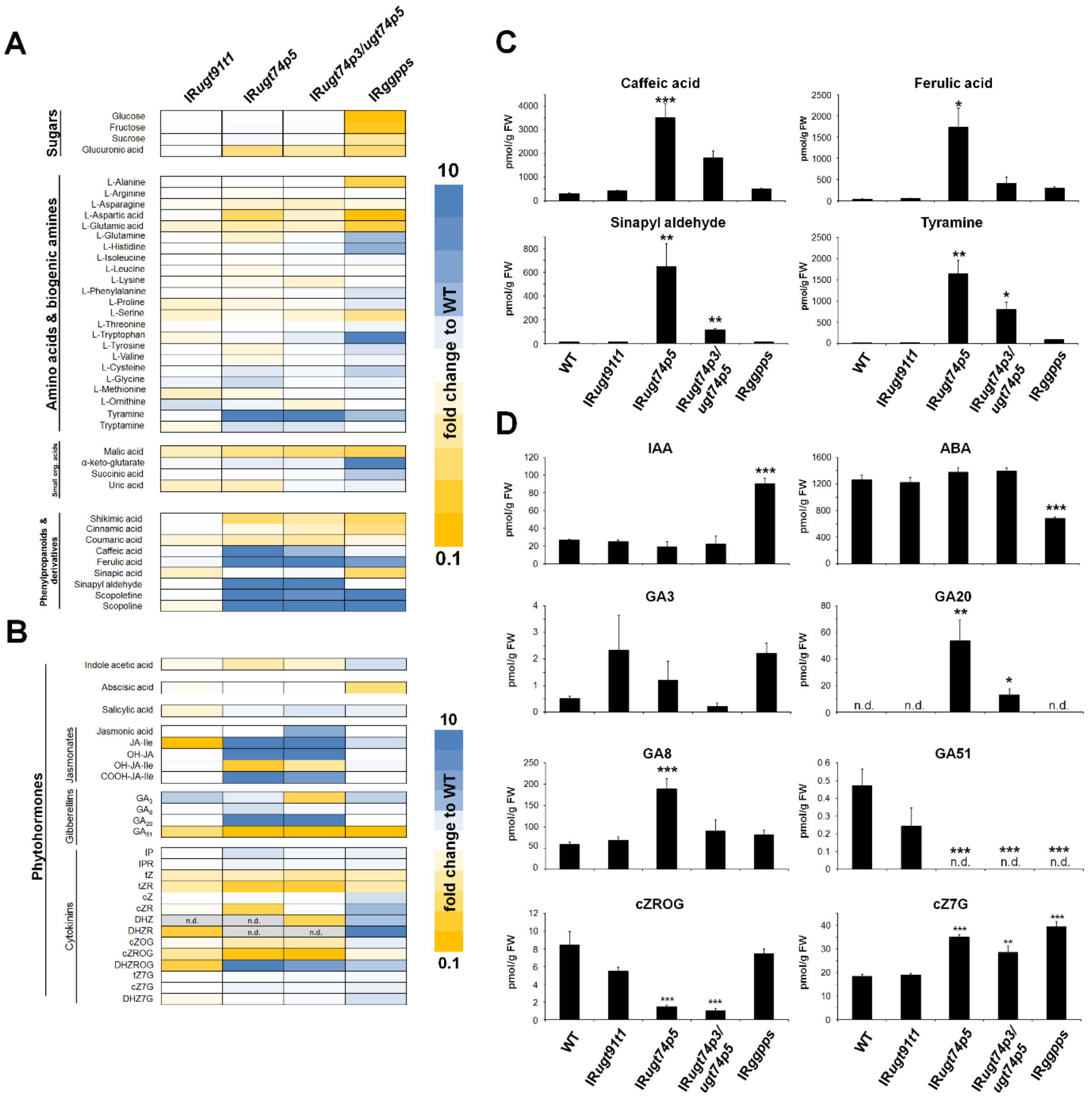

Supplemental Figure 21: Disrupting HGL-DTG glycosylation reorganizes general, specialized and hormonal metabolic pathways (Supports Figure 5)

Heatmap visualization of deregulations in the leaves' A) + C) general/specialized and B) + D) hormonal metabolic profiles of IR*ugt91t1*, IR*ugt74p5*, IR*ugt74p3/ugt74p5* and IR*ggpps* plants (average ± SE; N=5). Color gradients visualize fold changes in individual metabolites for each of the stable lines compared to the average in the WT plants. Asterisks indicate significant differences between WT control and stable transformants (*P ≤ 0.05, ** P < 0.01, *** P < 0.001).

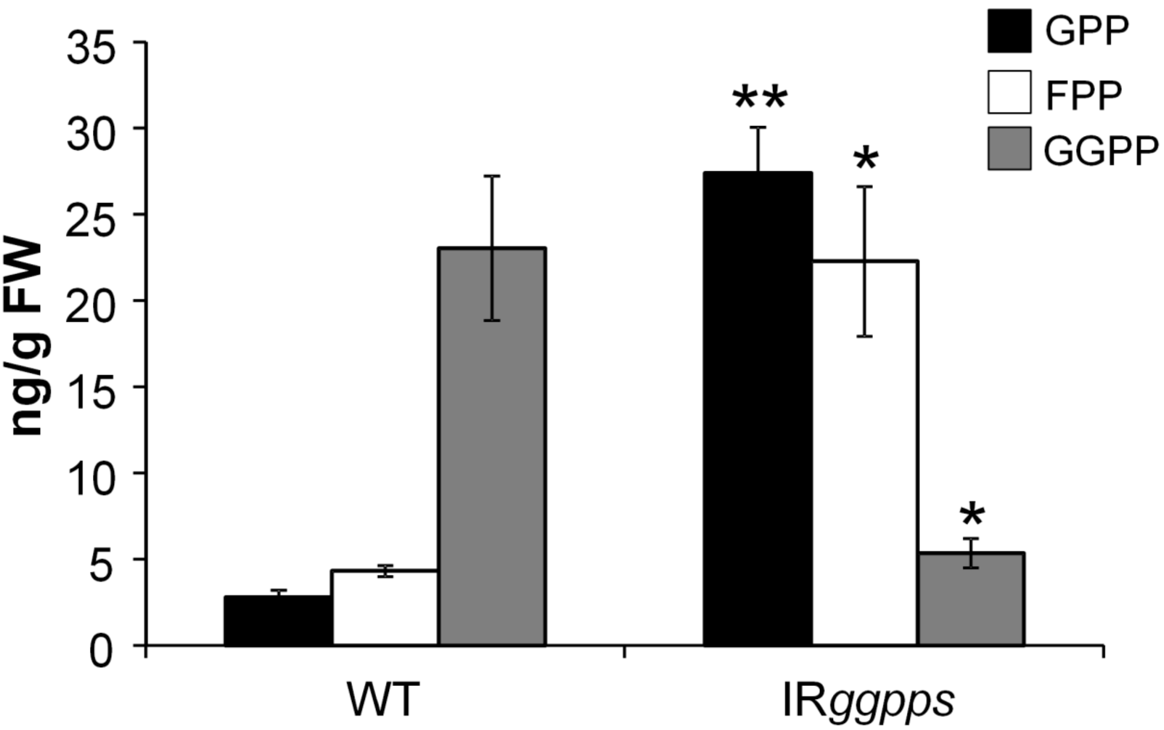

Supplemental Figure 22: Characterization of free prenyldiphosphates in IR*ggpps* and WT (Supports Figure 5)

Shown is the amount of the free prenyldiphosphates, GDP, FDP and GGDP, in leaf tissue of IR*ggpps* and wild type *N. attenuata* plants (average ± SE; N=4). Asterisks indicate significant differences between WT and IR*ggpps* (*t*-test, *P ≤ 0.05, ** P < 0.01).

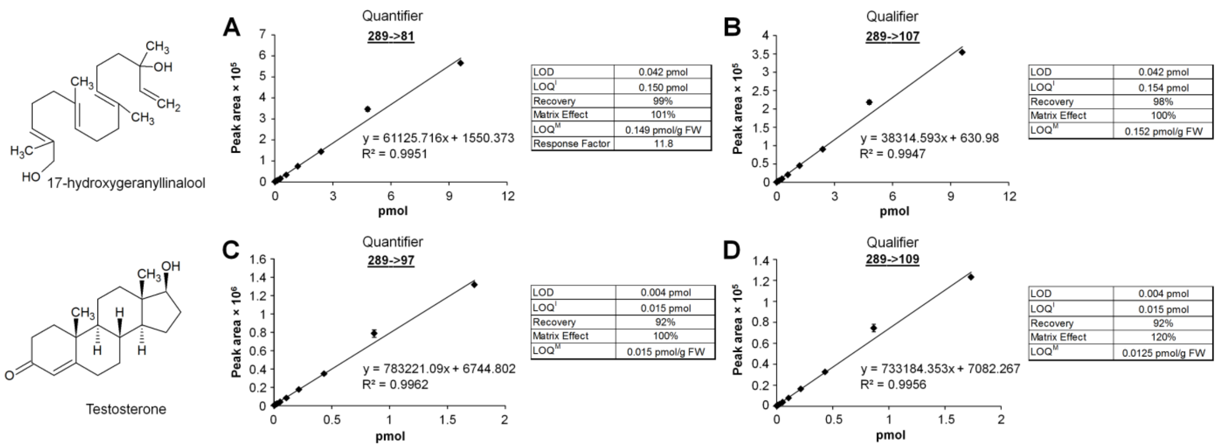

Supplemental Figure 23: Quantitative 17-HGL method (Supports Figure 8)

Shown are the standard curves for the quantifiers (A and C) and qualifiers (B and D) for 17-HGL as well as the testosterone internal standard. Method parameters like limit of detection (LOD), limit of quantification (LOQ), recovery, matrix effect, quantification limit of the instrument (LOQ^M) and the response factor are presented.

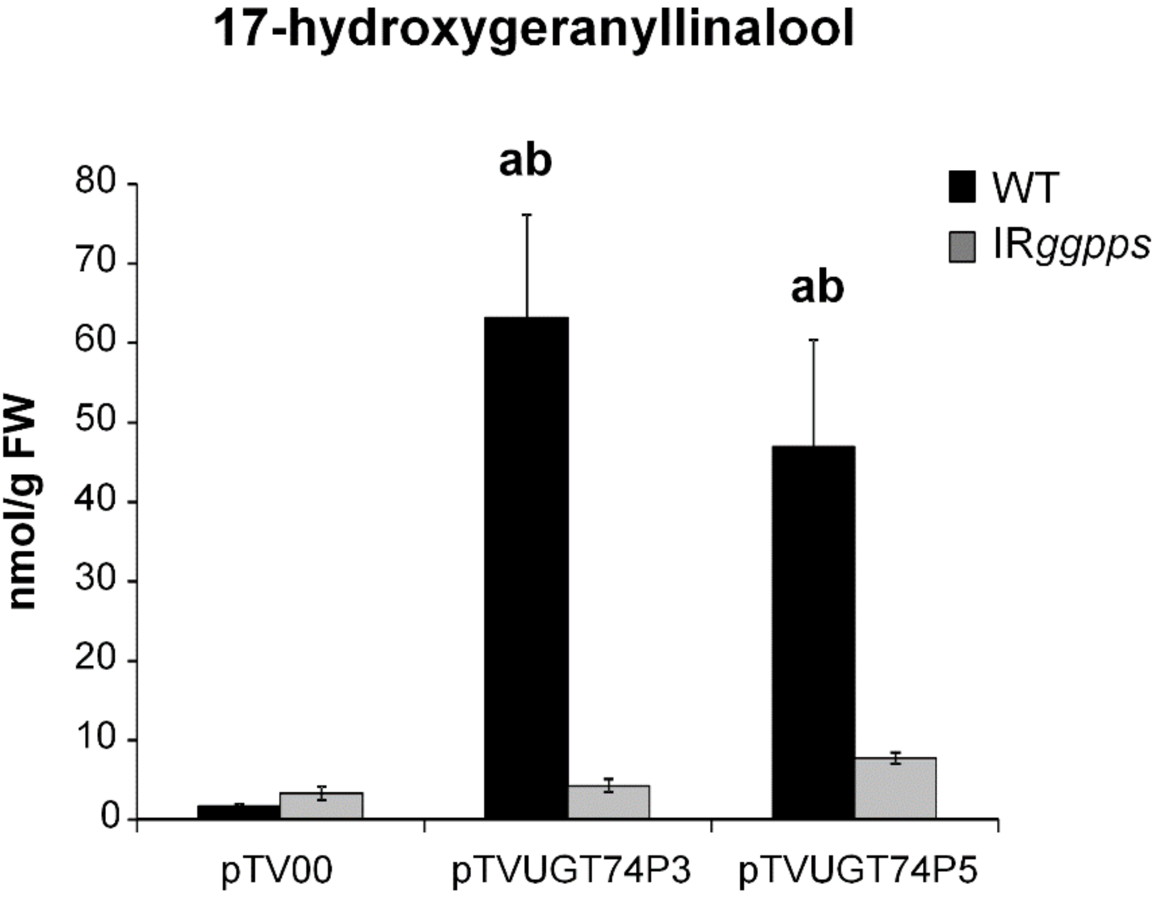

Supplemental Figure 24: 17-HGL concentration in IR*ggpps* and WT plants transiently transformed with pTV00, pTVUGT74P3 and pTVUGT74P5 (Supports Figure 7)

Shown is the amount of the 17-HGL in leaf tissue of WT and IR*ggpps* plants transiently transformed with pTV00, pTVUGT74P3 and pTVUGT74P5 (average ± SE; N=5). Letters indicate significant differences from the pTV00 empty vector control (a) and between IR*ggpps* and wild type (b) ($P \leq 0.05$).

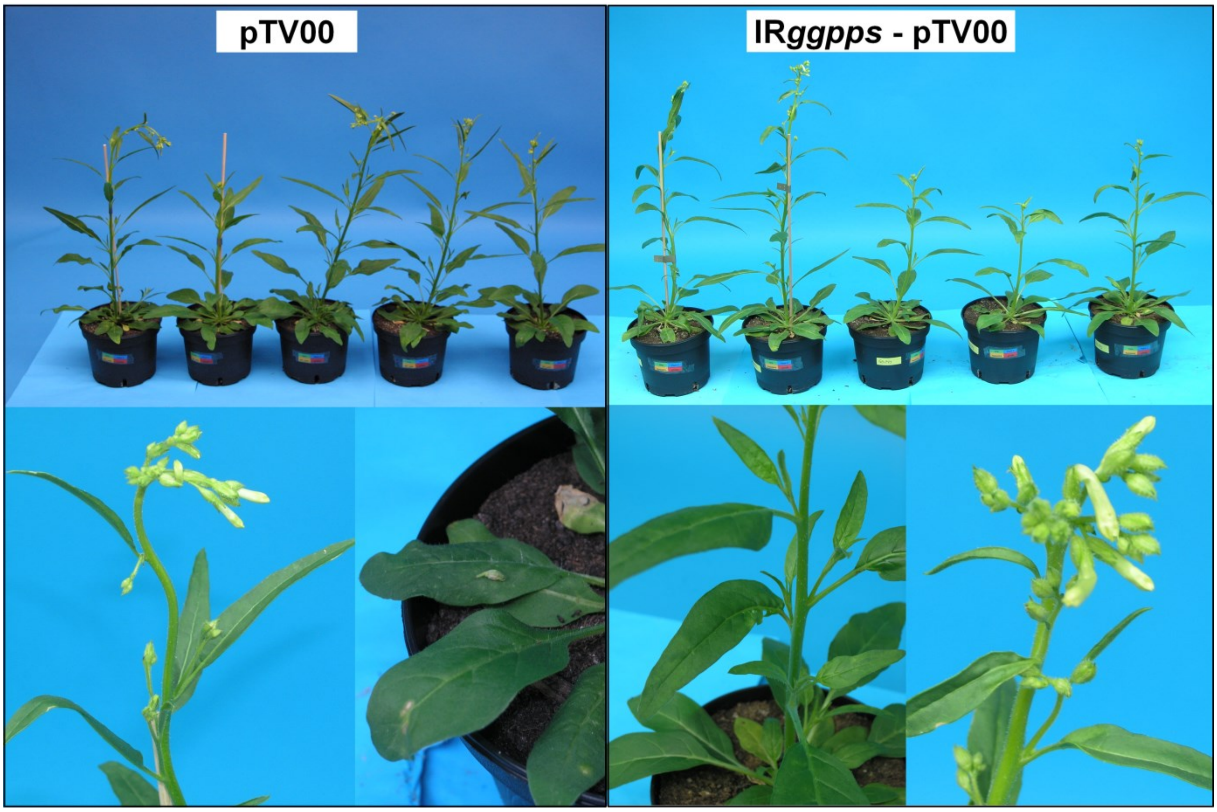

Supplemental Figure 25: Characterization of the morphological phenotypes of WT and IR*ggpps* plants transiently transformed with pTV00. (Supports Figure 7)

Shown are 42-day-old *N. attenuata* plants impaired in *GGPPS* expression and WT. Both are transiently transformed with the empty vector control and display typical morphological alterations like curly leaves due to the VIGS procedure.

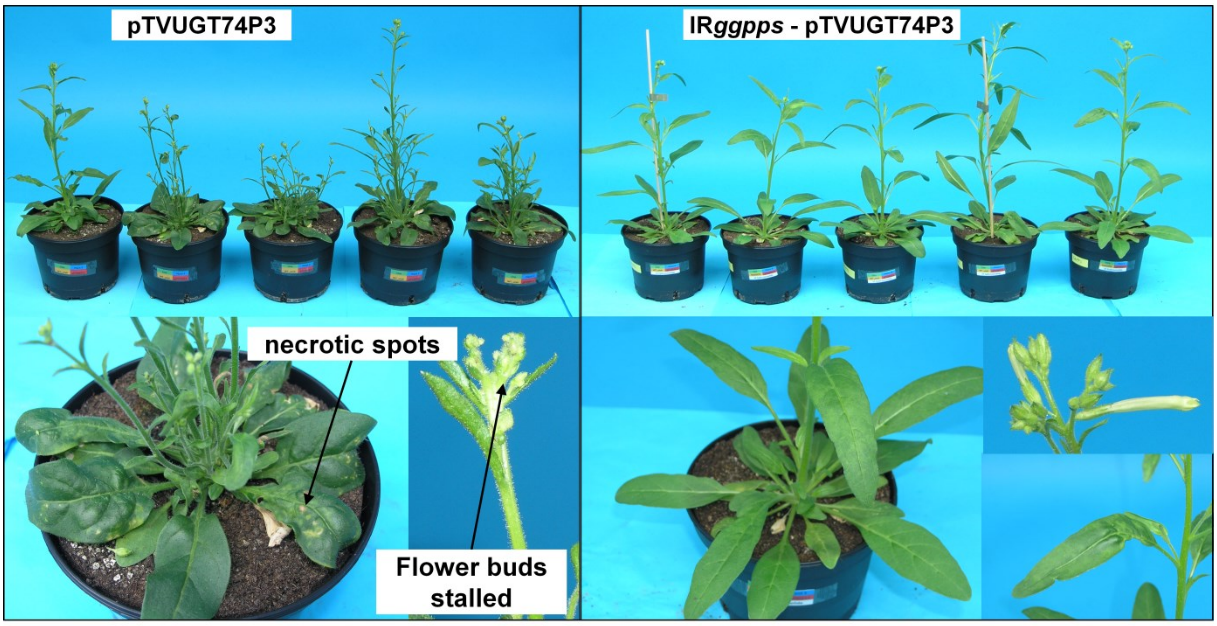

Supplemental Figure 26: Characterization of the morphological phenotypes of WT and IR*ggpps* plants transiently transformed with pTVUGT74P3. (Supports Figure 7)

Shown are 42-day-old *N. attenuata* plants impaired in *GGPPS* expression and WT. Both are transiently-transformed with the pTVUGT74P3 vector harboring a glucosyltransferase responsible for the synthesis of HGL-DTGs. WT VIGS plants display severe morphological alterations ranging from necrotic spots, lower stem height, enhanced branching and stalled or aborted flower buds. IR*ggpps* plants transiently transformed with pTVUGT74P3 only show slightly curly leaves.

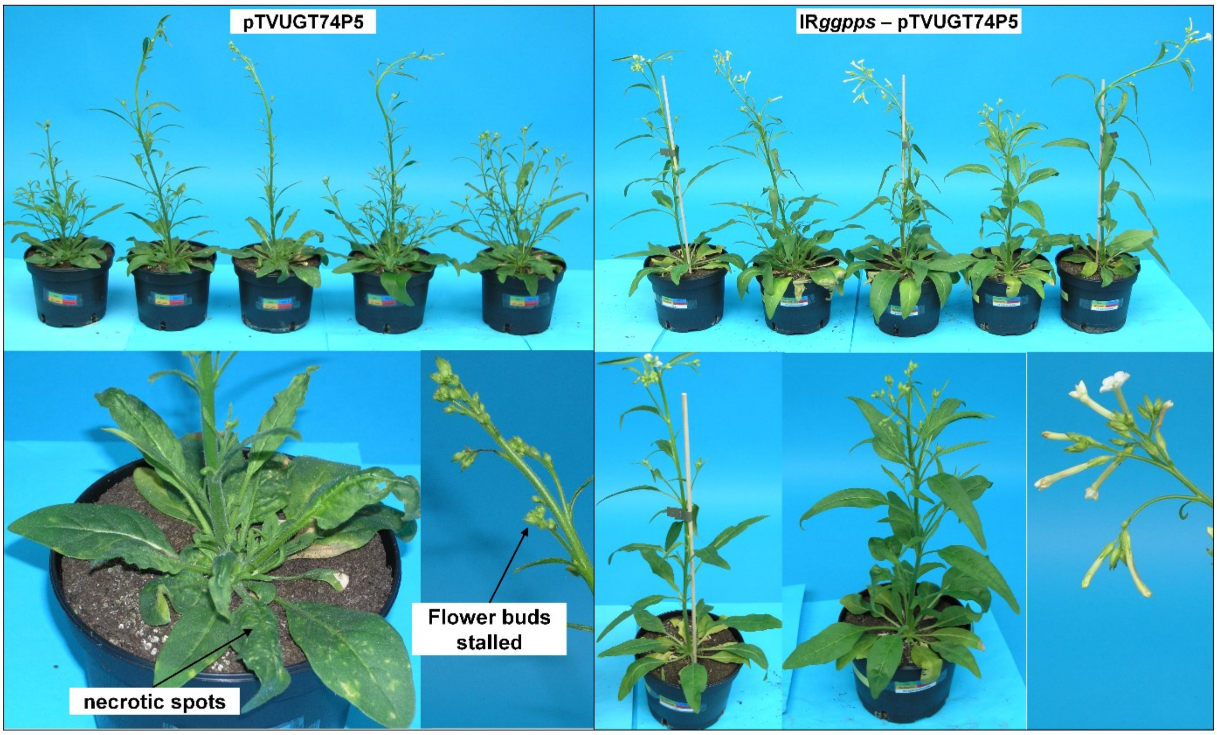

Supplemental Figure 27: Characterization of the morphological phenotypes of WT and IR*ggpps* plants transiently transformed with pTVUGT74P5. (Supports Figure 7)

Shown are 42-day-old *N. attenuata* plants impaired in *GGPPS* expression and WT. Both are transiently-transformed with the pTVUGT74P5 vector harboring a glucosyltransferase responsible for the synthesis of HGL-DTGs. WT VIGS plants display severe morphological alterations ranging from necrotic spots, enhanced branching and stalled or aborted flower buds. IR*ggpps* VIGS plants using pTVUGT74P5 only show slightly curly leaves.

Supplemental Table 1: Phylogenetic grouping of 107 UGTs in *N. attenuata*

Gr. No.	Group	Included families	NO. of sequences in group	% Similarity of full length protein sequences		
				MIN	MAX	AVERAGE
1	A	79,91,94	16+1	22.85	100.00	36.66
2	B	89	-	-	-	-
3	C	90	1	-	-	-
4	D	73	12	35.25	92.40	45.96
5	E	71,72,88,96,99	19	24.25	100.00	37.98
6	F	78	2	49.78	49.78	49.78
7	G	85	6	56.08	95.93	73.24
8	H	76	1	-	-	-
9	I	83,712	3	38.67	51.87	44.19
10	J	87	1	-	-	-
11	K	86	3	54.70	63.05	59.13
12	L	74,75,84	14+2	21.93	100.00	40.85
13	M	92	2	59.99	59.99	59.99
14	N	82	1	-	-	-
15	O	98, 93	17	35.04	82.58	47.59
16	P	709	5	47.42	55.23	51.70
17	Q	705	-	-	-	-
18	R	95	2	57.99	57.99	57.99

Supplemental Table 2: Molecular weight of UGTs

Protein Molecular Weight	Amino Acids	Sequence	starting
48.81 kDa	444	"UGT_g28668_CDS"	"MGTSQLHIAL"
49.36 kDa	437	"UGT_g38855_CDS"	"MASSLQNAAQ"
49.63 kDa	453	"UGT_g35881_CDS"	"MSNYHIAVLA"
49.95 kDa	444	"UGT_g24697_CDS"	"MEAKKNTISI"
49.95 kDa	444	"UGT_g41996_CDS"	"MEAKKNTISI"
49.96 kDa	455	"UGT_g23995_CDS"	"MDTDGRKVFG"
50.11 kDa	446	"UGT74P5_CDS"	"MEEITTKSHV"
50.45 kDa	445	"UGT_g22203_CDS"	"MVAKKNTISI"
50.53 kDa	450	"UGT91T1_CDS"	"MEEITSQKTH"
50.58 kDa	450	"UGT_g30507_CDS"	"MKCRKKTKVL"
50.66 kDa	449	"UGT_g40889_CDS"	"MVPFPAQGHL"
50.88 kDa	456	"UGT_g01854_CDS"	"MGHLIPLVEF"
51.05 kDa	456	"UGT_g04160_CDS"	"MKKHHFLIIS"
51.06 kDa	454	"UGT_g30575_CDS"	"MGSCENLPNH"
51.13 kDa	460	"UGT_g10741_CDS"	"MVPIPHIVAI"
51.34 kDa	469	"UGT_g16298_CDS"	"MSNSKNGVHV"
51.40 kDa	458	"UGT_g18508_CDS"	"MELPSQIKCH"
51.57 kDa	458	"UGT_g11522_CDS"	"MSHKAHCLIL"
51.57 kDa	458	"UGT_g43590_CDS"	"MSHKAHCLIL"
51.65 kDa	459	"UGT_g11521_CDS"	"MTTHKAHCLI"
51.71 kDa	458	"UGT_g22205_CDS"	"MDTQVTECGN"
51.77 kDa	466	"UGT_g11850_CDS"	"MRKNDVHVVM"
52.03 kDa	457	"UGT_g24585_CDS"	"MDTQHSSLRV"
52.06 kDa	459	"UGT_g41120_CDS"	"MAKTSNLENH"
52.07 kDa	465	"UGT_g03748_CDS"	"MASPPCIHVV"
52.09 kDa	461	"UGT_g35131_CDS"	"MSTTHKAHCL"
52.17 kDa	464	"UGT_g21652_CDS"	"MEILKDDCHV"
52.21 kDa	464	"UGT_g13538_CDS"	"MIGKRNEPHI"
52.23 kDa	465	"UGT_g21654_CDS"	"MKKHHYLVIS"
52.37 kDa	465	"UGT_g02821_CDS"	"MANKYEVVVV"
52.52 kDa	479	"UGT_g23176_CDS"	"MAETAIVPKS"
52.53 kDa	472	"UGT_g06355_CDS"	"MENEKSNDVL"
52.53 kDa	463	"UGT_g25918_CDS"	"MDLQGITSLR"
52.54 kDa	466	"UGT_g30577_CDS"	"MADKCTSNVA"
52.54 kDa	467	"UGT_g33134_CDS"	"MDIKSENLAN"
52.56 kDa	477	"UGT74P3_CDS"	"MKKDSIHVVM"
52.56 kDa	470	"UGT_g28046_CDS"	"MTRSELVFVP"
52.57 kDa	468	"UGT_g02942_CDS"	"MATINHATQH"
52.67 kDa	470	"UGT_g28047_CDS"	"MKKAELVFVP"
52.69 kDa	467	"UGT_g20123_CDS"	"MAANSNNVAV"
52.91 kDa	476	"UGT_g31158_CDS"	"MAETPRATQS"
52.91 kDa	476	"UGT_g35911_CDS"	"MAETPRATQS"
52.94 kDa	474	"UGT_g11851_CDS"	"MSKSDVHVVM"
52.99 kDa	468	"UGT_g39264_CDS"	"MENLKNECHV"
53.00 kDa	473	"UGT_g19344_CDS"	"MVQPHVLLVT"
53.03 kDa	476	"UGT_g16426_CDS"	"MDSSQLHVAI"
53.10 kDa	481	"UGT_g37675_CDS"	"MAETQSLTKP"
53.11 kDa	474	"UGT_g24514_CDS"	"MLSMDPSAFV"
53.22 kDa	473	"UGT_g05573_CDS"	"MKNGKKPLHV"
53.28 kDa	472	"UGT_g22983_CDS"	"MANPLDLKKH"
53.31 kDa	467	"UGT_g29262_CDS"	"MADTKKLHVV"
53.38 kDa	472	"UGT_g05060_CDS"	"MASNFHFHNN"
53.38 kDa	474	"UGT_g40324_CDS"	"MSKLELVFVP"
53.41 kDa	475	"UGT_g38610_CDS"	"MKVEKRQSVV"
53.42 kDa	474	"UGT_g36713_CDS"	"MEEIMGEILV"
53.47 kDa	479	"UGT_g32894_CDS"	"MKTAELVFIP"

53.55 kDa	468	"UGT_g22204_CDS"	"MNIFFRKKKV"
53.61 kDa	473	"UGT_g3727_CDS"	"MASNLHFQNH"
53.62 kDa	478	"UGT_g27477_CDS"	"MGVEEHPKLH"
53.62 kDa	480	"UGT_g31196_CDS"	"MDRSQLHIAI"
53.63 kDa	477	"UGT_g23136_CDS"	"MGQLHFFFFP"
53.64 kDa	483	"UGT_g16288_CDS"	"MKKAKVVFIS"
53.67 kDa	469	"UGT_g00527_CDS"	"MAENSKKLHI"
53.67 kDa	481	"UGT_g19018_CDS"	"MSKHHILMIP"
53.77 kDa	483	"UGT_g13005_CDS"	"MATAEQKEKP"
53.77 kDa	476	"UGT_g9179_CDS"	"MAPSCNHNQN"
53.80 kDa	478	"UGT_g24515_CDS"	"MALPHVLIFP"
53.81 kDa	474	"UGT_g00526_CDS"	"MLPWLAFGHI"
53.99 kDa	483	"UGT_g32892_CDS"	"MDTKKPELVF"
54.00 kDa	485	"UGT_g19190_CDS"	"MGSIGAELTK"
54.04 kDa	483	"UGT_g02083_CDS"	"MNQMPNNTLI"
54.05 kDa	483	"UGT_g04820_CDS"	"MKDTKKIELV"
54.05 kDa	483	"UGT_g34878_CDS"	"MDRKTDQLHI"
54.11 kDa	484	"UGT_g22481_CDS"	"MSSLRGKKAQ"
54.19 kDa	481	"UGT_g38353_CDS"	"MSDQLHFFFF"
54.20 kDa	483	"UGT_g40325_CDS"	"MESRELVFIP"
54.21 kDa	479	"UGT_g27096_CDS"	"MNELIFIPLA"
54.24 kDa	482	"UGT_g32016_CDS"	"MGSIRQDKPH"
54.42 kDa	486	"UGT_g34877_CDS"	"MDKRTDQLHI"
54.48 kDa	484	"UGT_g34091_CDS"	"MASKYAKLDM"
54.55 kDa	497	"UGT_g02093_CDS"	"MAAQKSQVHF"
54.87 kDa	484	"UGT_g21846_CDS"	"MDEPASCLSH"
54.88 kDa	492	"UGT_g09327_CDS"	"MGSIIADLEK"
54.93 kDa	495	"UGT_g20981_CDS"	"MFENRPHALI"
54.98 kDa	492	"UGT_g09326_CDS"	"MGSIIAELKK"
54.99 kDa	498	"UGT_g38211_CDS"	"MTVSSTSQQL"
55.04 kDa	493	"UGT_g36017_CDS"	"MASLVIPSSG"
55.18 kDa	488	"UGT_g29220_CDS"	"MEPEENIKPH"
55.21 kDa	490	"UGT_g05219_CDS"	"MDSQLLIKSM"
55.26 kDa	490	"UGT_g05426_CDS"	"MAVLTNEQPH"
55.33 kDa	486	"UGT_g26396_CDS"	"MNQESLPPHV"
55.39 kDa	491	"UGT_g28311_CDS"	"MSSTNSQKLH"
55.46 kDa	494	"UGT_g12088_CDS"	"MGSTVGEGAL"
55.47 kDa	488	"UGT_g28309_CDS"	"MAVLTIQPHF"
55.51 kDa	488	"UGT_g11159_CDS"	"MGILTVQPHF"
55.63 kDa	501	"UGT_g15196_CDS"	"MGSSEKLHVG"
55.67 kDa	495	"UGT_g03342_CDS"	"MDHPSPHVLL"
55.73 kDa	493	"UGT_g18870_CDS"	"MTQENGKLHV"
55.78 kDa	499	"UGT_g30898_CDS"	"MDNGNSNGNG"
55.91 kDa	493	"UGT_g19346_CDS"	"MASLSKQLHF"
56.11 kDa	513	"UGT_g24726_CDS"	"MEAIGDILVL"
56.16 kDa	496	"UGT_g34991_CDS"	"MEGKKGNIIL"
56.23 kDa	508	"UGT_g28331_CDS"	"MGSQGTIPPP"
56.25 kDa	497	"UGT_g30863_CDS"	"MASHYQKTHA"
57.82 kDa	511	"UGT_g08104_CDS"	"MEKNKGTILF"
58.46 kDa	514	"UGT_g29493_CDS"	"MGGCASLDQS"

Supplemental Table 3: SignalIP4.1 – signal peptide cleavage sites

##sequence-name	source	feature	start	end	score	N/A?
NIATv7_g28668_CDS	SignalP-4.1	SIGNAL	1	19	0.569	YES
NIATv7_g29262_CDS	SignalP-4.1	SIGNAL	1	18	0.512	YES

Supplemental Table 5a: Pearson Correlation of all UGTs to NaGLS				
	**Pearson correlation to NaGLS (p-Value <0.05)**			
**Gene ID**	**Total treated leaves**	**Total systemic leaves**	**Total roots**	**Total all tissues**
NaGLS	1	1	1	1
NaUGT91T1	0.867	0.851	0.437	0.899
NaUGT74P3	0.765	0.799	0.303	0.872
NaUGT74P5	0.795	0.788	0.293	0.868
NaGGPPS	0.576	0.720	#	0.799
NaUGT_g35131	0.579	0.663	#	0.747
NaUGT_g06355	0.716	0.731	#	0.710
NaUGT_g24514	0.253	0.278	#	0.551
NaUGT_g16426	0.459	0.373	#	0.527
NaUGT_g11159	0.401	0.641	#	0.501
NaUGT_g28668	0.215	0.229	0.243	0.504
NaUGT_g15196	0.198	0.259	#	0.420
NaUGT_g18508	0.194	0.287	-0.234	0.434
NaUGT_g04160	0.337	0.284	#	0.397
NaUGT_g32892	-0.254	-0.377	0.331	0.391
NaUGT_g02942	0.212	#	#	0.379
NaUGT_g40325	0.476	0.215	#	0.380
NaUGT_g13005	-0.173	#	#	0.352
NaUGT_g40324	0.450	0.526	0.205	0.350
NaUGT_g22983	#	-0.198	#	0.326
NaUGT_g29220	0.162	#	0.434	0.330
NaUGT_g03748	0.220	0.173	#	0.330
NaUGT_g21652	-0.329	-0.513	#	0.325
NaUGT_g30863	0.333	0.691	#	0.292
NaUGT_g13538	#	0.521	#	0.284
NaUGT_g20123	#	0.163	#	0.259
NaUGT_g24515	0.410	0.287	#	0.262
NaUGT_g33134	#	0.186	#	0.250
NaUGT_g05426	#	#	#	0.229
NaUGT_g36017	0.225	0.421	#	0.191
NaUGT_g16298	0.393	0.475	#	#
NaUGT_g35881	0.199	#	#	#
NaUGT_g30507	#	0.384	#	#
NaUGT_g24726	#	#	#	#
NaUGT_g34877	#	0.322	0.290	#
NaUGT_g32894	0.244	#	#	#
NaUGT_g25918	#	-0.184	#	#
NaUGT_g19344	#	#	0.199	#
NaUGT_g28331	-0.250	-0.562	#	#
NaUGT_g23136	#	#	#	#

NaUGT_g02083	0.158	0.337	#	#
NaUGT_g22481	#	-0.233	-0.190	#
NaUGT_g27477	#	#	#	#
NaUGT_g11521	#	0.267	#	#
NaUGT_g9179	#	#	#	#
NaUGT_g41120	#	#	0.166	#
NaUGT_g28311	#	#	#	#
NaUGT_g04820	#	0.348	0.373	#
NaUGT_g41996	#	#	#	#
NaUGT_g22205	#	0.172	0.165	#
NaUGT_g29493	0.239	#	#	#
NaUGT_g23176	#	#	#	#
NaUGT_g08104	#	#	0.173	#
NaUGT_g43590	#	0.320	#	-0.203
NaUGT_g20981	#	#	#	-0.360
NaUGT_g05060	0.161	0.175	0.189	-0.360
NaUGT_g26396	0.479	0.359	#	-0.368
NaUGT_g38211	0.227	0.694	#	-0.378
NaUGT_g34091	-0.223	-0.176	#	-0.402
NaUGT_g03342	-0.326	-0.437	#	-0.442
NaUGT_g16288	-0.199	-0.213	#	-0.476
NaUGT_g31196	#	0.166	#	-0.497
NaUGT_g01854	#	0.340	#	-0.498
NaUGT_g05573	0.161	0.288	0.164	-0.506
NaUGT_g40889	#	#	0.330	-0.512
NaUGT_g00526	0.183	#	#	-0.536
NaUGT_g05219	#	-0.245	#	-0.546
NaUGT_g22203	0.222	0.347	0.290	-0.545
NaUGT_g29262	0.220	0.309	#	-0.551
NaUGT_g39264	-0.243	-0.175	#	-0.558
NaUGT_g32016	-0.195	#	#	-0.562
NaUGT_g24585	-0.302	#	0.197	-0.575
NaUGT_g09326	-0.164	0.473	#	-0.576
NaUGT_g19190	-0.193	0.437	#	-0.589
NaUGT_g09327	-0.212	0.286	#	-0.597
NaUGT_g00527	-0.358	-0.307	0.183	-0.603
NaUGT_g02093	-0.298	-0.354	-0.210	-0.616
NaUGT_g34991	-0.449	-0.476	-0.225	-0.686

[#]missing values are not significant

Supplemental Table 4b: Pearson Correlation of all UGTs to NaGGPPS

	Pearson correlation to NaGGPPS (p-Value <0.05)			
**Gene ID**	**Total treated leaves**	**Total systemic leaves**	**Total roots**	**Total all tissues**
NaGGPPS	1.000	1.000	1.000	1.000
NaUGT91T1	0.544	0.782	0.301	0.823
NaGLS	0.576	0.720	#	0.799
NaUGT_g18508	0.711	0.742	0.499	0.722
NaUGT74P5	0.438	0.329	#	0.608
NaUGT74P3	0.310	0.307	#	0.566
NaUGT_g03748	0.574	0.516	0.274	0.526
NaUGT_g13538	0.245	0.859	#	0.505
NaUGT_g35131	0.246	0.221	-0.426	0.498
NaUGT_g30507	0.680	0.787	-0.204	0.457
NaUGT_g24514	#	#	0.465	0.432
NaUGT_g30863	0.593	0.802	0.276	0.420
NaUGT_g06355	0.170	0.311	0.181	0.408
NaUGT_g11159	0.485	#	#	0.365
NaUGT_g15196	#	#	-0.207	0.291
NaUGT_g21652	-0.445	-0.463	#	0.284
NaUGT_g32892	-0.571	-0.556	0.160	0.265
NaUGT_g11521	0.534	0.657	-0.435	0.258
NaUGT_g28668		-0.176	0.229	0.256
NaUGT_g05426	0.240	-0.198	#	0.251
NaUGT_g24726	0.503	-0.326	#	0.242
NaUGT_g22481	0.443	#	#	0.233
NaUGT_g22983	#	-0.502	0.255	0.218
NaUGT_g20123	#	#	#	0.199
NaUGT_g33134	-0.213	0.162	0.188	0.186
NaUGT_g9179	0.461	#	0.594	0.176
NaUGT_g19344	0.354	-0.294	-0.385	0.171
NaUGT_g32894	0.534	#	-0.372	0.164
NaUGT_g23136	0.449	-0.381	-0.465	#
NaUGT_g13005	-0.416	-0.523	0.273	#
NaUGT_g34877	#	0.159		#
NaUGT_g16298	0.557	0.267	-0.273	#
NaUGT_g41120	0.307	-0.393	-0.379	#
NaUGT_g28311	0.236	#	-0.164	#
NaUGT_g22205	0.360	0.179	-0.362	#
NaUGT_g41996	#	0.265	0.193	#
NaUGT_g16426	-0.218	-0.221	#	#
NaUGT_g04820	0.199	0.177	-0.242	#
NaUGT_g35881	#	#	#	#

NaUGT_g02942	-0.306	-0.352	0.183	#
NaUGT_g28331	-0.350	-0.515	#	#
NaUGT_g36017	#	#	#	#
NaUGT_g04160	-0.242	-0.216	0.387	#
NaUGT_g24515	0.214	-0.218	-0.455	#
NaUGT_g02083	#	0.168	-0.242	#
NaUGT_g43590	0.493	0.695	-0.508	#
NaUGT_g29220	-0.431	-0.407		#
NaUGT_g40325	#	-0.292	0.438	#
NaUGT_g29493	#	0.202	#	#
NaUGT_g25918	-0.353	-0.315	#	#
NaUGT_g40324	-0.197	#	0.232	#
NaUGT_g23176	0.222	-0.394	0.525	#
NaUGT_g20981	0.342	0.389	-0.268	-0.164
NaUGT_g27477	-0.445	-0.245	0.606	-0.176
NaUGT_g38211	0.651	0.915	0.267	-0.257
NaUGT_g34091	-0.212	#	-0.238	-0.329
NaUGT_g26396	0.646	0.158	#	-0.345
NaUGT_g05060	-0.159	#	-0.430	-0.365
NaUGT_g08104	-0.308	-0.425	-0.424	-0.384
NaUGT_g39264	#	#	0.541	-0.444
NaUGT_g40889	0.397	#	-0.260	-0.448
NaUGT_g31196	#	#	0.425	-0.465
NaUGT_g01854	#	0.175	-0.239	-0.469
NaUGT_g05573	-0.183	#	-0.393	-0.493
NaUGT_g00526	#	#		-0.505
NaUGT_g03342	-0.570	-0.527	-0.175	-0.511
NaUGT_g34991	#	#	#	-0.514
NaUGT_g22203	#	0.179	-0.166	-0.514
NaUGT_g24585	-0.308	0.314	-0.504	-0.515
NaUGT_g29262	#	0.158	0.395	-0.517
NaUGT_g32016	-0.249	#	#	-0.526
NaUGT_g16288	-0.537	-0.371	-0.247	-0.540
NaUGT_g09326	-0.188	#	#	-0.544
NaUGT_g19190	-0.198	#	#	-0.551
NaUGT_g09327	-0.208	#	#	-0.557
NaUGT_g02093	-0.401	-0.383	#	-0.599
NaUGT_g00527	-0.462	-0.469	#	-0.600
NaUGT_g05219	-0.494	-0.573	0.308	-0.609

[#]missing values are not significant

Supplemental Methods 1

GTs are highly divergent, polyphyletic, and represent one of the largest multigene super-families in plant genomes (Mackenzie et al., 1997; Ross et al., 2001). GTs catalyze the transfer of activated sugars as donor molecules to specific acceptors, including sugars, lipids, proteins, nucleic acids as well as small hydrophilic molecules (Weadge, 2000; Lairson et al., 2008). GTs have been classified into 103 families according to their sequence similarity to other carbohydrate-active enzymes, their catalytic mechanisms (inverting and retaining), 3D-structures (Coutinho et al., 2003), sugar donors, transferred sugars, acceptors and the combination of modules which can be catalytic or not (Lombard et al., 2014) as well as the presence of conserved sequence motifs (Campbell et al., 1997; 1998, CAZY - http://www.cazy.org). Family 1 corresponds to the uridine-5'-phosphate (UDP)-glycosyltransferases (UGTs) which are characterized by the utilization of UDP-activated sugars, like UDP-glucose, UDP-rhamnose, UDP-galactose and UDP-xylose as donor molecules (Merken and Beecher, 2000) for the glycosylation of complex structurally variable substrates like flavonoids, terpenes, and even phytohormones. While most UGTs perform O-glycosylations, for some xenobiotics however, UDP-glucose is used to glycosylate N-, S- or even C-linkages (Brazier-Hicks et al., 2009). Plant UGTs are promiscuous and are known to have broad substrate specificity, which is generally limited by regiospecificity (Lim et al., 2003). However, in some cases UGTs have also been shown to be highly specific. The diversity of used substrates is thought to result from the high variability in the N-terminal region of UGTs (Bowles et al., 2005; Bowles et al., 2006). The C-terminal region contains a conserved 44 amino acid sequence motif known as the plant secondary product glycosyltransferase (PSPG)-box, which is thought to be the UDP-sugar binding site (Vogt and Jones, 2000) and is often the only region of significant similarity in sequence alignments. Plant UGTs of the family 1 have been thoroughly studied across the plant kingdom, including *Arabidopsis thaliana* (Li et al., 2001; Ross et al., 2001; Paquette et al., 2003), *Glycine max* (Rehman et al., 2016), *Gossypium hirsutum* (Huang et al., 2015), *Linum usitatissimum* (Barvkar et al., 2012), *Medicago truncatula* (Achnine et al., 2005) and *Zea mays* (Li et al., 2014). These genome-wide

investigations provide systematic and global insights into the relationship between the structure and function of this multigene family.

Genome-wide inference and phylogenetic analysis of *N. attenuata* UGTs

A genome-wide survey of *N. attenuata* identified a total of 107 putative UGT sequences containing the PSPG motif at the C-terminus (Supplemental Figure 1). The length of the deduced proteins varied from 437 – 514 aa, averaging 474 aa with a predicted molecular weight ranging from 49 kDa to 58 kDa (Supplemental Table 2). All UGT sequences started with a methionine, none of these sequences contained premature stop codons. The constructed phylogenetic tree resulted in the classification of UGT protein sequences into 18 major groups (A-R) (Supplemental Figure 2). These groups are consistent with the previously described classification of UGTs in Arabidopsis (Li et al., 2001; Ross et al., 2001), maize (Li et al., 2014), flax (Barvkar et al., 2012) or cotton (Huang et al., 2015; Rehman et al., 2016) with the exception of the subfamily UGT95 which did not cluster with the UGT92 subfamily as part of group M (Huang et al., 2015). We therefore created a new group R, specific to *N. attenuata* that contains the UGT95 proteins. Group Q was absent in the classification of *N. attenuata* UGTs. Proteins classified as part of the same phylogenetic group exhibit percentages of aa similarity ranging from 22 % to 100 % (Supplemental Table 1). Highest average similarities were detected in group G with 73% and groups K and M with 59%, while the lowest were detected in groups A (23%), E (24%) and L (22%). Altogether, the 105 UGTs of *N. attenuata* clustered into 26 subgroups (sequence similarity of ~40% according to UGT nomenclature) and only two UGT sequences (NaUGTg20981; NaUGTg16298) did not cluster in any subgroup and remained unclassified.

To characterize the identified UGTs, we analyzed the amino acid composition and compared it to its soybean relatives. Leucine was the most abundant amino acid across all UGTs from *N. attenuata* (10%). Cysteine, histidine, methionine, tryptophan and tyrosine were the least common amino acids (1.6 – 2.7%) (Supplemental Figure 3, Supplemental Data Set 1). These results are consistent with those observed in soybean (Rehman et al., 2016).

We used SignalP4.1 to identify whether UGT proteins contain signal peptides for subcellular localization and detected that only two sequences (NaUGTg28668, NaUGTg29262) contained such motifs (Supplemental Table 3).

Using a full-transcriptome microarray dataset obtained from leaf and root tissues collected at several time-points following simulated leaf herbivory by application of *Manduca sexta* oral secretions (OS), we detected that, of the 107 UGTs identified in *N. attenuata*, 76 were expressed in either leaves or roots, while 31 were not expressed in these two tissue types (Supplemental Figure 4, Supplemental Data Set 2). The most pronounced changes in UGT transcript abundance appeared after 1h in locally OS-treated leaves while in systemic leaves and roots, induced levels of UGT expression peaked after 5 h. Consistent with the observation that UGTs often play pivotal metabolic functions in response to insect herbivory, only 12 UGTs were not affected by the OS treatment. To explore the tissue-level expression of the identified UGTs, we mined a previously published RNA-seq atlas established for 21 different tissue types from *N. attenuata* (Brockmoller et al., 2017). In addition to the previously mentioned 76 UGTs detected in leaves and roots after simulated herbivory, we found 17 UGTs expressed in different tissues other than leaves and roots. For the remaining 14 UGTs we did not detect any significant transcript abundance levels (Supplemental Figure 4). 45 UGTs were differentially expressed (2-fold change relative to controls) 1h after the simulated herbivory treatment, 41 UGTs after 5 h and 27 UGTs after 17 h (Supplemental Figure 5).

Phylogenetic characterization of the UGT family 1 in *N. attenuata*

UGTs represent 0.29% (137/47912), of all genes in flax (Barvkar et al., 2012), 0.33 – 0.38% (142/37505, *G. raimondii*; 146/40134 *G. arboreum*; 196/59089, *G. hirsutum)*; in different cotton species (Huang et al., 2015) and up to 0.44% of all genes in *Arabidopsis (120/27416*, (Paquette et al., 2003)). In wild and cultivated tobacco species, only a handful of family 1 UGTs have been functionally characterized. This includes a salicylic acid glucosyltransferase (SAGT – (Lee and Raskin, 1999)), several UGTs responsible for the glucosylation of phenolics, especially naphthols (*Nt*GT1a, *Nt*GT1b and *Nt*GT3 – (Taguchi et al., 2001; Taguchi et al., 2003)) and two putative flavonoid UDP-glycosyltransferases (UGT-A, UGT-B – Li et al., 2016). To enrich our understanding of this family in *N. attenuata*, we performed

a genome-wide analysis for the identification of UGTs and analyzed their phylogenetic relationships. We identified 107 putative UGTs and showed that the family-1 UGTs represent 0.32% (107/33449 – Xu et al., 2017) of the expressed genes in the genome, which is less than in *Arabidopsis* but about the same as in flax or cotton.

Additionally, we found that five phylogenetic groups, namely A, D, E, L and O, seem to have expanded more than others (Supplemental Table 1) in *N. attenuata*. This observation has been made in earlier studies in other higher plants (Caputi et al., 2012; Rehman et al., 2016). We foresee that this phylogenetic analysis in combination with expression studies provides an instrumental data platform for further characterization of UGT functions in the genus *Nicotiana*.

Material and Methods

Identification of UDP-glycosyltransferase sequences in *N. attenuata*

Sequences of the *N. attenuata* genome (MJEQ00000000) and a 454 *N. attenuata* shotgun assembly (GBGF00000000) were used. To identify members of the UGT family, we used the 44-amino acid conserved sequence of the plant secondary product glycosyltransferase (PSPG)-box motif as a query to perform a local BLASTP search. The e-Value threshold was set to 1e-10. All UGT candidates were verified using HMMER (http://www.ebi.ac.uk/Tools/hmmer) to confirm the presence of the UDP-glycosyltransferase domain (pfam00201). The identified UGTs were named based on the *N. attenuata* gene identifiers. Verified UGTs with a proven function were classified and named based on the HUGO Gene Nomenclature Committee (Mackenzie et al., 1997).

Phylogenetic analysis of *N. attenuata* UGTs

Identified UGT sequences were aligned using Clustal W with default gap penalties, the phylogenetic tree was constructed using maximum-likelihood (JTT matrix-based model, bootstrap value: 1000 replicates) and neighbor joining (bootstrap value: 1000, p-distance and pairwise deletion) in MEGA 5.0 (http://www.megasoftware.net). Twenty-eight reference UGT peptide sequences

belonging to various phylogenetic groups (A-O) were used for the phylogenetic comparison (Supplemental Figure 2). Subgroups were classified based on sequence similarity to the twenty-eight UGTs. Sequence similarity of the subgroup UGT93 was calculated based on the alignment to ZOG1 and ZOX1 from *Phaseolus vulgaris* (ZOX1 AAD51778; ZOG1 AAD04166) via Geneous (http://www.geneous.com). The total amino acid composition was calculated using the MEGA sequence data explorer tool. The molecular mass was calculated using Protein Molecular Weight (http://www.bioinformatics.org/sms2/protein_mw.html).

Microarray data sets

To explore the temporal expression dynamics of *N. attenuata* UGTs, we mined a dataset produced by our laboratory that is publicly available at the Gene Expression Omnibus database (accession number GSE30287), and consists of 150 published microarray expression profiles. This microarray data set was originally published in (Kim et al., 2011) and its experimental design was as follows. To simulate *Manduca sexta* feeding, the laminas of three leaves per plant (two source leaves at nodes +2 and +1 and one source-sink transition leaf at node 0) were mechanically wounded with a fabric pattern wheel on both sides of the midrib, and immediately, 20 µL of *M. sexta* OS (diluted 1:10 in water) was applied to the fresh puncture wounds (W+OS). For each time point (1, 5, 9, 13, 17, and 21 h after treatment), treated leaves or control leaves at the same nodal positions, systemic leaves (two sink leaves at nodes −1 and −2), and the complete root system were collected from six plants and immediately flash frozen in liquid nitrogen. As described by Gulati et al. (Gulati et al., 2013; Gulati et al., 2014), raw intensities of the microarray data-set were normalized using the 75th percentile value procedure and $\log_2$ and baseline transformed prior to statistical analysis. We compared the expression of all identified UGTs at 1h, 5h and 17h in local, systemic and root tissue using Tukeys post hoc test (p-Value < 0.05). The correlation to *NaGGPPS* and *NaGLS* was performed using Pearson correlation calculations across all 134 microarrays. Significance levels for correlation values (*r*) were determined following the number of transcript pairs (*n*) using the equation $t = r \times (n-2)^{0.5} / (1-r)^{0.5}$.

For the expression of the UGTs in other tissues we analyzed an RNAseq dataset (PRJNA317743) of 21 different tissues in *N. attenuata*.

For the construction of the UDP-glycosyltransferase tree in *N. attenuata*, we used the following sequence data which can be found in the GenBank database under the following accessions: NaUGT g00526 - KX752100, NaUGT g00527 - KX752101, NaUGT g01854 - KX752102, NaUGT g02083 - KX752103, NaUGT g02093 - KX752104, NaUGT g02821 - KX752105, NaUGT g02942 - KX752106, NaUGT g03342 - KX752107, NaUGT g3727 - KX752108, NaUGT g03748 - KX752109, NaUGT g04160 - KX752110, NaUGT g04820 - KX752111, NaUGT g05060 - KX752112, NaUGT g05219 - KX752113, NaUGT g05426 - KX752114, NaUGT g05573 - KX752115, NaUGT g06355 - KX752116, NaUGT g08104 - KX752117, NaUGT g9179 - KX752118, NaUGT g09326 - KX752119, NaUGT g09327 - KX752120, NaUGT g10741 - KX752121, NaUGT g11159 - KX752122, NaUGT g11521 - KX752123, NaUGT g11522 - KX752124, NaUGT g11850 - KX752125, NaUGT g11851 - KX752126, NaUGT g12088 - KX752127, NaUGT g13005 - KX752128, NaUGT g13538 - KX752129, NaUGT g13945 - KX752130, NaUGT g15196 - KX752131, NaUGT g16288 - KX752132, NaUGT g16298 - KX752133, NaUGT g16426 - KX752134, NaUGT g18508 - KX752135, NaUGT g18870 - KX752136, NaUGT g19018 - KX752137, NaUGT g19190 - KX752138, NaUGT g19344 - KX752139, NaUGT g19346 - KX752140, NaUGT g20123 - KX752141, NaUGT74P5 - KX752142, NaUGT g20981 - KX752143, NaUGT g21652 - KX752144, NaUGT g21654 - KX752145, NaUGT g21846 - KX752146, NaUGT g22203 - KX752147, NaUGT g22204 - KX752148, NaUGT g22205 - KX752149, NaUGT g22481 - KX752150, NaUGT g22983 - KX752151, NaUGT g23136 - KX752152, NaUGT g23176 - KX752153, NaUGT g23995 - KX752154, NaUGT g24514 - KX752155, NaUGT g24515 - KX752156, NaUGT g24585 - KX752157, NaUGT g24697 - KX752158, NaUGT g24726 - KX752159, NaUGT g25918 - KX752160, NaUGT g26396 - KX752161, NaUGT91T1 - KX752162, NaUGT g27096 - KX752163, NaUGT g27477 - KX752164, NaUGT g28046 - KX752165, NaUGT g28047 - KX752166, NaUGT g28309 - KX752167, NaUGT g28311 - KX752168, NaUGT g28331 - KX752169, NaUGT g28668 - KX752170, NaUGT g29220 - KX752171, NaUGT g29262 - KX752172, NaUGT g29493 - KX752173, NaUGT g30507 - KX752174, NaUGT g30575 - KX752175, NaUGT g30577 - KX752176, NaUGT g30863 - KX752177, NaUGT g30898 - KX752178, NaUGT g31158 - KX752179, NaUGT g31196 - KX752180, NaUGT g32016 - KX752181, NaUGT

g32892 - KX752182, NaUGT g32894 - KX752183, NaUGT g33134 - KX752184, NaUGT g34091 - KX752185, NaUGT g34877 - KX752186, NaUGT g34878 - KX752187, NaUGT g34991 - KX752188, NaUGT g35131 - KX752189, NaUGT g35881 - KX752190, NaUGT g35911 - KX752191, NaUGT g36017 - KX752192, NaUGT g36713 - KX752193, NaUGT g37675 - KX752194, NaUGT g38211 - KX752195, NaUGT g38353 - KX752196, NaUGT g38610 - KX752197, NaUGT g38855 - KX752198, NaUGT g39264 - KX752199, NaUGT g40324 - KX752200, NaUGT g40325 - KX752201, NaUGT g40889 - KX752202, NaUGT g41120 - KX752203, NaUGT74P3 - KX752204, NaUGT g41996 - KX752205, NaUGT g43590 - KX752206.

References

Achnine, L., Huhman, D.V., Farag, M.A., Sumner, L.W., Blount, J.W., and Dixon, R.A. (2005). Genomics-based selection and functional characterization of triterpene glycosyltransferases from the model legume *Medicago truncatula*. Plant J **41,** 875-887.

Barvkar, V.T., Pardeshi, V.C., Kale, S.M., Kadoo, N.Y., and Gupta, V.S. (2012). Phylogenomic analysis of UDP glycosyltransferase 1 multigene family in *Linum usitatissimum* identified genes with varied expression patterns. Bmc Genomics **13**.

Bowles, D., Isayenkova, J., Lim, E.K., and Poppenberger, B. (2005). Glycosyltransferases: managers of small molecules. Curr Opin Plant Biol **8,** 254-263.

Bowles, D., Lim, E.K., Poppenberger, B., and Vaistij, F.E. (2006). Glycosyltransferases of lipophilic small molecules. Annu Rev Plant Biol **57,** 567-597.

Brazier-Hicks, M., Evans, K.M., Gershater, M.C., Puschmann, H., Steel, P.G., and Edwards, R. (2009). The C-Glycosylation of flavonoids in cereals. J Biol Chem **284,** 17926-17934.

Brockmoller, T., Ling, Z.H., Li, D.P., Gaquerel, E., Baldwin, I.T., and Xu, S.Q. (2017). *Nicotiana attenuata* Data Hub (NaDH): an integrative platform for exploring genomic, transcriptomic and metabolomic data in wild tobacco. Bmc Genomics **18**.

Campbell, J.A., Davies, G.J., Bulone, V., and Henrissat, B. (1997). A classification of nucleotide-diphospho-sugar glycosyltransferases based on amino acid sequence similarities. Biochem J **326,** 929-939.

Campbell, J.A., Davies, G.J., Bulone, V., and Henrissat, B. (1998). A classification of nucleotide-diphospho-sugar glycosyltransferases based on amino acid sequence similarities (vol 326, pg 929, 1997). Biochem J **329,** 719-719.

Caputi, L., Malnoy, M., Goremykin, V., Nikiforova, S., and Martens, S. (2012). A genome-wide phylogenetic reconstruction of family 1 UDP-glycosyltransferases revealed the expansion of the family during the adaptation of plants to life on land. Plant J **69,** 1030-1042.

Coutinho, P.M., Deleury, E., Davies, G.J., and Henrissat, B. (2003). An evolving hierarchical family classification for glycosyltransferases. J Mol Biol **328,** 307-317.

Gachon, C.M.M., Langlois-Meurinne, M., and Saindrenan, P. (2005). Plant secondary metabolism glycosyltransferases: the emerging functional analysis. Trends Plant Sci **10,** 542-549.

Gulati, J., Baldwin, I.T., and Gaquerel, E. (2014). The roots of plant defenses: integrative multivariate analyses uncover dynamic behaviors of gene and metabolic networks of roots elicited by leaf herbivory. Plant J **77,** 880-892.

Gulati, J., Kim, S.G., Baldwin, I.T., and Gaquerel, E. (2013). Deciphering herbivory-induced gene-to-metabolite dynamics in *Nicotiana attenuata* tissues using a multifactorial approach. Plant Physiol **162,** 1042-1059.

Huang, J., Pang, C.Y., Fan, S.L., Song, M.Z., Yu, J.W., Wei, H.L., Ma, Q.F., Li, L.B., Zhang, C., and Yu, S.X. (2015). Genome-wide analysis of the family 1 glycosyltransferases in cotton. Mol Genet Genomics **290,** 1805-1818.

Kim, S.G., Yon, F., Gaquerel, E., Gulati, J., and Baldwin, I.T. (2011). Tissue specific diurnal rhythms of metabolites and their regulation during herbivore attack in a native tobacco, *Nicotiana attenuata*. Plos One **6**.

Kubo, A., Arai, Y., Nagashima, S., and Yoshikawa, T. (2004). Alteration of sugar donor specificities of plant glycosyltransferases by a single point mutation. Arch Biochem Biophys **429,** 198-203.

Lairson, L.L., Henrissat, B., Davies, G.J., and Withers, S.G. (2008). Glycosyltransferases: Structures, functions, and mechanisms. Annu Rev Biochem **77,** 521-555.

Lee, H., and Raskin, I. (1999). Purification, cloning, and expression of a pathogen inducible UDP-glucose: Salicylic acid glucosyltransferase from tobacco. J Biol Chem **274,** 36637-36642.

Li, Y., Baldauf, S., Lim, E.K., and Bowles, D.J. (2001). Phylogenetic analysis of the UDP-glycosyltransferase multigene family of *Arabidopsis thaliana*. J Biol Chem **276,** 4338-4343.

Li, Y.J., Li, P., Wang, Y., Dong, R.R., Yu, H.M., and Hou, B.K. (2014). Genome-wide identification and phylogenetic analysis of Family-1 UDP glycosyltransferases in maize (*Zea mays*). Planta **239,** 1265-1279.

Lim, E.K., Higgins, G.S., Li, Y., and Bowles, D.J. (2003). Regioselectivity of glucosylation of caffeic acid by a UDP-glucose : glucosyltransferase is maintained in planta. Biochem J **373,** 987-992.

Lombard, V., Ramulu, H.G., Drula, E., Coutinho, P.M., and Henrissat, B. (2014). The carbohydrate-active enzymes database (CAZy) in 2013. Nucleic Acids Res **42,** D490-D495.

Mackenzie, P.I., Owens, I.S., Burchell, B., Bock, K.W., Bairoch, A., Belanger, A., FournelGigleux, S., Green, M., Hum, D.W., Iyanagi, T., Lancet, D., Louisot, P., Magdalou, J., Chowdhury, J.R., Ritter, J.K., Schachter, H., Tephly, T.R., Tipton, K.F., and Nebert, D.W. (1997). The UDP glycosyltransferase gene superfamily: Recommended nomenclature update based on evolutionary divergence. Pharmacogenetics **7,** 255-269.

Merken, H.M., and Beecher, G.R. (2000). Liquid chromatographic method for the separation and quantification of prominent flavonoid aglycones. J Chromatogr A **897,** 177-184.

Modolo, L.V., Blount, J.W., Achnine, L., Naoumkina, M.A., Wang, X.Q., and Dixon, R.A. (2007). A functional genomics approach to (iso)flavonoid glycosylation in the model legume *Medicago truncatula*. Plant Mol Biol **64,** 499-518.

Paquette, S., Moller, B.L., and Bak, S. (2003). On the origin of family 1 plant glycosyltransferases. Phytochemistry **62,** 399-413.

Rehman, H.M., Nawaz, M.A., Bao, L., Shah, Z.H., Lee, J.M., Ahmad, M.Q., Chung, G., and Yang, S.H. (2016). Genome-wide analysis of Family-1 UDP-glycosyltransferases in soybean confirms their abundance and varied expression during seed development. J Plant Physiol **206,** 87-97.

Ross, J., Li, Y., Lim, E.K., and Bowles, D.J. (2001). Higher plant glycosyltransferases. Genome Biol **2**.

Taguchi, G., Yazawa, T., Hayashida, N., and Okazaki, M. (2001). Molecular cloning and heterologous expression of novel glucosyltransferases from tobacco cultured cells

that have broad substrate specificity and are induced by salicylic acid and auxin. Eur J Biochem **268,** 4086-4094.

Taguchi, G., Nakamura, M., Hayashida, N., and Okazaki, M. (2003). Exogenously added naphthols induce three glucosyltransferases, and are accumulated as glucosides in tobacco cells. Plant Sci **164,** 231-240.

Vogt, T., and Jones, P. (2000). Glycosyltransferases in plant natural product synthesis: characterization of a supergene family. Trends Plant Sci **5,** 380-386.

Weadge, J.T.a.P., M.M. . (2000). Chemistry of Glycosyltransferases. Vol. 2. Wiley Encyclopedia of Chemical Biology **99,** 198-211.

Xu, S.Q., Brockmoller, T., Navarro-Quezada, A., Kuhl, H., Gase, K., Ling, Z.H., Zhou, W.W., Kreitzer, C., Stanke, M., Tang, H.B., Lyons, E., Pandey, P., Pandey, S.P., Timmermann, B., Gaquerel, E., and Baldwin, I.T. (2017). Wild tobacco genomes reveal the evolution of nicotine biosynthesis. P Natl Acad Sci USA **114,** 6133-6138

Yonekura-Sakakibara, K., and Hanada, K. (2011). An evolutionary view of functional diversity in family 1 glycosyltransferases. Plant J **66,** 182-193.

Yonekura-Sakakibara, K., Tohge, T., Niida, R., and Saito, K. (2007). Identification of a flavonol 7-O-rhamnosyltransferase gene determining flavonoid pattern in *Arabidopsis* by transcriptome coexpression analysis and reverse genetics. J Biol Chem **282,** 14932-14941.

Supplemental Methods 2

Disrupting HGL-DTG biosynthetic flux influences central metabolism

Quantitative profiling of 40 general and specialized metabolites (23 amino acids and biogenic amines, 4 small organic acids, 10 phenylpropanoids and derivatives and 4 sugars) using U(H)PLC-triple-quadrupole MS was performed on extracts from IR*ugt91t1*, IR*ugt74p5*, IR*ugt74p3/ugt74p5*, IR*ggpps* and WT leaves (Supplemental Figure 21, Supplemental Data Set 9). The IR*ugt91t1* leaf extracts of line A and B contained modest but significantly higher levels of α-keto-glutaric acid and Line B also contained higher levels of quercetin but not significantly higher levels of other flavonoids, such as quercetin-3-*O*-glucoside, quercetin-3-*O*-sophoroside, rutin or kaempferol-3-*O*-rutinoside. IR*ugt74p5* and IR*ugt74p3/ ugt74p5* leaf extracts exhibited higher levels of the biogenic amines, tyramine and tryptamine, of the amino acids L-tryptophan and L-cysteine, α-keto-glutaric acid and the phenylpropanoids caffeic acid, ferulic acid and synapylaldehyde. On the other hand, lower levels of glucuronic acid and shikimic acid were also detected. Interestingly, IR*ggpps* exhibited most changes in central metabolism, especially among amino acids. IR*ggpps* extracts contained higher levels in 11 amino acids (L-glutamine, L-histidine, L-lysine, L-proline, L-phenylalanine, L-threonine, L-tryptophan, L-tyrosine, L-valine, L-cysteine and L-methionine). Especially L-tryptophan (increased by more than 10-fold), L-histidine (increased by 6.6-fold) and L-glutamine (increased by 6-fold) levels were altered dramatically compared to those of WT. The biogenic amine tyramine, the phenylpropanoid derivatives, scopoletin and scopolin, and the small organic acids, succinic acid and α-keto-glutaric acid (138 × higher in IR*ggpps* than in WT) were increased as well. Furthermore L-alanine, L-aspartic acid, L-glutamic acid and shikimic acid were significantly reduced. Noteably, the concentration of L-aspartic acid was 13 times lower. Likewise, the free sugars glucose (7.6-fold), fructose (4.6-fold) and glucuronic acid (2-fold) were highly reduced in *IRggpps* as well.

Furthermore, we investigated the amount of the free prenyldiphosphates, GPP, FPP and GGPP, in IR*ggpps* compared to WT. We observed a significant reduction of GGPP (4.3-fold) and an increase in GPP (9.7-fold) and FPP (5.1-fold) in *IRggpps* (Supplemental Figure 22).

Leaves of plants impaired in HGL-DTG biosynthesis exhibit altered phytohormone levels

We observed a severe developmental phenotype in *N. attenuata* plants impaired in *UGT74P5* and *UGT74P3/UGT74P5* expression*, which* was similar to that reported for the phenotypes of plants abrogated in phytohormone signaling or biosynthesis (Ueguchi-Tanaka et al., 2005; Rodó et al., 2008). For this reason, we analyzed the phytohormone profiles of all lines with strong developmental and growth phenotypes. The quantitative profiling of 26 phytohormones and derivatives using U(H)PLC-triple-quadrupole-MS was performed on extracts from IR*ugt91t1*, IR*ugt74p5*, IR*ugt74p3/ugt74p5*, IR*ggpps* and wild type leaves (Supplemental Figure 21B, Supplemental Data Set 9).

First, our attention was directed to the gibberellin pathway, which is essential for many developmental processes in plants and therefore, when disturbed, could be responsible for the observed morphological alterations. We analyzed the most active gibberellins GA_1, GA_3, GA_4 and GA_7 (Schneider et al., 1989; Olszewski et al., 2002) for altered levels in leaf tissues. Of these compounds, we only detected GA_3 which was not significantly changed in any stable construct compared to wild type. Additionally we were able to measure large changes in GA_8, GA_{20} and GA_{51}. We showed that leaf extracts of IR*ugt74p5* line A and B and IR*ugt74p3/ugt74p5* contained higher gibberellin GA_{20} levels. The highest concentration was measured in the IR*ugt74p5* line A exhibiting 114 pmol/g FW. In IR*ugt74p5* line B, a concentration of 53.6 pmol/g FW was observed. The heterologous double construct with the most severe morphological phenotype, accumulated only 12.9 pmol/g FW, inconsistent with the hypothesis that GA_{20} would be responsible for these alterations. Neither IR*ugt91t1* nor IR*ggpps* plants showed detectable levels of GA_{20}. Additionally, GA_8 was 3.8 times (Line A) and 3.2 times (Line B) higher in IR*ugt74p5* compared to WT, but not detectable in IR*ugt74p3/ugt74p5.* In contrast, GA_{51} was only detectable in WT and both IR*ugt91t1* lines.

Further we investigated cytokinin levels which are essential for plant development (Mok and Mok, 1994). Our analysis of both IR*ugt91t1* lines revealed that they closely resembled WT plants in their cytokinin levels. Only dihydrozeatin (DHZ) could not be detected and line B had slightly increased levels of *cis*-zeatin (cZ). Both IR*ugt74p5* transformed lines showed increased levels of isopentenyladenine (IP), cZ and *cis*-zeatin-N7-glycoside (cZ7G). DHZ and dihydrozeatin-riboside (DHZR) were not detected and *cis*-zeatin-O-glucoside-riboside (cZROG) was reduced. Additionally *trans*-zeatin-riboside (tZR) was reduced in IR*ugt74p5* line A*.* IR*ugt74p3/ugt74p5* exhibited higher levels of cZ7G and lower levels of cZROG. In addition, DHZR could not be detected in the heterologous double construct. Interestingly, strong alterations were detected in IR*ggpps*. Leaf extracts of IR*ggpps* plants contained higher concentrations of the cytokinins cZ, cZR, DHZ, DHZR, czROG, *trans*-zeatin-N7-glycoside (tZ7G), cZ7G and dihydrozeatin-N7-glycoside (DHZ7G). The highest induction was observed for DHZR, which was increased 24-fold. The higher levels of cytokinins could explain the shorter stems and delayed flowering of IR*ggpps* plants.

Additionally to cytokinins and gibberellins, we analyzed jasmonate levels for all constructs. Interestingly strong alterations were detected in both IR*ugt74p5* lines. Particularly high concentrations of COOH-JA-Ile, OH-JA and JA-Ile were observed. The only difference between both IR*ugt74p5* lines was the high concentration of jasmonic acid (JA) in Line A, which could not be detected in Line B. Surprisingly IR*ugt74p3/ugt74p5* transformed plants showed no significant differences from the levels found in WT plants. However, COOH-JA-Ile, OH-JA and JA-Ile were clearly detectable in the heterologous double construct. In WT plants only JA-Ile and OH-JA-Ile could be observed. Furthermore, the phytohormones abscisic acid (ABA), indole acetic acid (IAA) and salicylic acid (SA) were measured in the leaf extracts. IR*ggpps* showed highly increased IAA levels (3.4-fold) and about half the levels in ABA. No significant differences in the concentration of SA were observed across all tested constructs.

The diverse morphological deformities of IR*ugt74p5* and IR*ugt74p3/ugt74p5* plants might be a nonspecific stress response triggered by the toxicity of 17-HGL (Bowles et al., 2005; Bowles et al., 2006; Mylona et al., 2008; Naoumkina et al., 2010; Itkin et al., 2011), which interferes with phytohormone homeostasis or the

accumulation of yet unknown compound classes. For example, *ugt74b1* mutants of *A. thaliana,* which are impaired in glucosinolate biosynthesis, display phenotypes of auxin overproduction, such as epinastic cotyledons and incomplete leaf vascularization (Grubb et al., 2004). Over-expression of a zeatin O-glucosylation gene in *Zea mays* leads to growth retardation and tassel-seed formation (Rodo et al., 2008). Furthermore, *gid1* mutants impaired in a soluble receptor for gibberellins in *Oryza sativa* displayed a severe dwarf phenotype with a loss of GA-responsiveness (Ueguchi-Tanaka et al., 2005). When we analyzed gibberellins in IR*ugt74p5* and IR*ugt74p3/ugt74p5* plants, we found no difference in gibberellic acid (GA_3) contents, but found high levels of GA_{20}, which is thought to be a mobile signal in the elongation of internodes and flower induction (Proebsting et al., 1992; Ross et al., 2001). The increased levels of GA_{20} is roughly consistent with a phenotype observed in the later developmental stages of IR*ugt74p5* and IR*ugt74p3/ugt74p5* plants as well as in their T_0-transformants, namely the prevalence of multiple internodes with stalled flower buds.

Reference:

Bowles, D., Isayenkova, J., Lim, E.K., and Poppenberger, B. (2005). Glycosyltransferases: managers of small molecules. Curr Opin Plant Biol **8,** 254-263.

Bowles, D., Lim, E.K., Poppenberger, B., and Vaistij, F.E. (2006). Glycosyltransferases of lipophilic small molecules. Annu Rev Plant Biol **57,** 567-597.

Grubb, C.D., Zipp, B.J., Ludwig-Muller, J., Masuno, M.N., Molinski, T.F., and Abel, S. (2004). *Arabidopsis* glucosyltransferase UGT74B1 functions in glucosinolate biosynthesis and auxin homeostasis. Plant J **40,** 893-908.

Itkin, M., Rogachev, I., Alkan, N., Rosenberg, T., Malitsky, S., Masini, L., Meir, S., Iijima, Y., Aoki, K., de Vos, R., Prusky, D., Burdman, S., Beekwilder, J., and Aharoni, A. (2011). GLYCOALKALOID METABOLISM1 Is Required for Steroidal Alkaloid Glycosylation and Prevention of Phytotoxicity in Tomato. Plant Cell **23,** 4507-4525.

Mok, M.C., and Mok, D.W.S. (1994). Cytokinins: Chemistry, Activity, and Function. CRC Press Inc.

Mylona, P., Owatworakit, A., Papadopoulou, K., Jenner, H., Qin, B., Findlay, K., Hill, L., Qi, X., Bakht, S., Melton, R., and Osbourn, A. (2008). Sad3 and Sad4 are required for saponin biosynthesis and root development in oat. Plant Cell **20,** 201-212.

Naoumkina, M.A., Modolo, L.V., Huhman, D.V., Urbanczyk-Wochniak, E., Tang, Y.H., Sumner, L.W., and Dixon, R.A. (2010). Genomic and Coexpression Analyses Predict Multiple Genes Involved in Triterpene Saponin Biosynthesis in *Medicago truncatula*. Plant Cell **22,** 850-866.

Olszewski, N., Sun, T.P., and Gubler, F. (2002). Gibberellin signaling: Biosynthesis, catabolism, and response pathways. Plant Cell **14,** S61-S80.

Proebsting, W.M., Hedden, P., Lewis, M.J., Croker, S.J., and Proebsting, L.N. (1992). Gibberellin Concentration and Transport in Genetic Lines of Pea - Effects of Grafting. Plant Physiol **100,** 1354-1360.

Rodo, A.P., Brugiere, N., Vankova, R., Malbeck, J., Olson, J.M., Haines, S.C., Martin, R.C., Habben, J.E., Mok, D.W.S., and Mok, M.C. (2008). Over-expression of a zeatin O-glucosylation gene in maize leads to growth retardation and tasselseed formation. J Exp Bot **59,** 2673-2686.

Ross, J., Li, Y., Lim, E.K., and Bowles, D.J. (2001). Higher plant glycosyltransferases. Genome Biol **2**.

Schneider, G., Sembdner, G., Schreiber, K., and Phinney, B.O. (1989). Partial Synthesis of Some Physiologically Relevant Gibberellin Glucosyl Conjugates. Tetrahedron **45,** 1355-1364.

Ueguchi-Tanaka, M., Ashikari, M., Nakajima, M., Itoh, H., Katoh, E., Kobayashi, M., Chow, T.Y., Hsing, Y.I.C., Kitano, H., Yamaguchi, I., and Matsuoka, M. (2005). GIBBERELLIN INSENSITIVE DWARF1 encodes a soluble receptor for gibberellin. Nature **437,** 693-698.
