# Supplementary material for: Specific decorations of 17-hydroxygeranyllinalool diterpene glycosides solve the autotoxicity problem of chemical defense in Nicotiana attenuata
Source: Plant Cell. 2021 Feb 9;33(5):1748–70. doi: 10.1093/plcell/koab048 (PMC8254506; doi:10.1093/plcell/koab048)
Supplement: koab048_Supplementary_Data [file koab048_supplementary_data.zip › tpc.00165.2020-s13.pdf]

Specific decorations of 17-hydroxygeranyllinalool diterpene glycosides solve the autotoxicity problem of chemical defense in *Nicotiana attenuata*

Sven Heiling, Lucas Cortes Llorca, Jiancai Li, Klaus Gase, Axel Schmidt, Martin Schäfer, Bernd Schneider, Rayko Halitschke, Emmanuel Gaquerel, and Ian Thomas Baldwin

Corresponding author: Ian T. Baldwin baldwin@ice.mpg.de and Emmanuel Gaquerel emmanuel.gaquerel@ibmp-cnrs.unistra.fr.

Review timeline:

**TPC2017-RA-00817**	Submission received:	Oct. 25, 2017
	1st Decision:	Dec. 18, 2017 *manuscript declined*
**TPC2020-RA-00165**	Submission received:	Feb. 28, 2020
	1st Decision:	Apr. 27, 2020 *accept with minor revision*
**TPC2020-RA-00165R1**	1st Revision received:	Jan. 22, 2021
	2nd Decision:	Jan. 22, 2021 *acceptance pending, sent to science editor*
	Final acceptance:	Feb. 3, 2021

REPORT: (The report shows the major requests for revision and author responses. Minor comments for revision and miscellaneous correspondence are not included. The original format may not be reflected in this compilation, but the reviewer comments and author responses are not edited, except to correct minor typographical or spelling errors that could be a source of ambiguity.)

TPC2017-RA-00817 1st Editorial decision – *declined* **Dec. 18, 2017**

As you will see when you read their comments, the reviewers had significant concerns about a number of issues in your paper, including the rigor of the biochemical characterization of the heterologously expressed enzymes, the breadth of enzymes and substrates used in the characterization, and the mass spectral identification of the compounds in question. Some figures are difficult to read and lack statistics. Perhaps more importantly though is the size of this manuscript and the uneven impact of the data reported. Several reviewers and editorial board members commented that the manuscript is overly ambitious and its lack of focus in parts detracted from the impact of the stronger data it includes. As a result, the manuscript is long and somewhat cumbersome, becoming too descriptive and overly speculative in parts. For example, the data reported on lines 494-578 and 656-747 are only correlational and could be deleted to enhance the focus of the manuscript. The Discussion from lines 861-880 is redundant with similar text in the Results. Although it is not our intent to dictate publication strategy, it would seem that this manuscript would benefit from being split into two papers, each of which could be more focused.

-- Reviewer comments:

Reviewer #1 (Comments for the Author):

The manuscript by Heiling et al. reports on comprehensive multi-omic analyses to identify and functionally characterize candidate genes with putative involvement in the biosynthesis of 17-hydroxygeranyllinalool (HGL) glycosides in Nicotiana. The topic is very interesting and has significant potential, but various aspects of the experimental design, data analysis and presentation need to be revised.

The narrative and figure legends contain a large number of typographical, grammatical, punctuation and character spacing mistakes and inconsistencies. This leaves the reader with the impression of a hastily assembled manuscript, which is highly annoying when reviewing for a top journal. Only a few examples can be given here but similar problems can be found throughout the manuscript.

Narrative examples:
Line 1: "autoxicity" should be "autotoxicity".
Line 41: "In vitro, enzyme assays" should be "In vitro enzyme assays"
Line 66: (Yamamoto et al., 1990; Hollman et al., 1996); citations need to be consistent (and in accordance with Plant Cell author guidelines) regarding italicization of "et al.". The same applies to the usage of a comma after "et al." or not.
Line 95: "and even phytohormones ." (there should be no space before period).
Line 99: "2003). However, " (there needs to be a space after period)

Figure examples: Figure 2 (legend): "ortostichous" should be "orthostichous".
Figures 3A, 4A and 6A: "intermediate with one glucose moieties" should be "intermediate with one glucose moiety".
Maybe I missed the explanation, but I do not understand why the authors do not use the common UGT nomenclature for NaUDP-GT2 and NoUDP-GT2.

Figure 2 represents a bioinformatic analysis of previously published data and a description of the microarray experiment used for data analysis is provided in the Methods section. It would be more appropriate to present these analyses (and corresponding experimental details) in a Supplemental Methods and Data File. This would allow the authors to present the case for the selection of candidates more convincingly, for example by providing Pearson correlation coefficients for reciprocal analyses of UGTs with both GGPPS and GLS (only GGPPS correlation included in supplemental data) (heat maps could be employed for visualization). The phylogenetic analysis should include all *N. attenuata* UGTs and all (or a representative selection) of functionally characterized UGTs from other plants in one figure. The phylogenetic tree presented in Figure 2A does not allow the reader to associate a position on the tree with the unique identifiers used in supplemental figures and tables.

The biochemical characterization of UGTs is insufficient. Recombinant proteins need to be purified to ensure that the observed activity stems from the protein of interest. Figure 5 shows a conversion of 17-geranyllinalool to several, likely glycosylated, products. However, other potential substrates were not tested, which means that there might be a preference for an entirely different substrate. Kinetic data (Km, Vmax, Kcat) are essential as well to characterize a novel activity. The discussion of the potential function of UDP-GT2 (may work only on malonylated substrates) is unconvincing if the scheme in Figure 1 should be correct. This issue needs serious consideration and thought (plus experimentation) before a plausible hypothesis can be presented.

From a technical point of view, the chemical analyses of transgenic plants are very thorough and impressive. However, the results are difficult to interpret. Why would the pTVUGT74P3 and pTVUDP-GT2 transgenic plants have an essentially identical diterpene glycoside profiles when UGT74P3 has diterpenoid glycosylating activity while UDP-GT2 does not? Is it conceivable that the preferred substrate of either or both enzymes is different from the one tested here, and the observed profiles in silenced plants reflect secondary effects of a different metabolic impairment. This might also explain the pleiotropic effects on the levels of many other metabolites.

When considering the incomplete and unconvincing in vitro biochemical data, the selection of the candidate genes may have been too narrow. There are several other candidates that are closely related, both in terms of their sequence characteristics and general gene expression patterns. It is conceivable that the authors may have missed 'better' candidate UGTs. The fact that UGT91T1 clusters with enzymes known to be involved in flavonoid glycoside biosynthesis (rather than those known to be involved in diterpenoid glycoside biosynthesis) indicates that the utility of the phylogenetic analysis may be limited.

The authors need to be careful to not equate correlation with causation. In silenced plants, the accumulation of glycosylated diterpenoids correlates with changes in the quantities of many other metabolites. However, there is no direct evidence that this observation is based on a causative relationship. It is not unlikely that, because the transgenic plants are affected in various aspects of their development, the observed metabolite patterns are an indicator of unspecific stress responses. Interestingly, the most severe effects were observed with plants silenced in the expression of UDP-GT2, which might be another indication that its function is unrelated to diterpenoid glycosylation.

The discussion of how GGPP may regulate several genes putatively involved in diterpenoid glycosylation is interesting but highly speculative. Much more genetic evidence would be required to justify the inclusion of two figures to visualize this hypothesis.

Reviewer #2 (Comments for the Author):
General Comments: Heilling and colleagues report on the identification and characterization of multiple novel UDP glucosyl transferases associated with 17-HGL biosynthesis in Nicotiana. The authors use gene-to-gene expression and gene to metabolite correlations to identify potential UGT candidates. The authors then provided rigorous biochemical and molecular evidence of function using in vitro biochemical assays and both transient and stable gene silencing. The authors further delve into the cytotoxic nature of 17-HGL and glycosylation as a mechanism of managing cytotoxicity. Overall, this is a very comprehensive and high quality report. This reviewer is fairly enthusiastic about the article. The only major revision suggested would be to include a Supplemental table of all the gene expression time course data. Several minor editorial suggestion follows in the specific comments. Specific Comments:
Line 95; remove erroneous space before period.
Ln 143: What about the role of JA-ILE in regulating HGL-DTGs?
Ln 233: I would encourage the authors to also include a comprehensive table of all relative gene expression vs. time in addition to the UGTs reported in Supp. Fig 4.
Ln 243: suggest you use term 'we putatively identified UGTs' as the correlations at this point are insufficient to prove function...noting that you actually prove later.
Ln 307-309: The authors cite a previous report on dereplication and metabolite identification, but I would strongly encourage the authors to briefly describe their methods and rigor for metabolite identification within this manuscript and how the align with the community standards reported in Creek et al, 2014 and Sumner et al, 2007.
Lns 356-357: Again, please define the rigor used to identify the HGL-DTGs relative to community standards.
Ln399: please define rigor for lyciumoside I and other di-glucosides. Did this include matching to authentic standards Rt and MS/MS?
Lns 416-417: what was the root phenotype of the transformed plants?
Ln430: why use a heterozygous double construct? Was the homozygous lethal?
Lns 452-460: Again, please define the rigor used to identify the HGL-DTGs relative to community standards; especially the malonylated compounds, which I doubt are commercially available.
Lns 466-468: I would classify these as putatively identified unless further evidence provided.
Lns 610: The authors report deviations as RSD, or relative standard deviations which is usually provided as a percentage, but the data lack a percentage unit notation thus a suspect these are standard deviations; i.e. not relative STD.
Lns700-702. Malonyl conjugates decompose rather easily and at elevated temperatures. Thus, you might want to briefly note extraction conditions in text here.
Lns913-915. The neutral loss of rhamnose would be the same for other methyl pentoses. Thus, how did you ensure specificity for only the rhamnose ids?
Ln958: authors state '...compounds further upstream...' is this accurate or do you mean downstream as these are all tri and tetra saccharides at the end of the pathway?
Lns 985-90: Why use just 17_HGL substrates? You can show greater selectivity by using a hydrolyzed series of endogenous diterpenoids.
Lns994-997: Did you try other group L substrates with UDP-GT2 such as IAA, gypsongenic acid, steviol, mogroside, etc. as these would also be likely substrates if no activity for DTGs.
Lns 1205-1206: What is the purpose of using 'liquid smoke' on the seeds?
Ln 1392: insert word 'peaks' to read '...and plant analyte peaks was performed...'
Lns1456-throughout: Be consistent with instrumental abbreviations; i.e. UHR-Q-TO-MS and UHR-Q-TOFMS.
Figure 5: Can you identify other peaks in the substrate mixture beyond 17-HGL?

Reviewer #3 (Comments for the Author):
The manuscript describes an extensive body of work that investigated at multiple levels the biosynthesis of diterpene glycosides, major anti-herbivore defense molecules produced by N. attenuata and other species. Extensive screening of GTs for defense related induction of gene expression resulted in the identification of 3 GTs that were shown in vitro and in vivo to act in the diterpene pathway either in rhamnosylation or glucosylation of the 17-hydroxygeranyllinalool (17-HGL). Plants altered in glycosylation were severally affected in growth and the authors show elegantly that toxicity is likely through the accumulation of the aglycone 17-HDL in the plant tissue. The phenotyping of VIGS and stably transformed plants was performed by using advanced metabolite profiling methods that were most helpful in understanding feedback loops in the pathway and the likely source of phytotoxicity. Finally through integrating gene expression and metabolite levels associated with the pathway the authors could point or suggest points of feed -back and -forward regulation.

The research is impressive in its depth (especially at the metabolic level) and deals with a significant class of secondary metabolites that was hardly investigated to date. One of the major interests in the study is the issue of phytotoxicity; through single and combinations of silenced lines the authors dissected the metabolite in the pathway that imparts toxicity to the cell. This is in line with studies performed on other sec. metabolites classes showing the aglycones are phytotoxic and are glycosylated to avoid self-toxicity.

Major comments:
1. The main text is way too long, which makes the reading complicated. It includes some parts that are elaborated in a way that is not proportional to the significance of the finding. One main example is hormone profiling, which is (as always) very difficult to make clear sense of and does not add much to the overall picture. Not only the Results but also the Discussion section are too extensive; phylogenetic of the GTs is not so significant to the story, why does it need a whole page? Thus, the entire paper should be re-examined for what is a real must to have in the main text and largely compacted in some sections.
2. Although the aglycone 17-HDL is the likely cause of toxicity, the lack of knowledge regarding the primary target of this molecule is somewhat disappointing. While I understand the difficulty in identifying such target, I would at least have some suggestion introduced to the manuscript of what could it be.
3. The authors could not identify the function of UDP-GT2; did they try to conduct activity assays in a combination of the other GTs (i.e. in the same tube).
4. The authors lab is a world leader in plant herbivory research; I would expect that stable transgenic plants generated in the course of this study will be used for herbivory assays. If such experiments were conducted, it is very much worthy of introducing them to this manuscript, certainly instead of several experiments that did not provide a real significant conclusion that are now in the main text.
5. The phenotypes observed could be possibly recovered by the application of hormones or hormone biosynthesis or signaling inhibitors; did the authors try to complement the phenotypes or make them more severe by application of such substances?
6. As many other reports on the discovery of enzymes in secondary metabolism, also here the reader needs to understand better the relevance of the findings in this specific chemical class and at least understand to a certain extent how big this class of sec. metabolites, what families of plant are known to produce it and more on its activity in plant-herbivore interactions.

TPC2020-RA-00165 Submission received Feb. 28, 2020

AUTHOR RESPONSES: We would like to thank both the reviewers and the editor for their careful evaluation of the manuscript. We hereby resubmit a heavily revised manuscript "Specific decorations of 17-hydroxygeranyllinalool diterpene glycosides solve the autotoxicity problem of chemical defense in *Nicotiana attenuata*" addressing the concerns raised during the first review process.

As recommended by the reviewers, we streamlined the manuscript extensively and focused on the major interest of the study: the role of glycosylation reactions in avoiding the phytotoxicity problem of 17-HGL-based anti-herbivore defenses. Hence, we have removed overly speculative discussion sections of the manuscript and made the text

more compact and focused by moving several paragraphs associated to supplemental data into the supplemental materials.

<u>We addressed the major comments of the reviewers as described below:</u>

As suggested by reviewer 3, we report in the Introduction and Discussion sections of the manuscript on two-component defense systems in plants and additional examples of biochemical mechanisms evolved by plants to minimize autotoxicity by certain specialized metabolite classes. Additionally, we include new quantitative data obtained from performance assays with *M. sexta* larvae on the different transgenic lines generated in the context of this study (Figure 9) and discuss the activity of HGL-DTGs in plant-herbivore interactions. As mentioned above, we moved the extensive discussion of the UGT phylogeny as well as the detailed phytohormone profiles from the main text to the supplemental material.

As suggested by reviewer 1, we conducted additional enzyme assays with purified recombinant proteins to further consolidate that the observed glycosylation activity stems from the targeted *Nicotiana* UGT proteins of interest. Further, we conducted the enzyme assay for UDP-GT2 in combination with both NaUGT74P3 and NoUGT74P4. These new data are included in the manuscript, and shown in Figure 4.

We did not conduct further tests with other substrates than 17-HGL, and hence cannot provide kinetic data (Km, Vmax, Kcat) for the individual enzymes. Without purified substrates, these kinetic parameters are challenging to calculate rigorously, and as the emphasis of the work is focused on the *in planta* functions of these glycosyltransferases in solving the 'toxic waste dump" problem of 17-HGL-DTG biosynthesis, rather than being biochemical characterization of the structure-activity relationships exhibited by these UGTs, we felt that this data was outside the scope of the study.

By using genetically manipulated plants (IR*ggpps*), which do not produce the HGL-DTG precursor and in which the phytotoxic effect of UGT silencing is abolished, we demonstrate that the observed developmental alterations are directly linked to the glycosylation of the HGL-DTG precursor.

We also addressed the concerns of reviewer 1 regarding the essentially identical HGL-DTG profiles measured in leaves of pTVUGT74P3 and pTVUDP-GT2 transgenic plants. We inserted additional expression data (Supplemental Figure 8), in order to clarify that the UGT74P3 and UDP-GT2 are reciprocally co-silenced in pTVUGT74P3 and pTVUDP-GT2 transgenic plants, thereby generating overlapping disruptions of their glucosylation reactions in HGL-DTG metabolism.

We agree with reviewer 1's concern of directly extrapolating enzymatic functions for the manipulated genes solely from the deregulations detected in metabolic profiles in the different transgenic backgrounds. As mentioned above, we provide evidence from purified protein-based enzyme assays regarding the biochemical function of UGT74P3 and UGT74P4 as UDP-glucosyltransferases in HGL-DTG synthesis. Additionally, we were able to abrogate the strong morphological alterations that result from silencing *UGT74P3* and *UDP-GT2* (Figure 7), without the application of hormones or signalling inhibitors, as suggested by reviewer 3. Application of gibberellins did not rescue the morphological phenotype of IR*ugt74p3* and IR*udp-gt2* plants and as such was not included into the manuscript. More importantly, we provide evidence that an excess of 17-HGL triggers cell necrosis in *N. attenuata* leaves and show that 17-HGL is only detected in transiently- or stably-silenced plants exhibiting these severe phenotypes.

We would like to point out that the most severe morphological alterations were observed in plants silenced in the expression of UGT74P3. The phenotypic characteristics of IR*ugt74p3* transformants could not be transferred to the T_2 generation, as T_1 plants aborted most flower buds early during development and did not produce fertile flowers. This is consistent with the work of Li and colleagues (Li et al., 2018), who show that the disturbances in the uniform malonylation patterns of HGL-DTGs leads to a specific reduction in the floral style lengths of *N. attenuata* flowers and therefore alter flower fertility.

Another major comment regarded the levels of identifications of the HGL-DTGs, which were used in this study. We agree with reviewer 2 that the criteria for metabolite identification should follow community standards and be transparently presented. We revised the supplemental mass spectrometric data tables and included the identification levels (1-4) based on the criteria detailed in (Sumner et al., 2007), to ensure that the rigor of identification is clear for every HGL-DTG. We also added a paragraph in the Method section describing the dereplication process used in the study. HGL-DTGs have been studied in our group for more than two decades and all compounds with an identification level of 1 were purified, characterized by NMR, injected as authentic standards into the mass spectrometers and showed perfect matches based on retention times and MS/MS fragmentation. In

addition, all NMR data exclusively show rhamnose as the methyl pentose associated with HGL-DTGs in *N. attenuata* and *N. obtusifolia*. After the loss of all malonyl decorations in an alkaline pH environment, only the few well-described non-malonylated HGL-DTGs remain. We agree with reviewer 2 that the identification of rhamnose alone based solely on MS/MS data is not possible; but this is not what we did.

<u>We addressed the minor comments of the reviewers.</u>

As recommended by reviewer 1, we now report, in the supplemental data (Supplemental Table 5a/b), Pearson correlation coefficients calculated from reciprocal analyses between the targeted UGTs and both GGPPS and GLS.

We addressed the comment of reviewer 1 to streamline the manuscript and moved the complete phylogenetic characterization of the UGTs to the Supplemental materials. Additionally, we revised the phylogenetic tree of the UGTs in *N. attenuata* (Supplemental Figure 4A) to allow the reader to associate a position on the tree with the unique identifiers used in the other supplemental figures and tables. We did not include another phylogenetic analysis of all UGTs in *N. attenuata* with all or a representative selection of functionally characterized UGTs from other plants, as this would be too complex and visually incomprehensible. Furthermore, we agree with reviewer 1 and discuss in the manuscript that the phylogenetic analysis of UDP-rhamnosyl transferases (UDP-RTs) may be limited due to the lack of sufficiently functionally characterized UDP-RTs.

Reviewer 3 asked about the existence of possible metabolic gene clusters for the 17-HGL-DTG pathway. Current assemblies of *N. attenuata* and *N. obtusifolia* genomes do not allow us to rigorously explore the possible existence of such a gene cluster.

As noted by reviewer 1, we did not use the common UGT nomenclature for NaUDP-GT2 and NoUDP-GT2, because we were not able to show a clear UDP-glycosyltransferase function.

As recommended by reviewer 2, we included a comprehensive table of all relative gene expressions vs. time (Supplemental Data 1) in addition to the UGTs reported in Supp. Fig 5.

TPC2020-RA-00165 1st Editorial decision – *accept with minor revision* **Apr. 27, 2020**

The three reviewers have provided thoughtful and careful reviews and have not asked for any further experiments. We would ask however, that in a revised manuscript, you address all of their comments, all of which are relatively straightforward editorial changes. In particular, Reviewer 1 asks you to pay attention to and reduce the text in the Introduction on lines 152-180 that are largely a reiteration of the Abstract and provide a rather lengthy synopsis of the results to be presented. Similarly, we would point out that lines 550-572 and 585-654 in the Discussion are largely a retelling of the results and could be reduced or preferably eliminated without loss of impact. Also, in your Response to Reviewers Comments from the last version of the manuscript you state "As noted by reviewer 1, we did not use the common UGT nomenclature for NaUDP-GT2 and NoUDP-GT2, because we were not able to show a clear UDP glycosyltransferase function." Reviewer 2 is not persuaded by this argument and asks for the use of a more systematic naming of the UGTs for the sake of clarity and we agree that this would be of benefit for the broad audience of The Plant Cell.

As noted by Reviewer #1, you should recolor the heat maps to a yellow/blue/magenta spectrum to make these interpretable to those with color vision deficiencies. We are in the process of updating our instructions to authors to address this. The problematic heat maps are shown in at least three figure panels (2B, 3B, & 5B), plus Supp. Figure 4A, Supp Figure 15B, Supp Figure 21. If it would be helpful to see what those with color vision deficiencies see, there is a free simulator: http://colororacle.org/ with an article showing the problematic colors: http://colororacle.org/colororacle/resources/2007_JennyKelso_ColorDesign_lores.pdf. In addition, our instructions to authors describes that large-scale datasets should be made permanently available via submission to public repositories, and this should apply to your metabolomics data set. Please deposit these data and provide the accession numbers in the revised manuscript.

-- Reviewer comments:

Reviewer #1 (Comments for the Author):
I appreciated the efforts made by Heiling et al. to address reviewer concerns. The resubmitted manuscript is a

substantial improvement over the original submission at all levels (study design, data analysis, presentation, and narrative). The topic is interesting, the results are novel and the conclusions constitute a compelling advance. I had only a few minor suggestions for improvements:

Line 96 and following: "Another diverse compound class whose members are often glycosylated, associated with phytotoxic activities (Macias et al., 2008) and have potent anti-herbivore resistance/deterrence effects, is that of diterpene glycosides (DTGs)". Please reword because DTGs are by definition glycosylated. Line 144 and following: "all malonyl moieties of HGL-DTGs are rapidly lost when leaves are ingested by *M. sexta* larvae, suggesting that the malonylation of HGL-DTGs does not play a central role in anti-herbivore defense (Poreddy et al., 2015). Interestingly, disruption of the uniform malonylation patterns of HGL-DTGs leads to a specific reduction in the floral style lengths of *N. attenuata* flowers (Li et al., 2018). This shows that specific decorations of a plant's specialized metabolites can play a crucial, but poorly understood, role in plant development".

It is certainly true that the functional role of malonylation reactions is not fully understood. However, there is growing evidence that malonylation may favor the transport of metabolites into the vacuole and thus facilitating storage (Taguchi et al. (2010) Plant J 63, 1031 and other publications). I would encourage the authors to consider this interpretation of malonylation in the context of DTGs.

Line 152-180: Close to 30 lines of the Introduction are used to describe results obtained as part of this study. This is a repetition of the content of the Abstract and should be shortened considerably.

The MS/MS data presented in Supplemental Materials are an integral part of the identification strategy for DTGs and are as such of broader value. I am not sure about requirements at Plant Cell but the Metabolomics Standards Initiative strongly suggests the deposition of MS and MS/MS spectra in community databases (MassBank or similar).

Comment on figure design:
Several of the figures include heatmaps with a red - yellow- green color palette. Once again, I am not sure about Plant Cell policies, but I would ask the authors to consider a different coloring (such as red - white - blue or other). I asked my color-blind son (who has a scientific degree) to look at the figures and he could not interpret the content of the heatmaps.

Reviewer #4 (Comments for the Author):
This revised manuscript describes further studies of 17-hydroxygeranyllinalool diterpene glycosides (HGL-DTGs) in Nicotiana, specifically focused on elucidation of the relevant glycosyltransferases (UGTs) and, more impactfully, the physiological rationale for the resulting glycosylation. The work is solid, although the results are somewhat inconclusive in terms of identifying the exact biosynthetic roles of the UGTs, insight is provided regarding physiological role, which is clearly indicated to be chemical sequestration of the phytotoxic aglycone 17-hydroxygeranyllinalool (HGT). Note that this is not detoxification per se, as claimed by the authors, but rather chemical sequestration, which is a perhaps subtle but important distinction. Nevertheless, the resulting HGT-DGTs exhibit obvious toxicity towards *Manduca sexta*, and it is a bit misleading (not to mention confusing) to term it detoxification. The manuscript can be hard to follow at times for other reasons, particularly the results from the reported gene silencing, which were not entirely conclusive. While it is not immediately evident how this could be fully clarified, it should be noted that the use of different nomenclature for what seem to be orthologous, and are obviously homologous, enzymes increases the difficulty of following the results. While an argument is made in the response for keeping the current names intact, this seems to be more for the authors convenience rather than any truly valid rationale, and really should be reconsidered. For example, UGT91T1 and the orthologous (but unfortunately entirely distinctly named) UDP-RT1), as well as the orthologous pairs UGT74P3/P4 from *N. attenuata* and *N. obtusifolia* and unfortunately named Na/NoUDP-GT2. This could be significantly improved by normalizing the nomenclature - e.g., all of these almost certainly could easily be fit into the UGT family nomenclature like UGT74P3/P4, which presumably would make it easier to distinguish orthologous pairs as well as distinct paralogs. In addition, it might be helpful if it was noted (perhaps in the Introduction - line 165?) that rhamnosylation requires prior glucosylation. Other helpful changes would include clarifying the transitions between descriptions of the various enzymes (e.g., in the introduction - line 165 - transitioning from apparent rhamnosyltransferases to glucosyltransferases; note that it should be clarified

in the first sentence describing UGT91T1 and UDP-RT1 that the comparison was of empty vector to silenced lines). Finally, as a minor correction the reaction catalyzed by geranyllinalool synthases is not hydroxylation of GGPP but rather indirect hydrolysis of the allylic diphosphate ester, thus, explaining the loss of the diphosphate and double-bond isomerization. Despite these issues, the work reported here is significant in providing strong evidence for not only a role for glycosylation as a chemical sequestration mechanism to alleviate the auto/phyto-toxicity of the aglycone HGT, but also the differential effect of glucosylation versus rhamnosylation on herbivore deterrence.

Reviewer #6 (Comments for the Author):
This paper describes a very impressive and detailed characterization of UDP-sugar dependent glycosylation of 17-hydroxygeranyllinalool in *Nicotiana attenuata*. Beyond the overall gene discovery, molecular and biochemical characterization of three genes and two enzymes, the paper explores the role of diterpene glycosylation in the control of auto-toxicity of this diterpene defense metabolite system. The paper is a revised submission. I was not one of the original reviewers, but I did read the response to the original reviews. I believe the authors addressed the major concerns of the original reviews. I only have a few relatively minor comments that can be addressed with editorial revisions and perhaps an additional reference.

1. While UGT74P3 and UGT74P4 enzymes were functionally characterized using in vitro assays, characterization of UGT91T1 did not include such assays. This reviewer appreciates the difficulty of characterizing rhamnosyltransferases, since UDP-rhamnose (UDP-Rha) is - to the best of my knowledge - not commercially available. As the authors mentioned on page 19 (lines 581-584), a few labs have been successful in overcoming the issue of producing UDP-Rha. A recent study by Irmisch et al. (2018) The Plant Cell 30: 1864-1886 should perhaps be included as a reference for an efficient enzymatic system to produce UDP-Rha and its use for UGT characterization. Although I am not suggesting additional experiments for this present paper, the authors could contact Irmisch and co-workers for assistance with enzymatic UDP-Rha synthesis if needed for future work.
2. Page 8, line 227-236: The authors correctly highlighted that few rhamosyltransferases have been characterized. I would therefore suggest that the flavonol rhamnosyltransferase described by Irimsch et al. (2018) (see above) be included in the comparison described here and illustrated in Supplemental Figure S6.
3. P17, line 519-529. It says "We identified three novel UGTs ...". The following lines only mention two, UGT74P3 and UGT74P4. The third UGT, UGT91T1, should also find mention in this paragraph. Again, this is a very impressive paper.

TPC2020-RA-00165R1 1st Revision received Jan. 22, 2021

Reviewer comments on previous submission and **author responses**:

Reviewer #1

I appreciated the efforts made by Heiling et al. to address reviewer concerns. The resubmitted manuscript is a substantial improvement over the original submission at all levels (study design, data analysis, presentation, and narrative). The topic is interesting, the results are novel and the conclusions constitute a compelling advance. I had only a few minor suggestions for improvements:

Point 1. Line 96 and following:
"Another diverse compound class whose members are often glycosylated, associated with phytotoxic activities (Macias et al., 2008) and have potent anti-herbivore resistance/deterrence effects, is that of diterpene glycosides (DTGs)". Please reword because DTGs are by definition glycosylated.

RESPONSE: This is indeed a good point. Thanks for pointing this out. This sentence has been re-written.

Point 2. Line 144 and following: "all malonyl moieties of HGL-DTGs are rapidly lost when leaves are ingested by *M. sexta* larvae, suggesting that the malonylation of HGL-DTGs does not play a central role in anti-herbivore defense (Poreddy et al., 2015). Interestingly, disruption of the uniform malonylation patterns of HGL-DTGs leads to a specific reduction in the floral style lengths of *N. attenuata* flowers (Li et al., 2018). This shows that specific decorations of a plant's specialized metabolites can play a crucial, but poorly understood, role in plant development".

It is certainly true that the functional role of malonylation reactions is not fully understood. However, there is growing evidence that malonylation may favor the transport of metabolites into the vacuole and thus facilitating storage (Taguchi et al. (2010) Plant J 63, 1031 and other publications). I would encourage the authors to consider this interpretation of malonylation in the context of DTGs.

RESPONSE: Thanks for suggesting this alternative functional role for malonylation. We now mention the functional role of DTG malonylation in the regulation of intra-cellular transport and vacuolar storage.

Point 3. Line 152-180: Close to 30 lines of the Introduction are used to describe results obtained as part of this study. This is a repetition of the content of the Abstract and should be shortened considerably. The MS/MS data presented in Supplemental Materials are an integral part of the identification strategy for DTGs and are as such of broader value. I am not sure about requirements at Plant Cell but the Metabolomics Standards Initiative strongly suggests the deposition of MS and MS/MS spectra in community databases (MassBank or similar).

RESPONSE: We have re-written and streamlined the Introduction to reduce redundancies with the Abstract and Result sections. MS metabolomics and annotations have been submitted to the Metabolights database hosted by EMBL-EBI, which is a reference database for exhaustive data sharing and reporting in the field of metabolomics. The accession number is MTBLS1819. Reference to this publicly available dataset is now made in the manuscript. The submission is currently under curation at Metabolights and will be directly accessible. Additionally, we want to emphasize that our supplemental data provide additional in-depth reporting on MS/MS collected as part of this study.

Point 4. Several of the figures include heatmaps with a red - yellow- green color palette. Once again, I am not sure about Plant Cell policies, but I would ask the authors to consider a different coloring (such as red - white - blue or other). I asked my color-blind son (who has a scientific degree) to look at the figures and he could not interpret the content of the heatmaps.

RESPONSE: Thanks for this important point regarding the interpretability of data visualizations. We have changed the color usage in the problematic heatmaps in Figure 2, 3 and 5 as well as in Supplemental Figure 4, 15 and 21. To take into consideration colour vision deficiencies, we now use yellow-to-blue color gradients.

Reviewer #4:

Point 1. This revised manuscript describes further studies of 17-hydroxygeranyllinalool diterpene glycosides (HGL-DTGs) in Nicotiana, specifically focused on elucidation of the relevant glycosyltransferases (UGTs) and, more impactfully, the physiological rationale for the resulting glycosylation. The work is solid, although the results are somewhat inconclusive in terms of identifying the exact biosynthetic roles of the UGTs, insight is provided regarding physiological role, which is clearly indicated to be chemical sequestration of the phytotoxic aglycone 17-hydroxygeranyllinalool (HGT). Note that this is not detoxification per se, as claimed by the authors, but rather chemical sequestration, which is a perhaps subtle but important distinction. Nevertheless, the resulting HGT-DGTs exhibit obvious toxicity towards *Manduca sexta*, and it is a bit misleading (not to mention confusing) to term it detoxification.

RESPONSE: Thanks for the positive comment on our revised manuscript. The difference between detoxification and sequestration is indeed subtle. As shown in our manuscript, impairing glucosylation in the HGL-DTG pathway leads to symptoms of autoxicity, which are associated with the ectopic accumulation of the HGL aglycone. Infiltration of WT leaves with the latter at high concentrations to mimic the metabolic alterations observed in glucosylation-impaired lines recapitulates previously observed necrotic spots due to the toxicity of the aglycone. Based on these results, we infer that glucosylation in the HGL-DTG biosynthetic pathway contributes as biochemical mechanism of avoidance of the toxicity of this aglycone. This autoxicity avoidance is indeed based on a chemical sequestration mechanism. Accordingly, we agree that the term of detoxification can be confusing, as it would contradict the inherent toxicity of glucosylated HGL-DTGs for insect larvae. For this reason, and as recommended by this reviewer, we have changed the term "detoxification" to "chemical sequestration" or "autotoxicity avoidance" in different locations in the revised manuscript.

Point 2. The manuscript can be hard to follow at times for other reasons, particularly the results from the reported gene silencing, which were not entirely conclusive. While it is not immediately evident how this could be fully clarified,

it should be noted that the use of different nomenclature for what seem to be orthologous, and are obviously homologous, enzymes increases the difficulty of following the results. While an argument is made in the response for keeping the current names intact, this seems to be more for the authors convenience rather than any truly valid rationale, and really should be reconsidered. For example, UGT91T1 and the orthologous (but unfortunately entirely distinctly named) UDP-RT1), as well as the orthologous pairs UGT74P3/P4 from *N. attenuata* and *N. obtusifolia* and unfortunately named Na/NoUDP-GT2. This could be significantly improved by normalizing the nomenclature - e.g., all of these almost certainly could easily be fit into the UGT family nomenclature like UGT74P3/P4, which presumably would make it easier to distinguish orthologous pairs as well as distinct paralogs.

RESPONSE: We agree that a more systematic naming of the new genes with respect to orthology would make the large amount of gene silencing-specific data presented in this manuscript more understandable. Following the reviewer and editor's advice, we submitted NaUDP-GT2 and NoUDP-GT2 sequences to the UGT nomenclature committee in order to rigorously name these UGTs. The UGT committee provided the new designations as NaUGT74P5 and NoUGT74P6, respectively, which are now included in the revised manuscript and figures. In a similar effort to consistently name new UGTs reported in this manuscript, NoUDP-RT1, which is orthologous, and very likely functionally homologous to NoUGT91T1, is now named NoUGT91T1-like. These new UGT names have been updated throughout the main text and figures.

Point 3. In addition, it might be helpful if it was noted (perhaps in the Introduction - line 165?) that rhamnosylation requires prior glucosylation.

RESPONSE: As summarized in one of our previous studies (Heiling et al., 2016), all rhamnosylated HGL-DTG identified so far possess rhamnosyl moieties attached to glucose moieties (and no HGL-DTGs with only rhamnosyl units have been detected so far). In other words, rhamnosylation requires prior glucosylation. This point is now clearly mentioned in the Introduction to further help the interpretation of the HGL-DTG biosynthetic pathway results presented in the manuscript.

Point 4. Other helpful changes would include clarifying the transitions between descriptions of the various enzymes (e.g., in the introduction - line 165 - transitioning from apparent rhamnosyltransferases to glucosyltransferases; note that it should be clarified in the first sentence describing UGT91T1 and UDP-RT1 that the comparison was of empty vector to silenced lines).

RESPONSE: We significantly reworked the last paragraph of the Introduction dealing with the characterization of the different enzymes and more rigorously reported, throughout the text, to which corresponding controls comparisons were made. The consistent renaming of UGT proteins in the two studied species also provides additional clarity to the enzyme descriptions.

Reviewer #6:

This paper describes a very impressive and detailed characterization of UDP-sugar dependent glycosylation of 17-hydroxygeranyllinalool in *Nicotiana attenuata*. Beyond the overall gene discovery, molecular and biochemical characterization of three genes and two enzymes, the paper explores the role of diterpene glycosylation in the control of auto-toxicity of this diterpene defense metabolite system. The paper is a revised submission. I was not one of the original reviewers, but I did read the response to the original reviews. I believe the authors addressed the major concerns of the original reviews. I only have a few relatively minor comments that can be addressed with editorial revisions and perhaps an additional reference.

Point 1. While UGT74P3 and UGT74P4 enzymes were functionally characterized using in vitro assays, characterization of UGT91T1 did not include such assays. This reviewer appreciates the difficulty of characterizing rhamnosyltransferases, since UDP-rhamnose (UDP-Rha) is - to the best of my knowledge - not commercially available. As the authors mentioned on page 19 (lines 581-584), a few labs have been successful in overcoming the issue of producing UDP-Rha. A recent study by Irmisch et al. (2018) The Plant Cell 30: 1864-1886 should perhaps be included as a reference for an efficient enzymatic system to produce UDP-Rha and its use for UGT characterization. Although I am not suggesting additional experiments for this present paper, the authors could contact Irmisch and co-workers for assistance with enzymatic UDP-Rha synthesis if needed for future work.

RESPONSE: We thank the reviewer for pointing our attention to the study by Irmisch et al. Reference is now made

to this study in discussing the availability of synthetic UDP-Rha for rhamnosyltransferase characterization and the enzyme system described for UDP-Rha production. We also note the suggestion of the reviewer to contact Irmisch and co-workers for assistance with UDP-Rha synthesis for future work.

Point 2. Page 8, line 227-236: The authors correctly highlighted that few rhamosyltransferases have been characterized. I would therefore suggest that the flavonol rhamnosyltransferase described by Irimsch et al. (2018) (see above) be included in the comparison described here and illustrated in Supplemental Figure S6.

RESPONSE: We added CcUGT77B2 characterized in the study by Irmisch et al. to our phylogenetic reconstruction. As previously observed in the latter study, this rhamnosyltransferase clusters is part of the UGT clade F (with the two other UGTs described in Irmisch et al. (2018)), unlike the rhamnosyltransferases characterized in our study, which are part of clade A. As such, UGT77B2 clusters apart of ours and other rhamnosyltransferases used as input in the phylogeny of characterized UGTs presented in Supplemental Figure S6.

Point 3. P17, line 519-529. It says "We identified three novel UGTs ...". The following lines only mention two, UGT74P3 and UGT74P4. The third UGT, UGT91T1, should also find mention in this paragraph. Again, this is a very impressive paper.

RESPONSE: Thanks for highlighting this discrepancy. This is now corrected in the revised manuscript.

TPC2020-RA-00165R1 2nd Editorial decision – *acceptance pending* Jan. 22, 2021

We are pleased to inform you that your paper entitled "Specific decorations of 17-hydroxygeranyllinalool diterpene glycosides solve the autotoxicity problem of chemical defense in Nicotiana attenuata" has been accepted for publication in The Plant Cell, pending a final minor editorial review by journal staff. At this stage, your manuscript will be evaluated by a Science Editor with respect to its presentation of scientific content, compliance with journal policies, and presentation for a broad readership. The Plant Cell has appointed several Ph.D. Plant Scientists to serve as Science Editors in this capacity, and you will soon receive additional information on this process.

Final acceptance from Science Editor Feb. 3, 2021
